# Large-scale ocean connectivity and planktonic body size

**DOI:** 10.1038/s41467-017-02535-8

**Published:** 2018-01-10

**Authors:** Ernesto Villarino, James R. Watson, Bror Jönsson, Josep M. Gasol, Guillem Salazar, Silvia G. Acinas, Marta Estrada, Ramón Massana, Ramiro Logares, Caterina R. Giner, Massimo C. Pernice, M. Pilar Olivar, Leire Citores, Jon Corell, Naiara Rodríguez-Ezpeleta, José Luis Acuña, Axayacatl Molina-Ramírez, J. Ignacio González-Gordillo, Andrés Cózar, Elisa Martí, José A. Cuesta, Susana Agustí, Eugenio Fraile-Nuez, Carlos M. Duarte, Xabier Irigoien, Guillem Chust

**Affiliations:** 1 AZTI, Marine Research Division, Txatxarramendi ugartea z/g, 48395 Sukarrieta, Bizkaia Spain; 2 College of Earth, Ocean and Atmospheric Sciences, Oregon State University, Corvallis, 97331 USA; 3 Institute for the Study of Earth, Oceans, and Space, University of New Hampshire, Durham, NH 03824-3525 USA; 4 Institut de Ciències del Mar, CSIC, 08003 Barcelona, Catalunya Spain; 5 Department of Ecology, Environment and Plant Science, University of Stockholm, Stockholm, 114 18 Sweden; 6 Departamento de Biología de Organismos y Sistemas, Universidad de Oviedo, Calle Catedrático Valentín Andrés Alvarez, sin número, 33006 Oviedo Spain; 7 Center for Environment, Fisheries and Aquaculture Science CEFAS Marine instrumentation and survey team Parkfield road, Lowestoft, Suffolk, NR330HT UK; 8 Departamento de Biología, Facultad de Ciencias del Mar y Ambientales, Universidad de Cádiz, Campus de Excelencia Internacional del Mar (CEI-MAR), E-11510 Puerto Real, Cadiz Spain; 9 Instituto de Ciencias Marinas de Andalucía, CSIC, Campus de Excelencia Internacional del Mar (CEI-MAR), E-11510 Puerto Real, Cadiz Spain; 10 King Abdullah University of Science and Technology (KAUST), Red Sea Research Center, Thuwal, 23955-6900 Saudi Arabia; 11 Department of Global Change Research, IMEDEA (UIB-CSIC), Instituto Mediterráneo de Estudios Avanzados, Esporles, 07190 Spain; 12 Instituto Español de Oceanografía, Centro Oceanográfico de Canarias, Santa Cruz de Tenerife, 38180 Spain; 13 IKERBASQUE, Basque Foundation for Science, Bilbao, 48013 Spain

## Abstract

Global patterns of planktonic diversity are mainly determined by the dispersal of propagules with ocean currents. However, the role that abundance and body size play in determining spatial patterns of diversity remains unclear. Here we analyse spatial community structure - β-diversity - for several planktonic and nektonic organisms from prokaryotes to small mesopelagic fishes collected during the Malaspina 2010 Expedition. β-diversity was compared to surface ocean transit times derived from a global circulation model, revealing a significant negative relationship that is stronger than environmental differences. Estimated dispersal scales for different groups show a negative correlation with body size, where less abundant large-bodied communities have significantly shorter dispersal scales and larger species spatial turnover rates than more abundant small-bodied plankton. Our results confirm that the dispersal scale of planktonic and micro-nektonic organisms is determined by local abundance, which scales with body size, ultimately setting global spatial patterns of diversity.

## Abstract (web summary)

Body size is hypothesised to be a major determinant of β-diversity in passively-dispersing marine organisms. Here, Villarino et al. show that plankton body size determines rates of dispersal along marine currents, with shorter dispersal and higher species spatial turnover in larger organisms.

## Introduction

The oceans represent the largest continuous environment on Earth. Over long timescales, all marine ecosystems are connected to each other by ocean currents^1^. However, biological connectivity, or the exchange of individuals across geographically separated subpopulations^2^, is not uniform as there exist barriers to dispersal. Such barriers include not only land masses, but also persistent frontal features at a range of spatial scales, sharp environmental gradients, and other oceanographic features^3^. Further, dispersal along ocean currents and the effect of these ‘physical barriers’ varies across taxa. In particular, as seen in terrestrial examples^4,5^, differences in body size and abundance amongst taxa are hypothesized to play a major role in determining both the distributional patterns and the scale of dispersal for marine planktonic species^6–9^. In order to understand how marine biodiversity is maintained locally and structured spatially^10^, it is therefore necessary to investigate the relationship between planktonic dispersal, body size, and local abundance^9,11^.

The shift in species composition among locations^12^, or β-diversity, is strongly influenced by environmental heterogeneity and seascape features, such as differences in temperature or geographic distance^13^. The scale-dependence of β-diversity can be described as a ‘distance*-*decay’ rate, measured as the slope of a linear relationship between the logarithm of community similarity and the logarithm of geographic distance among pairs of sites^14^. In both oceanic and terrestrial ecological communities, distance-decay patterns are set by three major mechanisms^15,16^: (1) local niche-based processes, which are summarized by the statement that, below 1-mm body size, “everything is everywhere, but the environment selects”^7,17,18^; (2) the effects of dispersal limitation, as hypothesized by the neutral theory of biodiversity^19^; these effects lead to a negative relationship between community similarity and geographic distance, even in a completely homogeneous environment; and (3) the spatial configuration of the seascape, which can also dictate the rate at which organisms disperse among sites^1^. It is a major challenge to elucidate which of these mechanisms is dominant for any given ecological community, since differences in key environmental characteristics are often strongly correlated with geographic distance^8,19,20^. Indeed, while distance-decay patterns have been observed for specific taxa in terrestrial (e.g., rainforest trees^21^), freshwater (e.g., aquatic beetles^22^; fish and macroinvertebrates^23^), and marine communities (e.g., coral reefs^19^; marine bacteria and prokaryotes^24,25^; and macrobenthos and plankton^26^), few studies have identified a robust distance-decay pattern across taxa or across key physiological traits, such as body size^27^.

Body size is the dominant physiological factor determining individual metabolic rates^28^ and, according to the metabolic theory of ecology^29^, it also controls numerous ecological processes. For example, smaller organisms have higher metabolic rates, faster growth rates, shorter generation times, and higher energy needs relative to larger organisms. Small organisms are generally more abundant, in terms of population density, than larger organisms^28^. This means that smaller organisms are expected to have lower local extinction rates^30^ and, therefore, reduced demographic stochasticity and ecological drift^19^ compared to larger organisms. Importantly, among smaller, mostly passively dispersed taxa, body size is expected to be inversely correlated with the spatial scale of dispersal. In fact, dispersal limitation has been hypothesized to increase with body size in planktonic communities^7,11^. In the oceans, therefore, smaller planktonic organisms, which are relatively more abundant, are expected to disperse farther with oceanic currents^6^, leading to shallower distance-decay slopes than those of larger planktonic organisms^7,8,24^.

Here we have quantified empirically derived distance-decay slopes and measured dispersal scales for a number of planktonic and micro-nektonic organisms, spanning a wide range of body sizes and abundances, from prokaryotes to small mesopelagic fishes. With these analyses, we have tested the hypothesized size-dependence of community dispersal scales and resulting spatial patterns of regional connectivity. To do so, we first explored the importance of surface ocean transit times, derived from previous Lagrangian particle simulations^1^, in explaining spatial patterns of β-diversity for each biological group, accounting for the relative contribution of environmental filtering^31^. Since β-diversity is controlled by surface ocean transit time, we then used the distance-decay slopes of each biological group to infer the community dispersal scale as a proxy of distribution range (sensu biogeography). These analyses are based on samples of pelagic communities collected across the subtropical and tropical ocean during the Malaspina 2010 Circumnavigation Expedition^32^. Our results show that the species composition of plankton and micro-nekton communities in tropical and subtropical open ocean is in large part determined by oceanic currents. Given this finding, we also explored the dispersal scale of each biological group and found a negative relationship between dispersal scale and body size: less abundant large-bodied plankton and micro-nekton communities in near-surface epipelagic waters show significantly shorter dispersal scales and larger spatial species-turnover rates compared to more abundant small-bodied plankton.

## Results

### Community assembly contributors

We find that the relative influence of surface ocean transit times and differences in environmental factors on plankton and micro-nekton community structure vary among groups (Mantel tests Table 1, Supplementary Fig. 1). For example, planktonic community β-diversity is significantly correlated with surface ocean transit times in all groups, explaining on average 22% of the variance (Table 1). Correlations with environmental distances are only significant for Cercozoa and myctophids, explaining 6–8% of the variance. In these two groups, the correlation between β-diversity and surface ocean transit times remains significant after controlling for environmental factors (Table 1). We also find low-shared covariation between environmental distance and surface ocean transit times, indicative of the low-spatial autocorrelation in oceanic factors. In fact, the correlation between the surface ocean transit times and the environmental distance among the all pair sites is rather weak (Mantel *r* = 0.09, Supplementary Table 1). A large fraction of the β-diversity variance remains unexplained by the selected explanatory factors (multiple regression on distance matrices, Table 1). This finding reflects the complexity of interacting mechanisms controlling spatial community assembly in the oceans. In addition, we find no relationship between the relative contribution of environmental drivers and body size (non-parametric bootstrap, *p*-value >0.05, Supplementary Table 2).

**Table 1:** Correlations between community similarity with currents and environmental factors

Biological groups	Mantel correlation			Mantel partial correlation	MRM
	*N* pairs	Ocean transit time	Environmental distance	Ocean transit time partialling out environmental distance	Ocean transit time + environmental distance
Prokaryotes	120	0.28**	0.02	—	0.29**
Small heterotrophic flagellates	112	0.30**	0.04	—	0.31**
Green algae	112	0.27**	0.04	—	0.28**
Fungi	89	0.13**	0.02	—	0.13**
Microbial eukaryotes ALL	112	0.24**	0.005	—	0.24**
Parasites	112	0.23**	0.002	—	0.23**
Cercozoa	107	0.10**	0.06*	0.07**	0.12**
Large flagellates	112	0.19**	0.08	—	0.22**
Coccolithophores 0–160 m	133	0.28**	0.01	—	0.28**
Diatoms 0–160 m	133	0.21**	0.02	—	0.22**
Diatoms surface	93	0.17**	0.04	—	0.18**
Dinoflagellates 0–160 m	133	0.21**	0.004	—	0.21**
Dinoflagellates surface	112	0.11**	0.04	—	0.12*
Mesozooplankton 0–200 m	36	0.40**	Not available	—	Not available
Gelatinous zooplankton	61	0.11**	0.04	—	0.11**
Macrozooplankton	65	0.23**	0.05	—	0.27**
Myctophids	95	0.32**	0.08**	0.20**	0.32**

Mantel correlations and Multiple Regression on distance Matrices (MRM) between β-diversity (i.e., community variation in space), environmental distance, and pair-site ocean transit times; and Mantel partial correlations after controlling for the effects of environmental distance, in statistically significant cases. *N* pairs: number of pair-sites considered at each group. The statistical significance of comparisons is assessed using Mantel and partial Mantel tests based on Pearson’s product moment correlation using 9999 permutations

*p-value ≤0.05; **p-value ≤0.01

### Community dispersal scales and spatial turnover

**Fig. 1:**
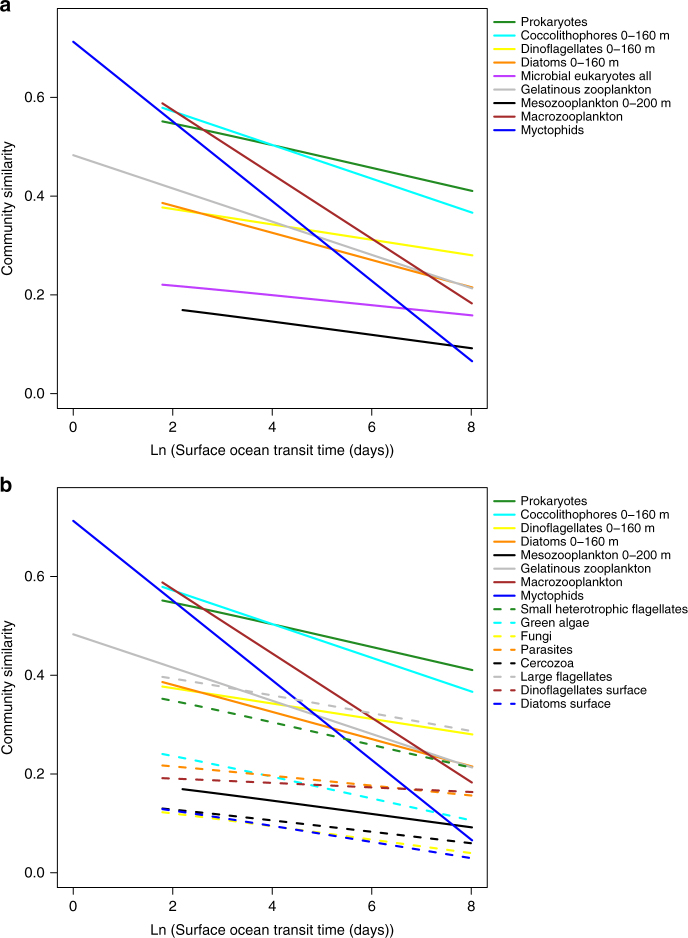
Time-decay between community similarity and surface ocean transit time. Time-decay relationship between community similarity using Jaccard similarity index and surface ocean transit time. Each line represents the community similarity decay between the species similarity and the logarithm of the surface ocean transit times for each biological group. **a** Main biological groups: prokaryotes (green); coccolithophores 0–160 m (cyan); dinoflagellates 0–160 m (yellow); diatoms 0–160 m (orange); microbial eukaryotes all (purple); gelatinous zooplankton (gray); mesozooplankton 0–200 m (black); macrozooplankton (brown); myctophids (blue). **b** All biological groups: prokaryotes (solid green); coccolithophores 0–160 m (solid cyan); dinoflagellates 0–160 m (solid yellow); diatoms 0–160 m (solid orange); mesozooplankton 0–200 m (solid black); gelatinous zooplankton (solid gray); macrozooplankton (solid brown); myctophids (solid blue); small heterotrophic flagellates (dashed green); green algae (dashed cyan); fungi (dashed yellow); parasites (dashed orange); cercozoa (dashed black); large flagellates (dashed gray); dinoflagellates surface (dashed brown); diatoms surface (dashed blue)

**Fig. 2:**
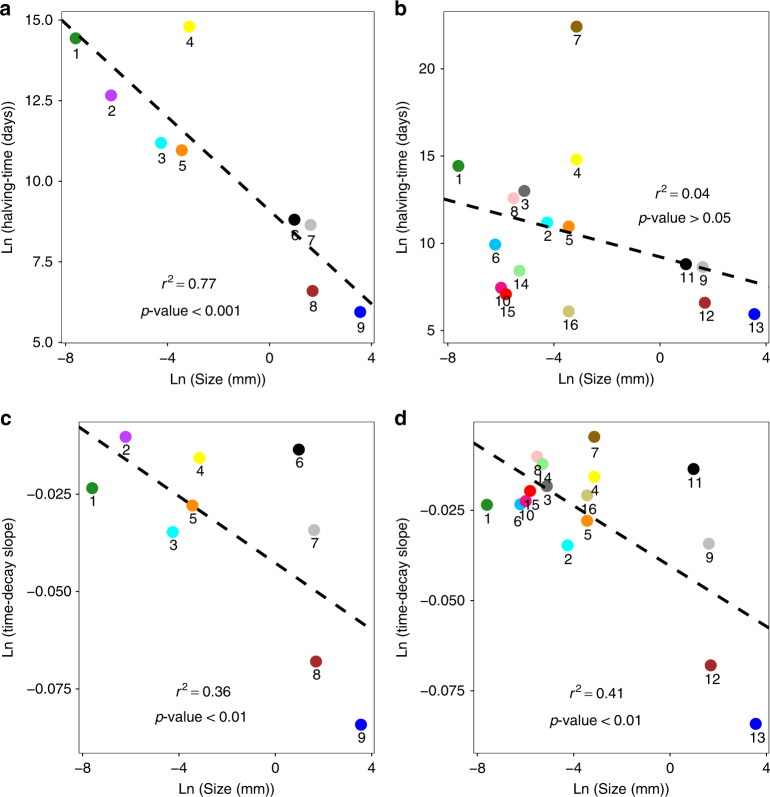
Correlations between halving-time and time-decay slope with body size and local abundance. **a** Correlation between the logarithms of halving-time (*y*) and body size (*x*) in main biological groups; linear regression equation *y* = 9.093–0.724*x*, *n* = 9, *r*^2^ = 0.767, *p*-value <0.001. **b** Correlation between the logarithms of halving-time (*y*) and body size (*x*) in all biological groups; linear regression equation *y* = 9.223–0.406*x*, *n* = 16, *r*^2^ = 0.037, *p*-value >0.05. **c** Correlation between the logarithms of the time-decay slope (*y*) and body size (*x*) in main biological groups; linear regression equation *y* = −0.042 − 0.004*x*, *n* = 9, *r*^2^ = 0.363, *p*-value <0.01. **d** Correlation between the logarithm of the time-decay slope (*y*) and body size (*x*) in all biological groups; linear regression equation *y* = −0.040 − 0.004*x*, *n* = 16, *r*^2^ = 0.406, *p*-value <0.01. Main biological groups **a**–**c**: prokaryotes (1); microbial eukaryotes all (2); coccolithophores 0–160 m (3); dinoflagellates 0–160 m (4); diatoms 0–160 m (5); gelatinous zooplankton (6); mesozooplankton 0–200 m (7); macrozooplankton (8); myctophids (9). All biological groups **b**–**d**: prokaryotes (1); coccolithophores 0–160 m (2); large flagellates (3); dinoflagellates 0–160 m (4); diatoms 0–160 m (5); small heterotrophic flagellates (6); dinoflagellates surface (7); parasites (8); gelatinous zooplankton (9); green algae (10); mesozooplankton 0–200 m (11); macrozooplankton (12); myctophids (13); cercozoa (14); fungi (15); diatoms surface (16). Ln Napierian logarithm. *P*-value is calculated at 95% of confidence interval in non-parametric bootstrap cross-validation. The dashed black line shows the model fit

**Fig. 3:**
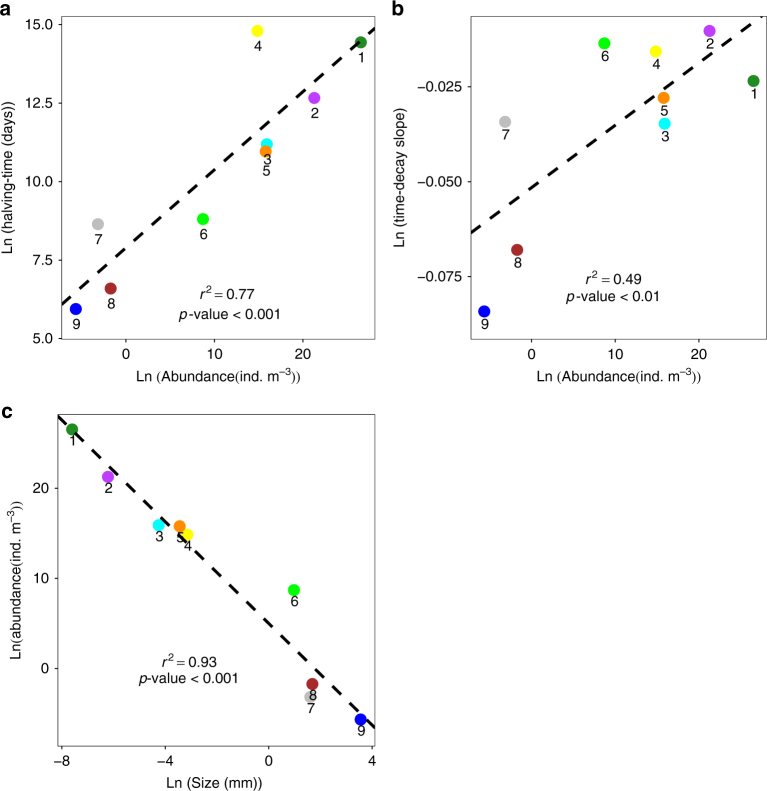
Correlations between halving-time and time-decay slopes with local abundance. **a** Correlation between the logarithms of halving-time (*y*) and local abundance (*x*) in main biological groups; linear regression equation *y* = 7.892 + 0.248*x*, *n* = 9, *r*^2^ = 0.766, *p*-value <0.001. **b** Correlation between the logarithms of the time-decay slope (*y*) and local abundance (*x*) in main biological groups; linear regression equation *y* = −0.051 + 0.001*x*, *n* = 9, *r*^2^ = 0.487, *p*-value <0.01. **c** Correlation between the logarithms of local abundance (*y*) and body size (*x*) in main biological groups; linear regression equation *y* = 5.002 − 2.820*x*, *n* = 9, *r*^2^ = 0.930, *p*-value <0.001. Main biological groups: prokaryotes (1); microbial eukaryotes all (2); coccolithophores 0–160 m (3); dinoflagellates 0–160 m (4); diatoms 0–160 m (5); mesozooplankton surface (6); gelatinous zooplankton (7); macrozooplankton (8); myctophids (9). Ln = Napierian logarithm. *P*-value is calculated at 95% of confidence interval in non-parametric bootstrap cross-validation

The significant negative relationship observed between oceanic transit times and β-diversity for planktonic and micro-nektonic organisms, more so than environmental distance, let us estimate community dispersal scales and spatial species turnover rates. The former is determined by means of the halving-time, that is, the oceanic transit time at which species similarity halves^16^; the latter comes from the slope of the distance-decay relationship for each group (Methods section; Fig. 1 and Table 2). In addition to the Mantel tests, the distance-decay slopes and the community similarity halving-times reinforce the result that community similarity decreases with the logarithm of surface ocean transit times (Fig. 1, Supplementary Fig. 2). For example, prokaryotes and microbial eukaryotes exhibit very long halving-times: 5094 and 866 years, respectively. In contrast, gelatinous zooplankton (15.5 years), myctophids (1 year) and macrozooplankton (2 years) show the shortest halving-times (Table 2). Likewise, the time-decay slopes are highest for large-sized groups, such as myctophids (−0.0807), macrozooplankton (−0.0657), and gelatinous zooplankton (−0.0336) (Fig. 1a, Table 2). Myctophids and macrozooplankton show very high initial similarity between neighboring stations, denoting a high-spatial dependence in community structure compared to smaller organisms (Fig. 1a, Table 2). In contrast, the shallow time-decay slopes and long halving-times of prokaryotes and microbial eukaryotes indicate globally mixed distributions for these groups (Fig. 1a, b). These groups of small organisms show the highest local abundance values (prokaryotes = 3.30 × 10^11^ ± 4.10 × 10^10^ ind. m^−3^; microbial eukaryotes = 1.72 × 10^9^ ± 1.49 × 10^9^ ind. m^–3^), 8–10 orders of magnitude more abundant than larger organisms (macrozooplankton = 1.79 × 10^−1^ ± 2.5 × 10^−1^ ind. m^−3^; myctophids = 3.5 × 10^−3^ ± 1.9 × 10^−2^ ind. m^−3^) (Table 3). The hypothesized size-dependence of dispersal in planktonic and micro-nektonic organisms is supported by a significant negative log–log relationship between the organism size and halving-time and time-decay slope (Fig. 2, Table 4). As expected, we also find a strong significant negative correlation between the organism’s body size and its local abundance (*r*^2^ = 0.93; *p*-value <0.001) (Fig. 3c), as well as a significant positive log–log relationship between the local abundance and halving-time and time-decay slope (Fig. 3a, b, Table 4).

**Table 2:** Halving-times for each biological group

Biological groups	Logarithmic decay between species similarity and surface ocean transit time			Size range (mm)	Size mean (mm)	Sampling depth (m)	Habitat
	Slope (*c*)	*S*_0_	HT (years)				
Prokaryotes	−0.0232**	0.52	5094	0.0003–0.001	0.0005	0	E
Small heterotrophic flagellates	−0.0231**	0.34	56	0.0008–0.003	0.002	0	E
Green algae	−0.0222**	0.24	5	0.0008–0.003	0.0025	0	E
Fungi	−0.0194**	0.16	3	0.0008–0.003	0.003	0	E
Microbial eukaryotes all	−0.0102**	0.22	866	0.0008–0.003	0.002	0	E
Parasites	−0.0100**	0.22	802	0.0008–0.003	0.004	0	E
Cercozoa	−0.0121**	0.12	12.5	0.0008–0.003	0.005	0	E
Large flagellates	−0.0181**	0.39	1215	0.0008–0.003	0.006	0	E
Coccolithophores 0–160 m	−0.0341**	0.52	198	0.002–0.5	0.0142	0–160	E
Diatoms 0–160 m	−0.0275**	0.27	158	0.002–0.4	0.033	0–160	E
Diatoms surface	−0.0206**	0.17	1	0.002–0.4	0.033	0	E
Dinoflagellates 0–160 m	−0.0156**	0.35	7325	0.002–0.5	0.043	0–160	E
Dinoflagellates surface	−0.0046**	0.19	14,931,726	0.008–0.003	0.043	0	E
Mesozooplankton 0–200 m	−0.0135**	0.16	18	0.3–5	2.65	0–200	E
Gelatinous zooplankton	−0.0336**	0.38	15.5	>5	5	0	N
Macrozooplankton	−0.0657**	0.55	2	4–15	5.41	0	N
Myctophids	−0.0807**	0.47	1	20–110	35	0	M&N

Halving-times derived from species similarity and surface ocean transit times with logarithmic decay models. The logarithmic decay model shows the community similarity decline (slope) with the logarithm of surface ocean transit time

HT Halving-time, N neustonic, E epipelagic, M mesopelagic, *S*_o_ initial similarity

**p-value <0.01

**Table 3:** Local abundance of main biological groups

Main biological groups	Abundance ± SD (ind. m^−3^)
Prokaryotes	3.30 × 10^11^ ± 4.10 × 10^10^
Microbial eukaryotes all	1.72 × 10^9^ ± 1.49 × 10^9^
Coccolithophores 0–160 m	8.08 × 10^6^ ± 7.60 × 10^6^
Diatoms 0–160 m	7.16 × 10^6^ ± 1.30 × 10^6^
Dinoflagellates 0–160 m	2.80 × 10^6^ ± 1.80 × 10^6^
Mesozooplankton surface	6.00 × 10^3^ ± 1.09 × 10^4^
Gelatinous zooplankton	0.04235 ± 1.49 × 10^9^
Macrozooplankton	0.179 ± 0.251
Myctophids	0.0035 ± 0.0193

Mean abundance and standard deviation (ind. m^−3^) of main biological groups (microbial eukaryotes subgroup abundance is not determined)

**Table 4:** Correlations between halving-time and time-decay slope with body size and local abundance

Main biological groups (*n* = 9)	Statistic	Parametric model	Bootstrap
Ln (HT) vs Ln (size)	Confidence interval		(−1.067, −0.715)
	*p*-value	0.001	<0.001
	RMSE	1.542	
	Adjusted *r*^2^	0.767	
	Linear regression equation	*y* = 9.093 − 0.724*x*	
Ln (time-decay slope) vs Ln (size)	Confidence interval		(−1.180, −0.236)
	*p*-value	0.050	<0.01
	RMSE	0.020	
	Adjusted *r*^2^	0.363	
	Equation	*y* = −0.042 − 0.004*x*	
Ln (HT) vs Ln (abundance)	Confidence interval		(0.727, 1.057)
	*p*-value	0.001	<0.001
	RMSE	1.547	
	Adjusted *r*^2^	0.766	
	Equation	*y* = 7.892 + 0.248*x*	
Ln (time-decay slope) vs Ln (abundance)	Confidence interval		(0.359, 1.140)
	*p*-value	0.022	<0.01
	RMSE	0.018	
	Adjusted *r*^2^	0.487	
	Equation	*y* = −0.051 + 0.001*x*	

All biological groups (*n* = 16)	Statistic	Parametric model	Bootstrap
Ln (HT) vs Ln (size)	Confidence interval		(−0.709, 0.100)
	*p*-value	0.230	>0.05
	RMSE	4.199	
	Adjusted *r*^2^	0.037	
	Equation	*y* = 9.223 − 0.406*x*	
Ln (time-decay slope) vs Ln (size)	Confidence interval		(−1.244, −0.252)
	*p*-value	0.005	<0.01
	RMSE	0.016	
	Adjusted *r*^2^	0.406	
	Equation	*y* = −0.040 − 0.004*x*	

Evaluation of the log–log relationship between (i) group size vs halving-time (HT) and time-decay slope, and between (ii) local abundance vs halving-time and time-decay slope in main- and all biological groups. The table shows parametric models (all observations included) and non-parametric bootstrap cross-validations (95% confidence interval)

Ln Napierian logarithm. RMSE root mean square error

### Community spatial clustering

**Fig. 4:**
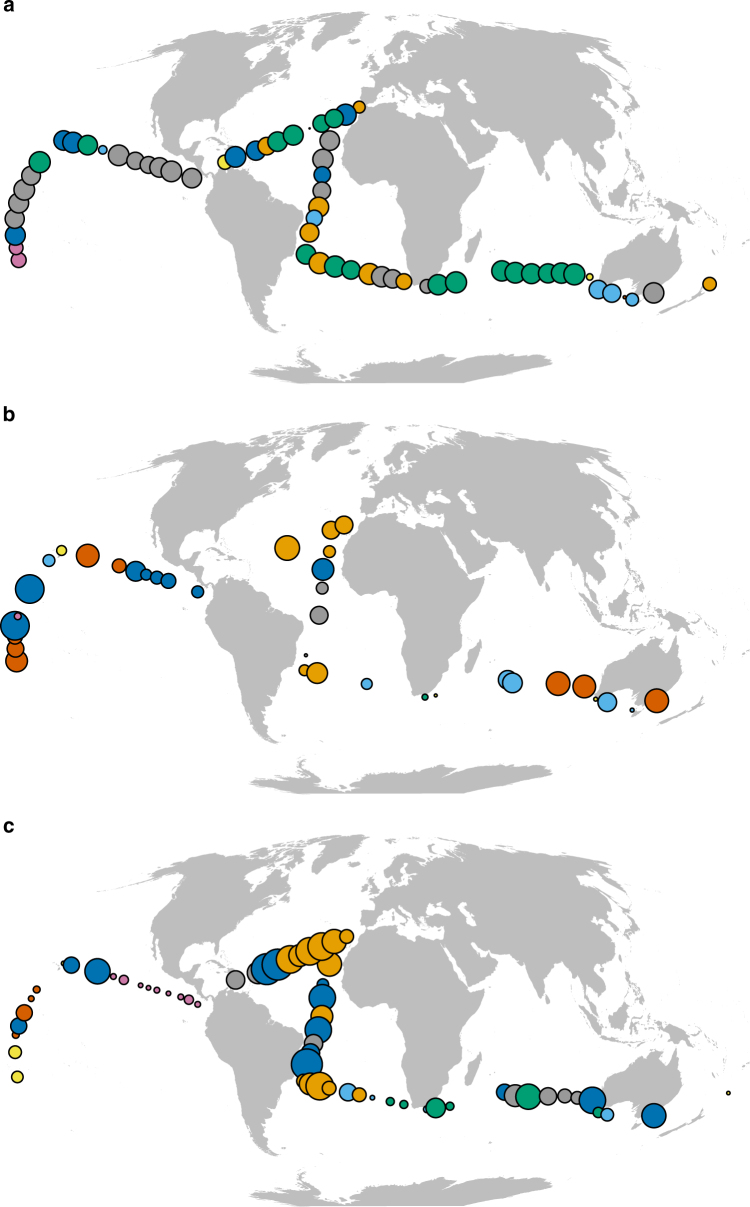
Spatial community patterns. Hierarchical clustering based on the Jaccard similarity index for, **a** diatoms 0–160 m, **b** mesozooplankton 0–200 m, and **c** myctophids. Each color represents a different hierarchical cluster. The size of stations indicates the number of connections (i.e., species/OTUs similarity between sites), that is, larger sized circles share more species (or OTUs) within all stations, compared to small sized circles. Some stations have been aggregated based on proximity for clarity

Spatially heterogeneous patterns in community similarity are observed in each size-group (Fig. 4). Specifically, hierarchical clustering^33^ of our estimates of community similarity reveals distinct spatial patterning of larger organisms, with clear biogeographic regions in the myctophid, meso- and macrozooplankton communities (Fig. 4b, c, Supplementary Fig. 3f). In these large-sized communities, connectivity is highest in the Atlantic Ocean and the southern Indian Ocean. Network graphs also reveal an area of high β-diversity for myctophids in the central Pacific Ocean (Fig. 4c, pink points), where species connectivity is low due to limited mixing between neighboring communities. Marine communities in the Pacific and Atlantic Oceans cluster into different groups, reflecting the barrier imposed by land (Fig. 4). A possible oceanographic barrier is also detected in the Hawaiian archipelago, dividing communities into two different groups at either side of the islands (Fig. 4c). In contrast to large-sized groups, small-sized groups show many different clusters of various sizes, randomly distributed over the global ocean, as seen, for example, in diatoms (Fig. 4a).

## Discussion

In our analysis, the spatial arrangements of the sampled assemblages reveal that surface ocean transit time explains a larger fraction of the variability in planktonic and micro-nektonic community similarity than do environmental factors. This indicates that passive dispersal with surface ocean currents—arguably an ecologically neutral process similarly affecting all planktonic and micro-nektonic organisms—is a stronger determinant of community structure than niche-filtering factors^19^. In addition, the low-spatial correlation found between oceanic transit time and environmental distance likely results from the global scale of our study (tropical and subtropical regions of the world’s oceans). Contrary to most regional studies where climate and space correlate well, here climatically very similar locations can be geographically far apart, for instance, two antipode points in the equator or two points at 30° North and South.

We have found that dispersal limitation in small (0.0003 to ca. 10 mm) abundant planktonic and micro-nektonic organisms increases with body size. This is based on a trend toward steeper time-decay slopes and shorter halving-times with increasing body size. Notably, the large halving-times of marine microbial organisms imply that, when dispersing with ocean currents, it would take thousands of years of oceanic transport for such communities to halve the similarity between adjacent sampling stations (i.e., the initial similarity). However, in some biological groups, such as surface dinoflagellates, community similarity is never less than half of the initial similarity, even for stations located far apart. As such, the halving-time is a relative indicator, or proxy, of community dispersal scale, and should not be interpreted as an absolute value of the transit time that operates among the sub-communities. Therefore, communities of small organisms (body size <2 mm) and high-local abundance are likely to have a panmictic worldwide distribution^7,11^. On the other hand, larger-sized organisms exhibit stronger spatial patterning^6,33^ and need only a few decades of surface ocean transit time, ~20 years at most, to halve their initial similarity. This means that for these large-sized organisms, species will be similar at geographically proximate locations, and dissimilar between distant locations. These results highlight that patterns of β-diversity in open-ocean in planktonic and micro-nektonic organisms are size-dependent^34^. In order to explain the underlying process of this empirical finding, we have identified a significant positive relationship between the local abundance and the community dispersal scales. This was expected since local abundance scales negatively with body size^29,35^, as confirmed in our data. Moreover, generation time also scales negatively with body size^29,35^. Locally abundant species are exposed to lower local extinction rates^30^ and hence, reduced demographic stochasticity and ecological drift^19^. Therefore, we suggest that large population densities and short generation times of micro-planktonic organisms are the mechanisms explaining the larger geographic range and relatively weak spatial structure of these organisms^30,34,36,37^. In contrast, larger planktonic organisms generally have longer generation times and smaller population densities^38^, and are therefore more sensitive to local extinctions and ecological drift, resulting in stronger spatial structure. In addition, lower sinking losses^39^ and longer survival times of resting stages of small passively dispersed plankton (from prokaryotes to phytoplankton)^40^ allow their populations to travel greater distances than large-sized plankton.

In our study, the environment, through environmental species sorting, explains little of the observed spatial variation in community structure in both plankton and micro-nekton groups. There are multiple plausible explanations for this finding. First, the Malaspina sampling was restricted to tropical and subtropical regions and took place in summertime, when horizontal environmental gradients are typically low at surface. As a result, it is difficult to capture assemblage variations due to climate. Second, the presence-absence indices that we used are less sensitive^41^ compared to relative abundances. We anticipate that the latter indice would potentially identify a stronger relationship in both small and large-sized plankton and micro-nekton with environmental gradients. Other potential reasons might stem from the other environmental variables not measured in our study, and the exclusion of biotic variables, which might play a role driving spatial distribution, particularly in large planktonic taxa. Finally, marine microbial communities are mainly dispersed by advection and diffusion. These, together with their relatively high-niche plasticity compared to the plasticity of larger-bodied taxa, results in microbes showing broad spatial distributions. However, our results do not identify low niche plasticity in large-bodied taxa^42^, and we observe no significant relationship between organism body size and environmental variability. This is in line with a recent meta-analysis by Soininen^41^ that concluded that body size and environmental species sorting are not significantly related in a data set spanning a range in body size of up to 12 orders of magnitude. This apparent contradiction in thinking and evidence highlights the need for further research on the strength of environmental species sorting among organisms of different size.

In addition to passively dispersed planktonic organisms, we also analysed connectivity in myctophid fish communities (micro-nekton), which are active swimmers. The myctophid group showed short dispersal scales and a steep distance-decay slope comparable with those of other large-bodied passive dispersers (i.e., gelatinous zooplankton and macrozooplankton). This evidence of dispersal limitation for myctophids is likely a result of their migration patterns being mostly vertical (rather than horizontal), as they move daily between the mesopelagic and epipelagic zones^43^. In contrast, numerous marine megafauna, such as large pelagic fish and marine mammals, actively move horizontally, either to forage for food or to complete long-distance migration^44^. Indeed, previous research has demonstrated a positive relationship between dispersal distance and body size for such megafauna^45^. For myctophids, horizontal movement occurs predominantly as larvae, with passive transport by ocean currents in epipelagic waters^43^. The observed similarity in dispersal patterns of myctophids and macrozooplankton may thus arise from the same processes: passive horizontal dispersion of larvae, with movement as juvenile and adults mainly devoted to diel vertical behavior. It is worth noting that contradictory results have been found in a study by Jenkins et al.^9^, whose findings suggested that body size controls the dispersal of active dispersers, but not of passive dispersers like planktonic organisms. However, this study^9^ did not characterize the full range of body sizes that we have studied, and therefore is limited in its scope. Our data support the existing understanding that β-diversity in the pelagic domain increases with body size in small and mainly passive organisms but decreases in actively mobile larger taxa (pelagic fishes, cetaceans), because high-dispersal capacity reduces compositional differences between sites^27^. Furthermore, given that the community dispersal scale defined here is a good proxy for the geographic range of a particular community, it seems that the local abundance of the species from an ecological guild relates positively to their geographic range in plankton, similar to many other groups from marine and terrestrial domains, including both passively and actively dispersing species^46^.

The spatial distribution of community similarity, identified using hierarchical clustering, revealed distinct size-dependent spatial patterns. In particular, we identified large-scale frontal zones as areas of low β-diversity in the case of mesozooplankton and especially myctophid fishes. These frontal zones act as barriers separating subtropical gyres and are typically areas of relatively high-primary production^47^. Limited dispersal between distinct pelagic provinces has been shown to play a major role in plankton population differentiation, and in the creation of strong genetic breaks and enhanced diversity in bridging regions^48^. Another interesting conclusion drawn from these network maps is that modeling results of global ocean transit times indicate that the Atlantic Ocean is less connected than are the Pacific and Indian Oceans. This is mirrored in the spatial clustering of planktonic organisms found in our data, particularly in myctophids and macrozooplankton, where a set of unique clusters are only seen in the Atlantic Ocean (orange-color stations), and another set of unique clusters (pink and dark-orange stations) only in the Pacific and Indian Oceans.

In summary, we have shown that planktonic and micro-nektonic β-diversity declines logarithmically with surface ocean transit times, and that dispersal limitation is a more powerful determinant of community structure than is niche segregation in the tropical and subtropical open ocean. More importantly, we have identified that large-bodied plankton groups and neustonic-migrating mesopelagic myctophid fishes have shorter dispersal scales and higher species spatial turnover rates when compared to more abundant micro-plankton groups. Together, these results highlight that body size, local abundance, and ocean currents are key determinants of global patterns of biodiversity in marine planktonic and small-bodied pelagic communities.

## Methods

### Data collection

The Malaspina Expedition sailed the subtropical and tropical Atlantic, Indian, and Pacific Oceans on board R/V *Hespérides*, with a balanced distribution sampling to characterize pelagic communities across the open ocean in the northern and southern hemisphere^32^. Samples included pelagic communities encompassing six orders of magnitude in body length, including prokaryotes (~0.0003 − 0.001 mm) and small microbial eukaryotes (~0.0008 − 0.003 mm), large microbial eukaryotes (i.e., phytoplankton (~0.002 − 0.5 mm), surface mesozooplankton (~0.2 − 3 mm) and epipelagic mesozooplankton (~0.3 − 5 mm), macrozooplankton (~4 − 15 mm), gelatinous zooplankton (>5 mm), and myctophid fishes (20−110 mm) (Supplementary Table 3). In this paper, we focus on the neuston, epipelagic, and neuston-migrating mesopelagic communities. Neuston communities include gelatinous zooplankton, macrozooplankton, and mesozooplankton (surface) occupying the first centimeters of surface ocean. Epipelagic communities include mesozooplankton (epipelagic), phytoplankton divided as diatoms, coccolithophores and dinoflagellates, and prokaryotes and small microbial eukaryotes living in the first 200 m of the water column. Mesopelagic communities include myctophid fishes found in the neuston layers during their nightly migration (Supplementary Table 3).

At each sampling location, ~12 L of seawater was used to determine the composition of microbial communities (marine prokaryotes and small microbial eukaryotes). Water samples were pre-filtered through a 200 μm mesh to remove large plankton, followed by sequential filtration, involving filtering the sample through a 20-µm Nylon mesh followed by a 3 µm pore-size polycarbonate filter (Poretics), and finally through a 0.2 µm polycarbonate filter (Poretics) using a peristaltic pump (MasterFlex 7553-89 with cartridges Easy Load II 77200-62, Cole-Parmer Instrument Company) to collect the prokaryotes and small eukaryotes (size fraction: 0.0003 − 0.001 mm). The filters were then flash-frozen in liquid N_2_ and stored at −80 °C until DNA extraction. Water samples for nano- and micro-autotrophic plankton (for simplicity, hereafter ‘phytoplankton’) determination were taken from surface waters (3 m) using a 30 L Niskin bottle, and from the depth receiving 20% of the light (PAR) incident just below the surface, and the depth of the chlorophyll maximum, using a Rosette sampler system fitted with 24, 10 L Niskin bottles and a SeaBird CTD sensor. The water was introduced in glass bottles that were hermetically capped after fixation with hexamine-buffered formaldehyde solution (4% final formalin concentration)^49^. Gelatinous zooplankton, macrozooplankton, surface mesozooplankton and myctophid fish were sampled using a neuston sampler (80 cm wide, 30 cm high) fitted with a 200 µm mesh size, towed at 2–3 knots during 10−15 min at a depth of 15 cm and a distance of 5 m from the starboard side of the hull^50^. Deeper mesozooplankton communities (0–200 m) were sampled with a multi-net (300−5000 µm mesh size).

### Species identification

Traditional taxonomy approaches were used to identify species of phytoplankton^49^, gelatinous zooplankton^50^, surface mesozooplankton, and juvenile and adult stages of myctophids^51^ (Supplementary Table 3). For phytoplankton examination, 100 mL aliquots of sample were settled in composite samples and observed under an inverted microscope, following the Utermöhl method^52^. At least two transects of the chamber bottom were examined under high magnification (×312) to count the smaller cells, and the whole chamber bottom was scanned at ×125 to enumerate the larger, less frequent forms. Large phytoplankton (dinoflagellates, diatoms and coccolithophores) were identified using inverted microscopy to species level when possible. However, some taxa could only be identified to genus (e.g., *Thalassiosira* spp.) or to more general categories like ‘Small dinoflagellates’ or ‘Small coccolithophores’^49^. Gelatinous zooplankton were identified combining morphological taxonomical approaches and high-resolution photography^50^ (Supplementary Table 3). The use of molecular approaches in gelatinous zooplankton has many gaps, and the most common markers used in techniques, such as DNA barcoding like COI or ITS are often not useful in resolving all gelatinous phyla^53^. We confirmed some morphological identifications using mainly DNA barcode with COI as molecular marker. However, in groups like Ctenophora or in thaliaceans the identification approach was based solely on morphology because the molecular markers were not valid to differentiate between species^53^. Myctophids and surface mesozooplankton were identified using morphometric and morphological parameters^54^. Metabarcoding was used to identify macrozooplankton, epipelagic mesozooplankton (0−200 m), and microbial communities (prokaryotes and microbial eukaryotes) (Supplementary Table 3). Specifically, DNA from macrozooplankton (crustacean, mollusks, and insects) was extracted as in Marco-Herrero et al.^55^. Target mitochondrial DNA from the 16S rRNA and COI genes was amplified with polymerase chain reaction (PCR). Primers 1472 (5′-AGATAGAAACCAACCTGG-3′)^56^ and 16L2 (5′-TGCCTGTTTATCAAAAACAT-3′)^57^ were used to amplify 540 bp (base pair) of 16S, while primers COH6 (5′-TADACTTCDGGRTGDCCAAARAAYCA-3′) and COL6b (5′-ACAAATCATAAAGATATYGG-3′)^57^ allowed amplification of 670 bp of COI. The PCR products were sent to external laboratories to be purified and then bidirectionally sequenced (Sanger). Sequences were edited using the Chromas software version 2.0 (http://technelysium.com.au/wp/chromas/). With the final DNA sequences obtained, a BLAST search was executed on the NCBI webpage (https://www.ncbi.nlm.nih.gov/) to get the sequence that matched best. Macrozooplankton specimens were identified at species level when sequences fit 100%. Assignations to generic or familial level were made with a 90–99% divergence, depending on taxa and genes analysed^58^. For lower % of divergence Operational Taxonomic Units (OTUs) were kept without taxonomical adscription. DNA from mesozooplankton (0–200 m) samples was extracted following Corell and Rodriguez-Ezpeleta^59^. The V4 of the 18S rRNA gene was amplified using the #1/#2RC primer pair^60^ following the ‘16S Metagenomic Sequence Library Preparation’ protocol (Illumina, California, USA). Amplicons were purified using the AMPure XP beads, quantified using Quant-iT dsDNA HS assay kit with a Qubit 2.0 Fluorometer (Life Technologies, California, USA) and pooled for high throughput sequencing in the Illumina MiSeq platform (Illumina, California, USA). After demultiplexing based on index, reads were trimmed at 200 bp (as overall Phred quality scores decreased after this position) and processed following the mothur^61^ MiSeq SOP^62^. Briefly, sequences with ambiguous bases, chimeras, and global singletons were removed, and OTUs were created by merging reads at 97% similarity. Prokaryotic diversity was assessed by amplicon sequencing of the V4–V5 regions of the 16S rRNA gene in the Illumina MiSeq platform (iTags) using paired-end reads (2 × 250 bp) and primers 515F-Y (5′-GTGYCAGCMGCCGCGGTAA-3′) and 926 R (5′-CCGYCAATTYMTTTRAGTTT-3′) targeting both Archaea and Bacteria^63^. Small microbial eukaryotic diversity was assessed by amplicon sequencing of the V4 region of the 18S rRNA gene with the Illumina MiSeq platform using paired-end reads (2 × 250 bp) and the universal eukaryotic primers TAReukFWD1 (5′-CCAGCASCYGCGGTAATTCC-3′) and TAReukREV3 (5′-ACTTTCGTTCTTGATYRA-3′)^64^. For both groups, sequence data processing was performed using an UPARSE^65^ based workflow implemented in a local cluster [Marbits platform, ICM] (Logares^66^). Briefly, raw reads were corrected using BayesHammer^67^ following Schirmer et al.^68^. Corrected paired-end reads were subsequently merged with PEAR^69^; sequences longer than 200 bp were quality-checked (maximum expected errors 0.5) and de-replicated using USEARCH^65^. OTU were delineated at 97% similarity using UPARSE V8.1.1756^65^. To obtain OTU abundances, reads were mapped back to OTUs at 97% similarity using an exhaustive search (-maxaccepts 20 -maxrejects 50,000–100,000). Chimera check and removal were performed both de novo and using the SILVA reference database^70^. Taxonomic assignation was performed by blasting (i.e., BLASTn^71^) the sequence representative of each OTU against the 16S SILVA v123^70^ and two in-house marine microeukaryote databases based in a collection of Sanger sequences^72^ or 454 reads from the BioMarKs project (http://www.biomarks.eu/). Analysis of macro-organisms was conducted at the species level, where possible, and that of mesozooplankton and heterotrophic prokaryotes and eukaryotes was conducted at the OTU level (Supplementary Table 3). Standard protocols for assignment to OTUs or species for each group differed slightly between groups depending on the taxa. However, the same approach was used for all stations in the Malaspina cruise and, thus, the among-site similarity of each group is consistent, independent of the exactness of OTU or species assignment.

Global estimates of abundance for each group were made using flow cytometer counting^73^ (prokaryotes), microscope epi-fluorescence counting (small microbial eukaryotes), inverted microscopy (phytoplankton), and stereo-microscope counting (macrozooplankton) (Supplementary Table 3). The abundance of phytoplankton (diatoms 0–160 m, coccolithophores 0–160 m and dinoflagellates 0–160 m) was vertically integrated (0–160 m). The abundance of myctophids, gelatinous zooplankton and macro- and surface mesozooplankton was estimated using traditional taxonomy identification techniques (Supplementary Table 3).

The above analyses produced a data set of nine focal organismal groups, with high sample spatial resolution and species occurrence (Supplementary Table 4): prokaryotes (120 stations and 1218 OTUs), microbial eukaryotes all (112 stations and 35615 OTUs), coccolithophores 0–160 m (133 stations and 47 species), dinoflagellates 0–160 m (133 stations and 236 species), diatoms 0–160 m (133 stations and 68 species), mesozooplankton 0–200 m (36 stations and 4283 OTUs), gelatinous zooplankton (61 stations and 12 species), macrozooplankton (65 stations and 46 species), and myctophids (95 stations and 12 species). Additionally, to infer the relationships between body size and plankton biological connectivity, and based on the taxonomic assignation of each OTU, we split the small microbial eukaryotes group into 8 subgroups labeled as: Small heterotrophic flagellates (1014 OTUs), Green algae (451 OTUs), Fungi (59 OTUs), Parasites (20466 OTUs), Cercozoa (84 OTUs), Large flagellates (375 OTUs) Dinoflagellates surface (8391 OTUs) and Diatoms surface (85 OTUs) (Supplementary Table 4).

### Distance and similarity matrices

In Supplementary Fig. 4, we show a general flow diagram with the steps we took to quantify the dispersal patterns of planktonic and micro-nektonic communities. Briefly, the analysis involves the calculation of three similarity or distance metrics for each pair of sampling locations, which over all pairs is stored as a matrix: biotic similarity, environmental distance, and surface ocean transit times (also a distance)^31^.

For the biotic similarity matrix, we calculated pairwise species similarities for each group using the Jaccard similarity (J) index^74^ with species presence-absence data to infer the variation of the species assemblages (β-diversity matrix):

1 $$J_{({i},{j})} = \frac{{a}}{{{a + b + c}}},$$

where *a* is the number of species shared between two sites (*i* and *j*), *b* is the total number of species that occur in site *i* but not in *j*, and *c* is the total number of species that occur in site *j* but not in *i*.

The environmental distance matrix was populated using a multidimensional Euclidean distance calculation, applied to a set of key environmental variables at surface pair-sites (Table 5). All key environmental variables were converted into *Z*-scores [(*x*-mean)/standard deviation] to give equal weight in the distance calculation. The environmental variables used here have been previously shown to be important to the spatial distribution of marine organisms^43,75^. Further, key environmental variables were selected using a BIOENV function in R, which finds a subset of environmental variables (from a larger super set), such that the Euclidean distances of scaled environmental variables have the maximum (rank) correlation with community dissimilarities^76^.

**Table 5:** Environmental variables used and model selection for explaining species similarity

Main biological groups	Environmental variables	BIOENV variable selection
Prokaryotes	*T*, *S*, O_2_, Conduct, Fluo, PARi, SPARi, Turb, Beam-att-1m, O_2_volt, *Z*_max_	O_2_, Turb, Beam-att-1m, *Z*_max_
Microbial eukaryotes ALL		Turb, *Z*_max_, O_2_
Coccolithophores 0–160 m	*T*, *S*, O_2_, Chl-a, Conduct, O_2_volt, Fluo, PARi, SPARi, Turb, Beam-att-1 m	SPARi
Dinoflagellates 0–160 m		SPARi
Diatoms 0–160 m		O_2_volt
Mesozooplankton 0–200 m	Not available	Not available
Gelatinous zooplankton	SST (remotely sensed), SST, *S*, Chl-a,*W*, *Z*	*S*
Macrozooplankton	*T*, *S*, O_2_, Chl-a, Conduct, O_2_volt, Fluo, PARi, SPARi, Turb, Beam-att-1 m	Turb, *S*
Myctophids	*T*, *S*, O_2_, *T*_400_, *T*_200_, *S*_400_, *S*_200_, O_2min_, SFluo, Fluo_max_	*T*_400_

Environmental variables and best BIOENV model selection for each of the different plankton groups.

SST sea surface temperature (°C), *S* salinity, O_*2*_ oxygen (ml/l), *O*_*2*_ volt oxygen (volts), *O*_2min_ oxygen minimum concentration (ml/l), Conduct conductivity (S/m), Fluo fluorescence (volts), Fluo_*max*_ maximum fluorescense (volts), SFluo surface fluorescense (volts), Turb turbidity (FTU), PARi photosynthetic active radiation irradiance (µE/(cm^2^sg)), SPARi surface photosynthetic active radiation irradiance (µE/(cm^2^sg)), Beam-att-1m beam attenuation coefficient at 1 m depth (m^−1^), *Z*_*max*_ maximum depth of sampling (m), Chl-a chlorophyll-a (µg/l), *W* wind (m/s), *Z* depth of station (m), *T*_400_ temperature at 400 m (°C), *T*_200_ temperature at 200m (°C), *S*_400_ salinity at 400 m, *S*_200_ salinity at 200 m

The surface ocean transit time matrix was built using estimates of minimum connection times or surface ocean transit times between pair-sites obtained from previously published global surface ocean Lagrangian particle simulations^1^. In brief, velocity fields from the ECCO2 model (http://ecco2.org), an eddy permitting global Ocean General Circulation Model (OGCM) with a 1/4° × 1/4° horizontal resolution that assimilates satellite and in situ data using a 4D-var approach, were used as inputs to the TRACMASS offline particle tracking framework^77^ to advect virtual particles, bound to the near surface (5–20 m depth), using only horizontal velocities. The particles (36 million in total) were seeded over 9 years and advected for 100 years by looping velocity fields from the years 2000–2010, with particle positions saved every 3 days. No extra diffusivity was added to the movement of the particles. When calculating minimum connection times, we aggregated the model’s ¼° × ¼° grid cells to 11,116 discrete 2° × 2° patches. The size and number of these connectivity patches were selected as a balance of computational feasibility and biogeographic detail. However, for many pairs of patches, direct estimates of minimum connection times were not available (due to the global scale of the simulations and the relatively short integration period). Therefore, a shortest path algorithm was applied to estimate minimum connection times between all pairs of patches^1^.

**Fig. 5:**
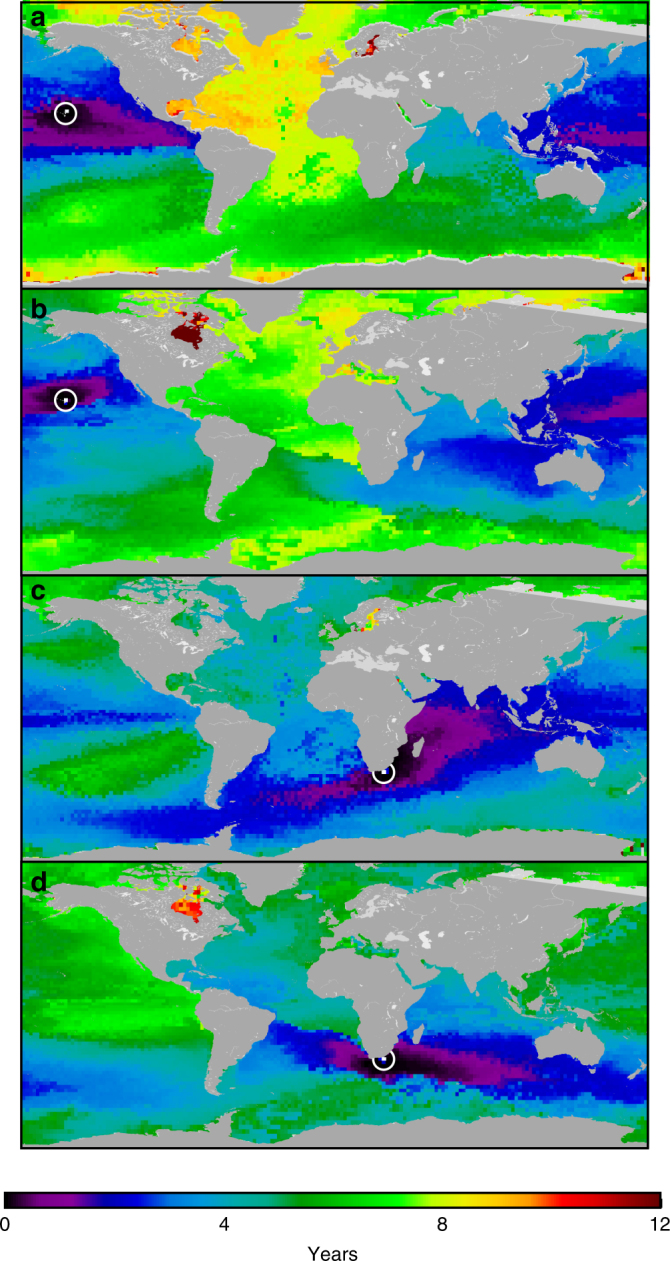
Surface ocean transit times. Examples of minimum connection times to and from two locations identified by white circle-dots in two random Malaspina stations: off Hawaii (**a**,** b**) and off South Africa (**c**,** d**). Times ‘to’ are the shortest times taken for water from other patches to arrive at these locations. Times ‘from’ are the shortest times taken for water from these locations to go to all others. Minimum ocean transit time values were generated using Dijkstra’s algorithm (Methods section)

To illustrate spatial patterns of minimum connection times (surface ocean transit times), we show times to, and times from, for two Malaspina sampling stations identified by white circle-dots in Fig. 5. Minimum transit times from these Malaspina sampling stations to nearby surface ocean locations are short relative to those to far-off locations (Fig. 5). These figures highlight the spatially heterogeneous nature of global surface ocean connectivity and dispersal. For example, the Atlantic Ocean is less connected, since the minimum connection times between the Atlantic and other oceans are longer compared to the connection times of the other oceans. It is important to note that dispersal scales estimated from modeled surface ocean transit times may be a good first-order approximation for planktonic organisms, but are less appropriate for larger biological taxa—particularly for myctophids, which exhibit active vertical migration. There are numerous alternatives to modeling the dispersal of actively swimming marine organisms, ranging from complex agent-based models to simple advection-diffusion methods. However, ocean transit times derived from passive surface-ocean Lagrangian particle simulations were sufficient for our study of global planktonic community structure.

### Correlations between community similarity and descriptors

Mantel correlations^31^ were estimated for species dissimilarity and surface ocean transit times, and environmental distances. Partial Mantel tests were also used to determine the relative contribution of surface ocean transit times and environmental distance in accounting for community similarity. All Mantel tests were performed using the vegan package in R^78^. Further, Multiple Regressions on Distance Matrices (MRM) were used to apportion the variability in species composition among the different predictor factors. The Mantel tests should be restricted to questions that concern dissimilarity matrices, and not ‘raw data tables’ of spatial coordinates, from which one can compute dissimilarity matrices^79^. In our study, the surface ocean transit times among sites are not vectors of raw data tables from which a dissimilarity matrix can be calculated; therefore, Mantel tests are suitable for our purpose.

### Halving-time and time-decay slope

When β-diversity correlated significantly with oceanic transit time, after partialling out by environmental factors, we estimated rates of community dispersal and species spatial turnover using two connectivity descriptors:

The time-decay slope^14,16^, which is a proxy for species turnover rates. Time-decay rates were estimated using a Type 1 linear regression equation describing the relationship between log community similarity (*S*) and log linear time (*T*):

2 $$\log \;{S} = a + b\log {T},$$

where *a* is the intercept and *b* is the slope of the time-decay relationship which reflects the rate of species turnover per unit time^14^.

The halving-time metric, which is a time-decay-based proxy for the scale of dispersal^16^. The halving-time identifies the time at which community similarity halves, and provides relevant information regarding the spatial scale of community variation^16^. We calculated this metric using the surface ocean transit times (instead of the normal geographic distances). Halving-times for each community were calculated using a logarithmic decay model:

3 $$S = c\ln \left( t \right) + {\rm int},$$

where *S* is community similarity at time *t*, *c* is the rate of time-decay, and int the intercept of the model. Assuming *S* = 1 when *t* = 0; the corresponding halving-time (*t*_H_) is:

4 $$t_{\rm H} = \frac{{\mathrm{e}^{\left( {\frac{{S_0}}{2} - {\rm int}} \right)}}}{c},$$

where *S*_o_ is the initial community similarity at the lowest transit time (100 days). The value of 100 days to obtain the *S*_*o*_ was imposed after analysing the similarity-decay of each group along surface ocean transit times. Long halving-times, represented by shallow time-decay slopes, indicate slow species turnover, while short halving-times imply fast species spatial turnover. The major advantage of the halving-time over other metrics of dispersal scales is that it can be calculated for any type of regression between similarity and distance, and offers, therefore, a useful and easily comprehensible metric to compare across studies^16^. The difference between using the halving-distance/times or the distance-decay slope arises from the intercept of the relationship between species similarity and distance (Supplementary Fig. 5). The higher the species occurrences along the stations, the higher the similarity over distance; consequently, the intercept will be higher, too. Since the halving-distance depends on the intercept, this will vary accordingly (Supplementary Fig. 5). Both descriptors, the halving-time and distance-decay slope, are key to revealing patterns of planktonic community assembly embedded in distance-decay relationships^16^.

The hypothesis that dispersal scales and species spatial turnover rates decrease with body size and local abundance was tested through the correlation between (1) halving-times and time-decay slopes and the average body size of each biological group, and (2) halving-time and time-decay slopes and the local abundance of each biological group. The correlations were calculated using parametric linear models and non-parametric bootstrap cross-validation techniques.

### Spatial patterns of β–diversity

Network graphs were used to explore spatial patterns of community similarity among all pair sites, using the igraph package^80^ in R. Specifically, sampling stations were grouped according to their species composition (based on Jaccard distances), using hierarchical clustering. In addition, the Analysis of Similarities (ANOSIM), performed using the vegan package^78^ in R, permits us to obtain a significant number of clusters for the biological group. Subsequently, network graphs were drawn with nodes (sampling stations) proportional to the similarity between sites and color-coded to represent cluster membership. In other words, the size of stations indicates the number of connections—that is, larger-sized circles share more species (or OTUs) within all stations, compared to small-sized circles, and the color represents a given hierarchical cluster. A minimum similarity threshold was imposed allowing all nodes to have a given connectivity degree.

### Code availability

All code was written in the R programming language, which is open source and freely available. Enquiries about the code used here can be directed to the corresponding author, E.V.

### Data availability

The presence/absence data of species and OTUs that support the findings of this study are publicly available in the Pangaea open repository (https://www.pangaea.de/) with the 10.1594/PANGAEA.874689 DOI identifier.

## Electronic supplementary material

Supplementary Information

Peer Review File
