# Supplementary material for: Large-scale ocean connectivity and planktonic body size
Source: Nat Commun. 2018 Jan 10;9:142. doi: 10.1038/s41467-017-02535-8 (PMC5762663; doi:10.1038/s41467-017-02535-8)
Supplement: Supplementary file 1 — Supplementary Information [file 41467_2017_2535_MOESM1_ESM.pdf]

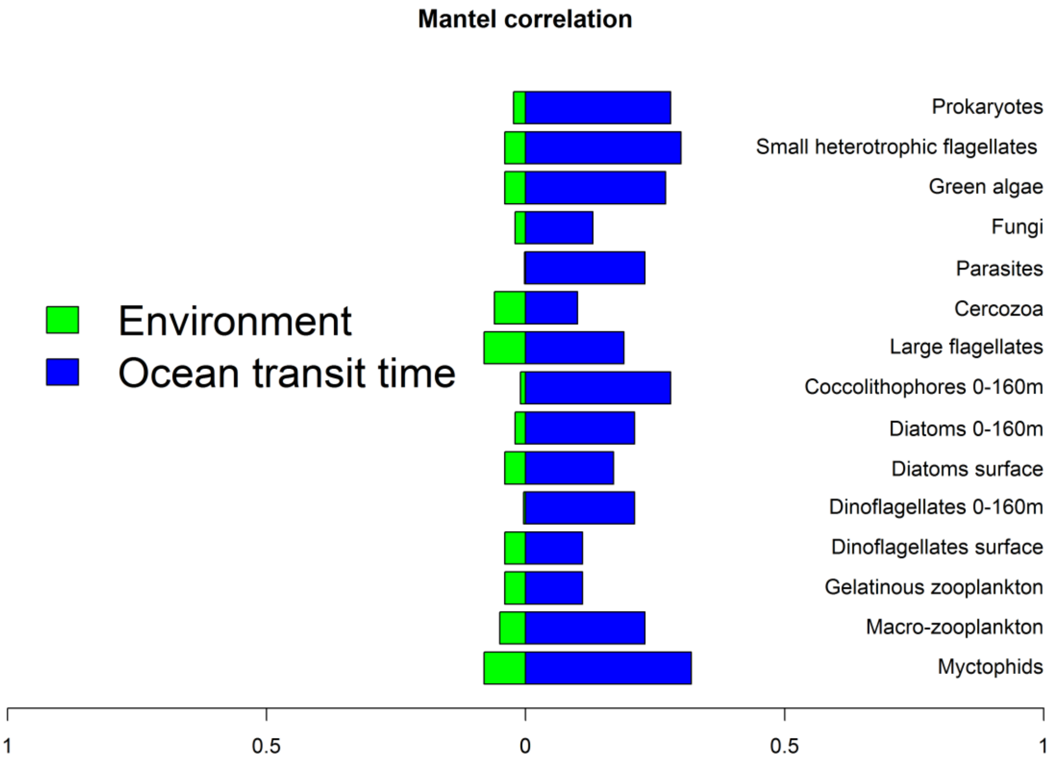

Supplementary Figure 1. Correlations between community similarity with surface ocean transit time and environment. Mantel R correlation showing the relative contribution of ocean transit time and environment shaping the community structure for the different biological groups. The Mantel tests is based on Pearson's product moment correlation using 9999 permutations. Blue: surface ocean transit time; Green: environmental distance.

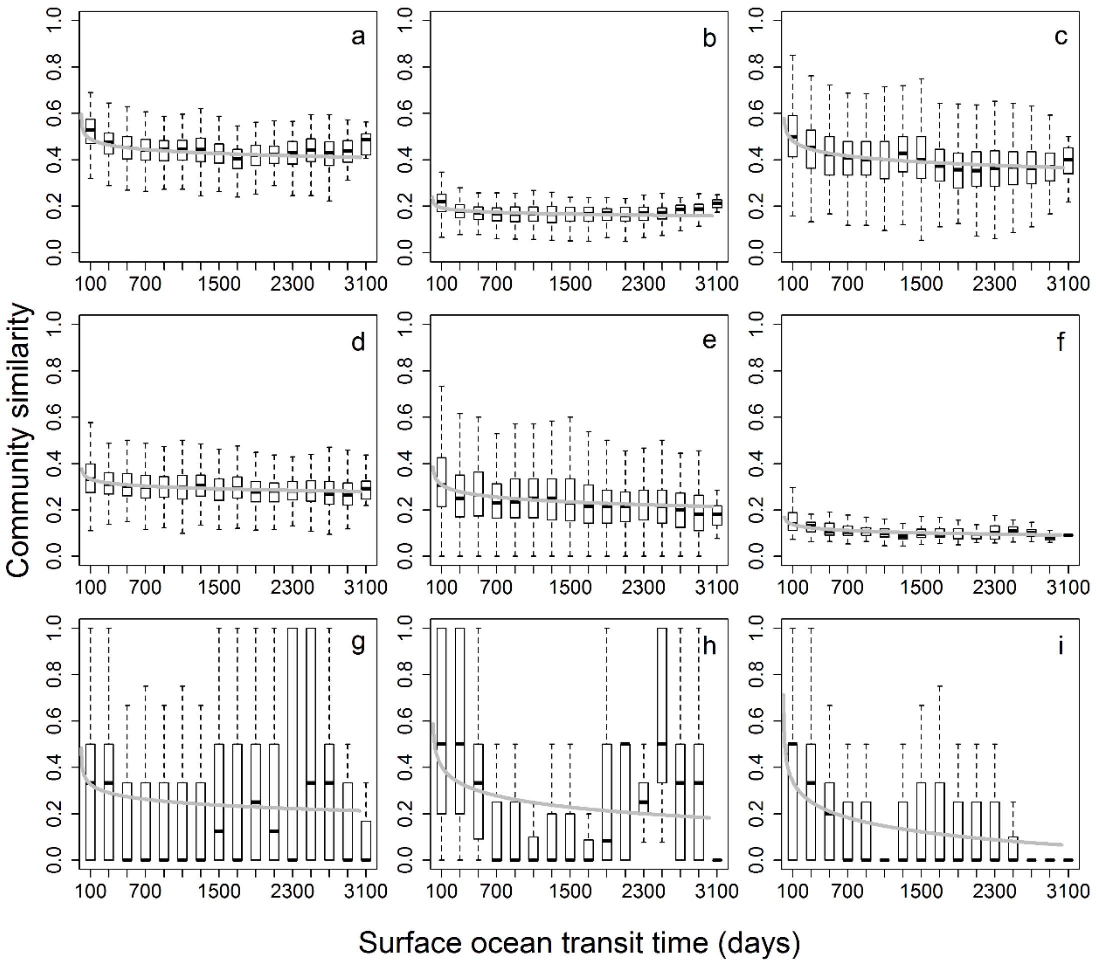

Supplementary Figure 2. Time-decay between community similarity and surface ocean transit time. Box-plots of community similarity and surface ocean transit time (bins of 200 days) for the different biological groups. The bottom and top of the box are the lower (Q1) and upper (Q3) quartiles and the band inside the box is the median. The whiskers extend up to 1.5 times the interquartile range (Q3-Q1) from the box. The points outside the whiskers are not drawn. The relationship is best approximated by a logarithmic model (grey line). (a) Prokaryotes, (b) Microbial eukaryotes all, (c) Coccolithophores 0-160m, (d) Dinoflagellates 0-160m, (e) Diatoms 0-160m, (f) Meso-zooplankton 0-200m, (g) Gelatinous zooplankton, (h) Macro-zooplankton and (i) Myctophids.

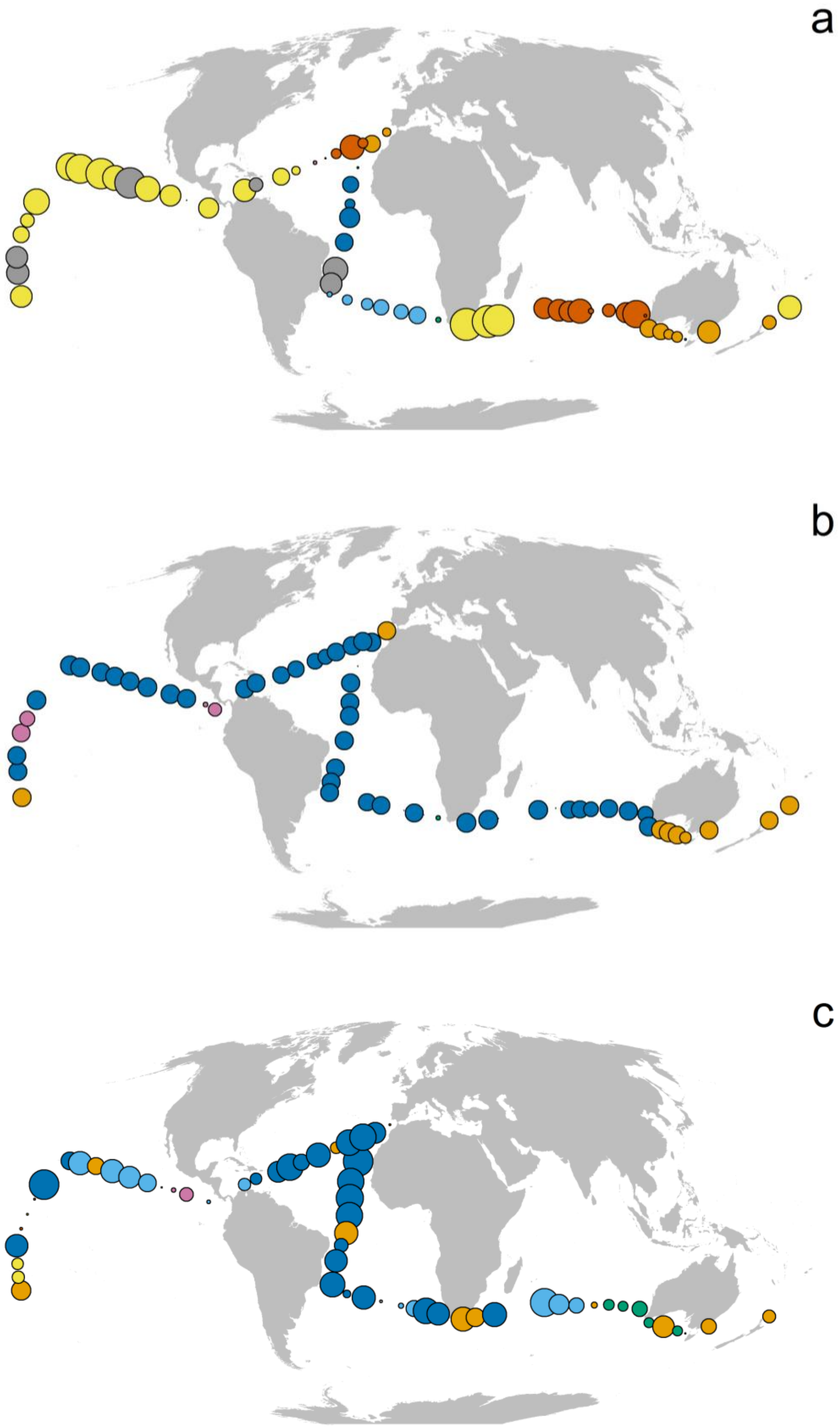
a
b
c

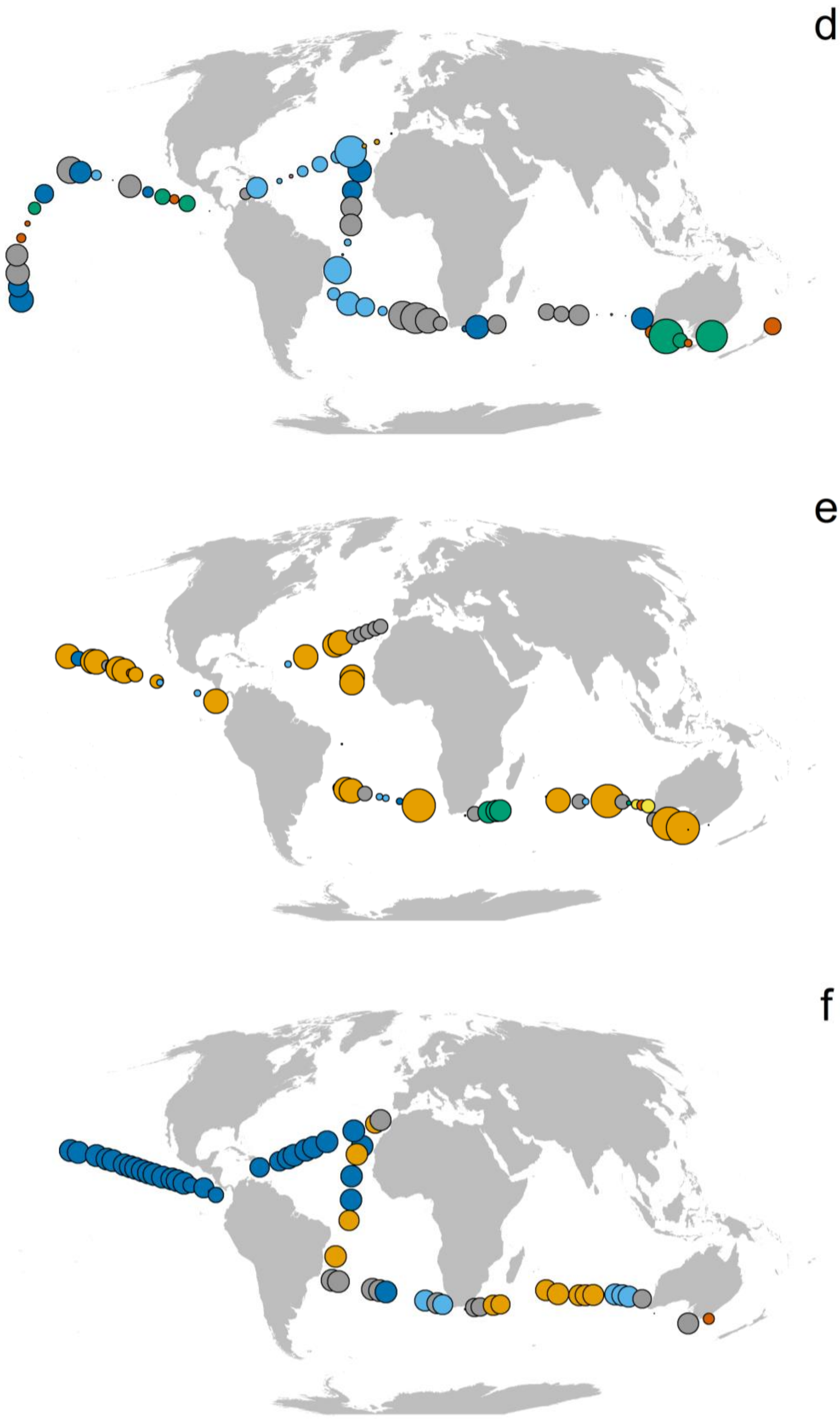

Supplementary Figure 3. Spatial community patterns. Hierarchical clustering based on the Jaccard similarity index for (a) Prokaryotes, (b) Microbial eukaryotes all, (c) Coccolithophores 0-160 m, (d) Dinoflagellates 0-160 m, (e) Gelatinous zooplankton and (f) Macro-zooplankton. Each color represents a different hierarchical cluster. The size of the stations indicates the number of connections (i.e. species or OTUs similarity between sites), that is, larger sized circles share more species (or OTUs) within all stations, compared to small sized circles. Some stations have been aggregated based on proximity for clarity.

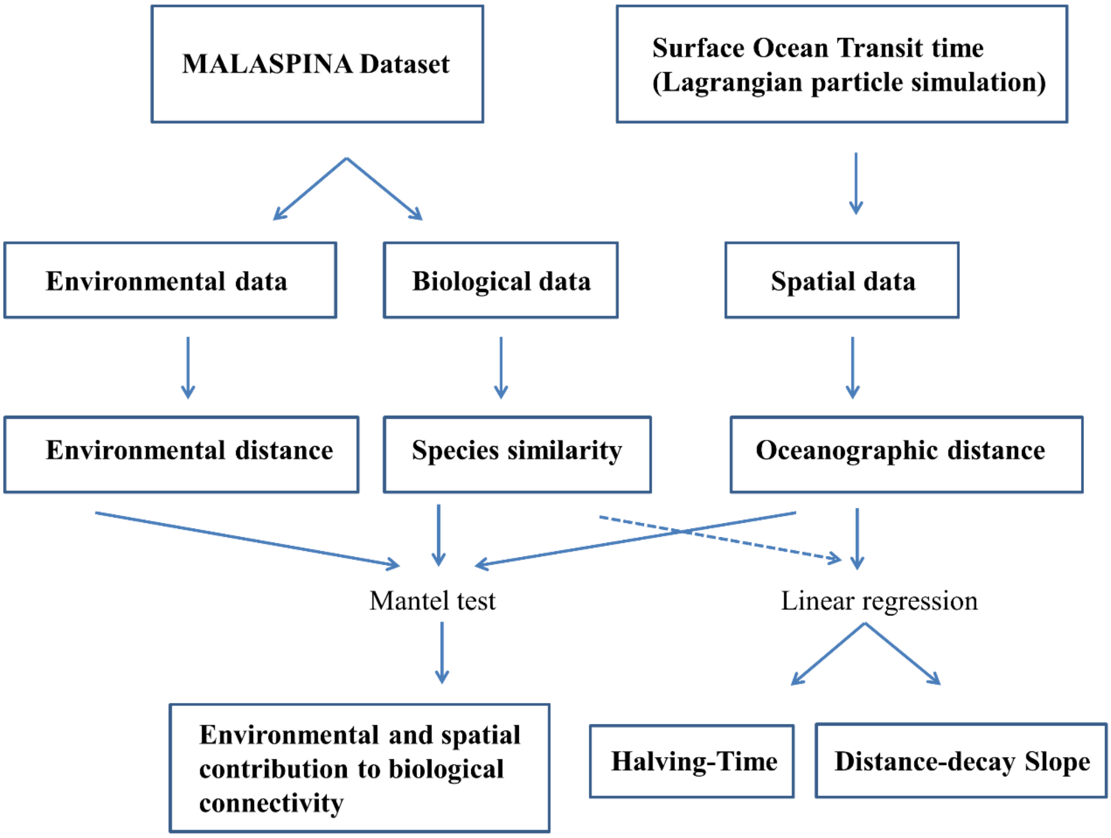

Supplementary Figure 4. Flow diagram of the used methodology. Main diagram of the methodology used to estimate the biological connectivity, in terms of dispersal scales (halving-time) and species spatial turnover (distance-decay).

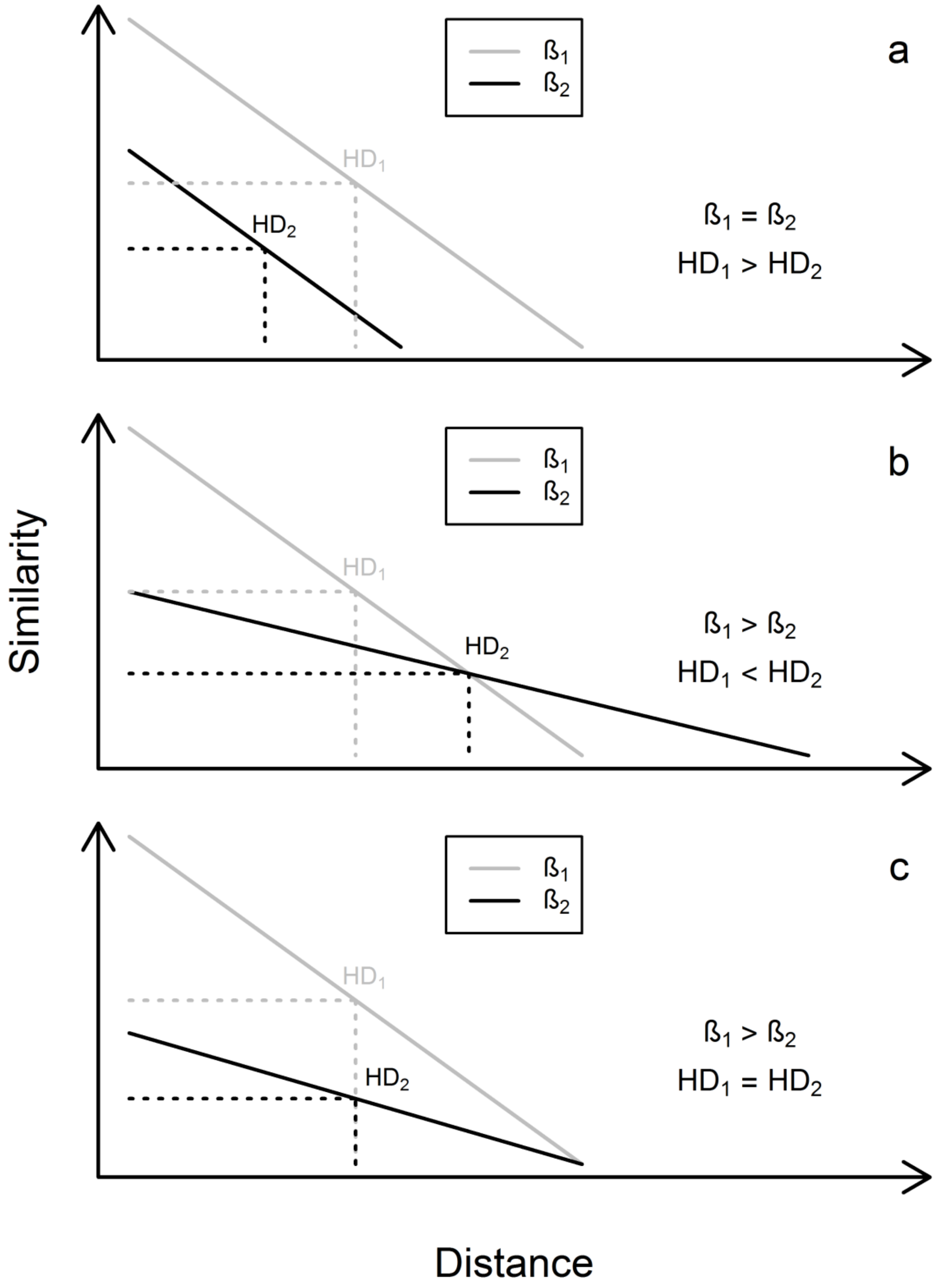

Supplementary Figure 5. Similarity decay with distance in three different cases. Conceptual figure denoting the similarity decay with distance in three different cases: (a) two equal distance-decay slopes ($\beta_1 = \beta_2$) but different halving-distance ($HD_1 > HD_2$), (b) two different distance-decay slopes ($\beta_1 \neq \beta_2$) but different halving-distance ($HD_1 > HD_2$) and (c) two different distance-decay slopes ($\beta_1 \neq \beta_2$) but equal halving-distance ($HD_1 = HD_2$).

Supplementary Table 1. Correlation between environment and currents. Mantel correlation between environmental distance and ocean transit times considering all-pairs of sampling sites. The statistical significance of the comparison is assessed using Mantel tests based on Pearson's product moment correlation using 9999 permutations.

Factors	Number of pairs	Mantel R correlation	p-value
Surface ocean transit time vs environmental distance	133	0.09	0.001

Supplementary Table 2. Correlation between body size and environment. Evaluation of the log-log relationship between the logarithms of environmental distance and group size, showing parametric models (all observations included) and non-parametric bootstrap cross-validations (95% confidence interval). Ln: Napierian logarithm.

All biological groups	Statistic	Parametric model	Bootstrap
Ln (environmental distance) vs Ln (Size)	Confidence interval		(-0.054, 0.683)
	p-value	0.27	>0.05
	RMSE	1.05	
	Adjusted r^2	0.019	
	Equation	y=-3.322+0.097x	

Supplementary Table 3. Description of the biological groups and identification and quantification methods for abundance. Groups analyzed in the Malaspina survey, with its description, identification techniques and the methods used for global abundance estimation.

Main biological group size range (mm)	All biological groups	Group description	Taxonomical or OTU identification	Methods used for abundance estimation
Prokaryotes (0.0003 - 0.001)	Prokaryotes	Archaea and Bacteria	HTS (High Throughput Sequencing)16SrRNA	Flow cytometer counting
Microbial eukaryotes all (0.0008-0.003)	Microbial eukaryotes all	All picoeukaryotes (cells 0.0008-0.003 mm), including small heterotrophic flagellates, green algae, fungi, parasites, cercozoa, large flagellates, diatoms and dinoflagellates, and other minor components	HTS 18SrRNA	Microscope epifluorescense counting
Microbial eukaryotes sub-groups	Small heterotrohic flagellates	Small heterotrophic flagellates (MAST lineages – Marine Stramenopiles)	HTS 18SrRNA	Not determined
	Green algae	Green algae (Mamiellophyceae and Prasinophyceae)	HTS 18SrRNA	Not determined
	Fungi	Fungi (Ascomycota, Basidiomycota and basal lineages)	HTS 18SrRNA	Not determined
	Parasites	Parasites (MALV lineages – marine Alveolates)	HTS 18SrRNA	Not determined
	Cercozoa	Cercozoa (all Cercozoa excluding the chlorarachniophytes)	HTS 18SrRNA	Not determined
	Large flagellates	Large flagellates (Katablepharidae, Picozoa and Telonema)	HTS 18SrRNA	Not determined
	Dinoflagellates surface	Dinoflagellates at surface	HTS 18SrRNA	Not determined
	Diatoms surface	Diatoms at surface	HTS 18SrRNA	Not determined
Coccolithophores 0-160m (0.002-0.5)	Coccolithophores 0-160m	Coccolithophores, depth-integrated abundance.	Traditional taxonomy, Inverted microscopy-visual examination	Inverted microscopy counting
Dinoflagellates 0-160m (0.002-0.5)	Dinoflagellates 0-160m	Dinoflagellates, depth-integrated abundance.	Traditional taxonomy, Inverted microscopy-visual examination	Inverted microscopy counting
Diatoms 0-160m (0.002-0.4)	Diatoms 0-160m	Diatoms, depth-integrated abundance.	Traditional taxonomy, Inverted microscopy-visual examination	Inverted microscopy counting
Meso-zooplankton 0-200m (0.3-5)	Meso-zooplankton 0-200	Annelida, Echinodermata, Ctenophora, Tunicata, Nemertea, Cnidaria and others	18SrRNA	Not determined
Meso-zooplankton surface (0.3-5)	Meso-zooplankton surface	Annelida, Echinodermata, Ctenophora, Cnidaria, Mollusca, Chordata, Arthropoda	Morphological characters, visual examination	Traditional taxonomy
Gelatinous zooplankton (>5)	Gelatinous zooplankton	Salpìdae, Cnidaria, Ctenophora	Morphological characters and high-resolution photography	Traditional taxonomy
Macro-zooplankton (4-15)	Macro-zooplankton	Nudibranquia, macro-crustaceans (phyllosoma and megalopa decapoda larvae) and Halobates insects	16SrRNA and Cox1 gene	Estereo-microscope counting
Myctophids (20-110)	Myctophids	Myctophid fishes	Morphological and morphometric characters	Traditional taxonomy

Supplementary Table 4. Description of the biological dataset. The biological groups members with the number of species and OTU (Operational Taxonomic Unit) and its habitat. E = Epipelagic, N = Neustonic, M = Mesopelagic.

Main biological groups	Number of species / OTU	Number of stations	Habitat
Prokaryotes	1218	120	E
Microbial eukaryotes all	35615	112	E
Coccolithophores 0-160m	47	133	E
Diatoms 0-160m	68	133	E
Dinoflagellates 0-160m	236	133	E
Meso-zooplankton 0-200m	4283	36	E
Gelatinous zooplankton	12	61	N
Macro-zooplankton	46	65	N
Myctophids	12	95	M&N
**Microbial eukaryotes biological sub-groups**	**Number of species / OTU**	**Number of stations**	**Habitat**
Small heterotrophic flagellates	1014	112	E
Green algae	451	112	E
Fungi	59	89	E
Parasites	20466	112	E
Cercozoa	84	107	E
Large flagellates	375	112	E
Dinoflagellates surface	8391	112	E
Diatoms surface	85	93	E
