# Supplementary material for: Large-scale ocean connectivity and planktonic body size
Source: Nat Commun. 2018 Jan 10;9:142. doi: 10.1038/s41467-017-02535-8 (PMC5762663; doi:10.1038/s41467-017-02535-8)
Supplement: Supplementary file 2 — Peer Review File [file 41467_2017_2535_MOESM2_ESM.pdf]

Reviewers' comments:

Reviewer #1 (Remarks to the Author):

The manuscript describes size-dependent distance decay relationships among groups of planktonic organisms. The main findings, based on observational data, are that (1) overall connectivity, not local selection, predicts beta-diversity along transects sampled in the (sub)tropical oceans and that (2) there is a general size-dependence, with larger organisms (<=10mm) exhibiting steeper distance decay relationships, while especially protists revealed rather flat relationships. The analysis is mostly convincing, though there are a number of technical issues that need to be addressed. In particular, the underlying mechanism (size itself or other factors involving allometric scaling) need to be worked out more clearly.

(1) By comparing the empirical data from various biological groups with plastic particles as neutral marker, it is concluded that physical connectivity actually should be rather independent from size, i.e. similar for all (passively dispersing) groups (l. 208 ff). So it is concluded that differences in generation time and population size (which correlated with body size following general allometric scaling rules) may rather drive the differences seen among groups, not body size itself. This is a very relevant aspect, but treated inadequately in the paper. IF it is suggested that generation time, not size, drives the relationships, this should be tested, and decay rates should (also) be plotted against generation time. Though size and community turnover are related, there should be considerable variation in this allometric scaling to check which of the two predictors matters. Notably, in phytoplankton, it is not the smallest organisms that exhibit fastest reproduction, but cells of intermediate size (Maranon, Annu. Rev. Mar. Sci. 2015. 7:241–64). This discrepancy in the general allometric scaling could be helpful to discern whether indeed the smallest (picoplankton), or the fastest growing cells exhibit flatter distance-decay pattern. Another relevant aspect not discussed is the role of sinking losses, which again scale with body size. Sinking losses should be minimal for the smallest particles, allowing their populations to travel farthest.
(2) From finding overall flatter distance decay relationships in protists, it is concluded that they are less dispersal limited (in agreement with numerous statements made by previous studies). However, shouldn't we then expect a stronger local sorting for those groups that are less dispersal limited (as their close-to ubiquitous distribution should foster local selection)? Fig. S2 doesn't confirm this.
(3) The statements made on the relationship between productivity and phytoplankton diversity (l. 241-245) are confusing. As it stands, it is suggested there is a simple relationship, which is exactly not the case (e.g. Vallina et al. 2014 Nature Communications 5, Article number: 4299 (2014)). The authors refer to positive *edge effects* at fronts – not a direct relationship between productivity and diversity.
(4) Reg Fig 3, cluster analysis – I found these Figures very hard to understand. The colors are apparently indicating clusters, same color=same cluster=connected – but why then there are circles with same color but different size in one plot?
The Figure would also be easier to follow if you add one figure illustrating hydrological connectivity. In the text you refer to fronts & gyres (and Hawaii as barrier), most readers (inc me) aren't that familiar to superimpose them onto the map. A figure on connectivity could simply be based on the sample locations, with symbols reflecting the connectivity to adjacent sampling sites.

Specific comments
l. 159 – 'no relationship between size and the scale of dispersal in micro plastic' – as I understood, no size categories were differentiated in plastic (Fig 2), so how would you detect such a relationship? I
in this context – how did you measure beta-diversity in plastic particles?

l. 246 – enter 'located' in sentence: "sample sites *located* between subtropical gyres"

Fig.1 – The color code is very useless with >10 lines – you can improve legibility by adding labels at the end of each line rather having a separate legend, or at least having the items of the legend ordered according to the sequence of the lines. I could not align legend to lines, due to too similar colors.

Fig. 1 - why two groups are estimated for larger scale on x-axis (longer lines)?

Reviewer #2 (Remarks to the Author):

I really appreciate what this research group has tried to do, it is a very interesting problem. Their goal was to sample planktonic diversity - from prokaryotes to small fishes, with attendant differences in mobility and behavior - using a massive (but still linear) transect of global oceans. From these samples, they would identify the taxon using traditional and molecular methods, calculate the diversity at a given location, and contrast that diversity with every other station in the transect to estimate beta diversity. By asking how distinct any two stations are, they were then able to correlate this result with environmental heterogeneity between pairs of sites as well as oceanic transit times between any pair of sites. This is a massive undertaking; the results are intriguing, but I'll return to that later.

I'll first note that the paper is poorly edited, with Figures jumbled in order and often poorly referred to. Overall, however, the writing is good and the introduction sets the problem very well in a short space. What I immediately was intrigued by was how the diversity would be catalogued at each site, as well as how it would be contrasted.

The Methods section is poorly organized and very confusing. Note first that Figure 1 (line 266) does not refer to the Malaspina Expedition. I believe you are referring to Figure 3 (lines 745-748)? The sampling of diversity I assume is done appropriately to avoid bias, and was done homogeneously throughout the expedition, so I leave consideration of sampling methods for another reviewer (though depth of the neuston sampler could be useful). My concerns lie with the scant details provided for identification of recovered diversity. Traditional methods (visual inspection) was used to identify phytoplankton (surely this is only to a higher taxonomic level, but little additional information is provided), the gelatinous zooplankton (surely a group harboring considerable cryptic diversity!), and lantern fishes; this last group I will hope can be adequately validated by traditional methods. However then, the macrozooplankton were identified with "partial sequences of 16S rDNA and CoxI genes". How? Sanger, or next-generation? How many individuals per site? What were the criteria for species identification, for OTU separation? What was the consideration for cryptic diversity that may not have been previously described and available in genomic databases? None of this information is provided. What quality assessment of data, etc. etc. The same criticism could be applied to the mesozooplankton which were identified using 18S data instead, and thus almost surely lumping together cryptic diversity that cannot be distinguished with such a slow-evolving locus. No information is given on how, methodologically, the 16S rDNA data was collected or analyzed for microbial prokaryotes - was this Sanger? Was it metagenetics, and analysis with Qiime or similar packages? What were the criteria for separation, again? How can these data be referred to as "(unpublished sequences)"? That is not really appropriate for a study like this, any new published study must include some mechanism by which the data will become available; perhaps this is just a placeholder during review. Finally - note I am still on lines 294-304 alone - the discussion of OTU is of course exactly what is important in this study, so much more consideration and explanation is necessary.

OTUs and species are described in the subsequent paragraphs. The analyses then follow starting at line 319. First please note the equations in your manuscript are barely readable in the PDF provided. I'm also concerned that equation (1), for Jaccard dissimilarity, does not match the information I have for this index (Jost et al Chapter 6 in Magurran & McGill 2011. This same

resource notes that in a study like this, multiple-site versions of this statistic should be applied because of the non-independence of multiple data points; it also suggests that the Jaccard index is less appropriate than the multiple-site version of the Sørensen index, but my main concern is the wrong version being applied. Following this, the authors point to other resources for their environmental matrix and their surface ocean transit time matrix. The latter in particular I consider very important; though my expertise is not in physical modeling of the ocean, I am concerned that what is being used in this paper is a very local-scale Lagrangian model in a heavily studied region of the world for these dynamics (the Southern California Bight). Whether similar methods apply to the global models, at larger scales, in particular when applied to a linear transect for which different points on the transect are likely to have non-linear and multiple paths of connectivity, is quite concerning that so little explanation is given for how one method has been scaled up from an area of about 10,000km^2 to global scales.

The organization remains a problem in the rest of the methods; "distance-decay slopes" are explained 2 sections after the section called "Halving-distance and distance-decay slope" (starting on line 354). I will say that I do like the idea of using plastic particles as a null approach, but even this (separating into 16 colors as "species") brings me some concern later when looking at results. The Mantel and linear regression approaches are fine with me.

Back to Results! (Line 119). I assume on Line 123 you mean "significantly negatively correlated". These results are intriguing when stated, but the effect sizes are hard to interpret. The "halving times" for microbial diversity are on the order of 10^6 days, or thousands of years, perhaps longer than current estimates of the total turnover time of any point in the surface ocean? Here again, references to Tables (line 149) are incorrect. Even if the halving time for macrozooplankton is 207 days (an interesting and more relevant figure). There may indeed be a size factor involved in these distributions (not shown in "Table 5" referred to in line 159); it is not clear how the plastics data inform this analysis, as there is no relationship between size and scale of dispersal in plastics, but those "OTUs" are artificially defined by color, which has nothing to do with environmental context or point of origin. In fact, it isn't clear to me why the slope for plastics isn't zero?

Given the concerns raised above, I left the Discussion alone. I think this is an astounding data set (though I don't know most of the details), and a very interesting question. In many ways, the methods seem appropriate; however, in many other ways the degree of hand-waving and incautious consideration of the complexity of this problem (what is diversity? how is it distributed? how do environmental forcing mechanisms distinct from ocean currents interact with those ocean currents to drive these distributions?) makes me concerned that we don't yet know the answer from these data.

Reviewer #3 (Remarks to the Author):

The research question is interesting and this represents a significant global scale study likely of interest to a wide range of readers. However, my main issue when reviewing this manuscript was that structural issues and the use of interchangeable terminology meant that I had to read the manuscript a few times before I fully understood your definitions and methods. Secondly, some detail is lacking from the methodology, meaning that it has been difficult for me to comment on the appropriateness of your scientific methods.
I therefore think the manuscript needs improving in two ways:
A) Restructuring and clarification for a general scientific audience.
B) Additional methodological detail.
I hope the comments below will help you to clarify the manuscript.
Define and be consistent with your use of the terms 'dispersal', 'connectivity' and 'ocean transit times', as these are often used in slightly different contexts in other publications and could be confused for one another in this manuscript. I think of 'dispersal' as the transport of an individual by ocean currents (what you call connectivity), whereas what I think you mean by dispersal scales

here is more akin to species range (i.e. colonisation of increasingly wider area over multiple generations, until equilibrium is reached). This distinction between biogeography versus short-term processes is an important one.
Your use of the term connectivity is also not clear. For example, in lines 182-183 you state that connectivity is determined by both transport by ocean currents (what I think of as dispersal) plus environmental filtering. I would say this was the correct definition of connectivity However, you then state that "ocean connectivity (through our estimates of surface ocean transit times) explains a larger fraction of the variability in… community similarity, relative to environmental factors" (lines 184-187).
I would therefore recommend clearly defining and separating the two metrics; a) scales of dispersal (determined empirically based on species composition, i.e. the distance-decay slopes) and b) timescales of surface ocean connectivity/ocean transit times (derived from a previous modelling study), and how they are derived early on in the manuscript. Make it clear throughout the manuscript that only "surface ocean transit time/connectivity" is modelled. On first reading the manuscript I mistakenly assumed that you had determined the dispersal scales of the different size plankton groups using the Lagrangian particle simulation approach. You don't state that you are determining distance decay slopes until line 107, and then fail to link these to the "dispersal scales" discussed in the abstract.
With this is mind, I think the structure of the introduction could be improved to make your aims and the hypotheses you are testing clearer to the reader. As I understand it, your main aim was to test whether scales of dispersal vary amongst different size groups. You do this by 1) firstly testing how much of the variation in β-diversity can be explained by oceanographic distance (modelled surface ocean transport times) over changes in environmental variables (despite the fact that, as you state at lines 91-94, the two are often correlated). Given that this tells you that β-diversity is indeed predominantly controlled by oceanographic distance, you then 2) reverse the process - using patterns of β-diversity to estimate how far different size classes of organisms are able to disperse (given the caveat that a proportion of the limitation to dispersal will still be environmentally driven). This took me a while to get at, and was not clear from the introduction alone. I think the two strands of the study should be more clearly separated.
Regarding detail on the methodology, you state that for the surface ocean transit time matrix (lines 345-352) you "[used] the approach of Watson et al. [2011]", but do not give any specifics of the model configuration for this study (Figure 5, presumably referring to S5, does not give any detail). I therefore cannot comment on this approach here. For example, what spatial and temporal scales did you model particle transport over? What depths? I think it would improve the interpretation of your results if you explicitly stated how you derived the ocean transport times - for example that you did not consider behaviour (e.g. swimming) or depth - and how this may have affected your correlation between ocean transport times (your 'connectivity') and β-diversity? For example, you state that body size is not the sole driver of 'dispersal', discussing also population densities (population size at line 101) and generation times (lines 205-214) - could differing depth distributions between groups explain some of your missing variation in community structure (i.e. differing transport due to changes in currents with depth)? (In my earlier misunderstanding of your approach, on reading lines 62-64 of the abstract, in which you state "larger-bodied plankton… in near-surface epipelagic waters have significantly shorter dispersal scales", I found myself wondering whether the different dispersal scales were indeed due to differing body size, or alternatively due to their depth distribution).
It is not clear to me how you defined microplastic community composition/distance-decay relationships to compare with the various plankton groups.
Minor comments:
Define specialist terms in the main text: β-diversity (line 81), halving time (line 142), spatial turnover rates (line 142).
Line 60/65: A pet hate of mine maybe, but I recommend avoiding terminology such as 'reveal' and 'confirm', replacing it with something like 'suggest' or 'provide evidence that'. Also "of course" on line 173.
Lines 99-105: The rationale for the argument that smaller organisms are likely to disperse further is not clear to me from this paragraph. Could you perhaps expand/simplify this hypothesis, using

the references you cite?
Line 150: Large-bodied groups?
Some typos: Line 84: “by as a”? Line 92: “weather”. Line 98: “,”. Line 352: “groups”.

Response to the reviewer's comments

Manuscript No.: NCOMMS-16-24484 entitled "***Large scale ocean connectivity and planktonic body size***"

- RC: Reviewer comment (in italic and grey)
- Author Response (AR) in blue

Reviewer #1

- **RC 1.1:**

"By comparing the empirical data from various biological groups with plastic particles as neutral marker, it is concluded that physical connectivity actually should be rather independent from size, i.e. similar for all (passively dispersing) groups (l. 208 ff). So it is concluded that differences in generation time and population size (which correlated with body size following general allometric scaling rules) may rather drive the differences seen among groups, not body size itself. This is a very relevant aspect, but treated inadequately in the paper. IF it is suggested that generation time, not size, drives the relationships, this should be tested, and decay rates should (also) be plotted against generation time. Though size and community turnover are related, there should be considerable variation in this allometric scaling to check which of the two predictors matters.

AR: We thank the reviewer for this insightful comment. We have excluded the micro-plastics data from the analysis, according to the comments and concerns raised by the three reviewers (see response to reviewers 1.7, 2.7 and 3.6). Additionally, we have included new analyses concerning the relation between local abundance (i.e. density) and dispersal scales (see below) and we have included additional explanations on the relationship between body size and abundance, within the framework of the metabolic theory of ecology[1] (see lines 105-108). "Body size is the dominant factor determining individual metabolic rates[2] and, according to the Metabolic Theory of Ecology[1], it also controls numerous ecological processes. For example, smaller organisms have lower metabolic rates, faster growth rates, shorter generation times and higher energy needs relative to larger organism". As generation time is also related to body size, this is equally valid for generation times[1,3-5]. However, as relatively accurate generation times are more difficult to obtain for some of the groups, we have focused the analysis on abundance, a parameter measured during the cruise, and included the relation between generation times and body size in the discussion: (see lines 108-110) "Other implications of body size are that small organisms are generally more abundant than larger organisms[2]". Following the reviewer's suggestion, we have compiled the abundance data from the Malaspina survey (Table 2A). We have analyzed the abundance vs. dispersal scale (Halving time and time-decay slope) relationship for each group, and we show that there is a significant, yet slightly stronger correlation relative to that to of size vs. dispersal scale (see Figure 3 and table 3). For that reason,, we have changed the text including abundance data in the results (lines 168-172) "these groups of small organisms showed the highest local abundance values (prokaryotes = $3.30\times10^{11} \pm 4.10\times10^{10}$ ind.m^{-3}; microbial eukaryotes = $1.72\times10^{9} \pm 1.49\times10^{9}$ ind.m-3), being 8-10 orders of magnitude more abundant that larger organisms (macro-zooplankton =

$1.79 \times 10^{-1} \pm 2.5 \times 10^{-1}$ ind.m^{-3}; myctophids = $3.5 \times 10^{-3} \pm 1.9 \times 10^{-2}$ ind.m^{-3}) (Table 2B)" and the correlation (abundance vs size and time-decay slope) related text (lines 175-180) "As expected, we also found a strong significant negative correlation between the organism body size and its abundance ($r2=0.93$; p-value < 0.001) (Fig. 3C) and a significant positive log-log relationship between the local abundance of the biological groups and halving-time and time-decay-slope (Fig. 3A and Fig. 3B, Table 3A)". We have also pointed out that the mechanism underlying the dispersal scale is not the size *per se*, but also the abundance, which is tightly linked with body size[6] (see lines 238-247) "In order to explain the underlying process of this empirical finding, we have identified a significant positive relationship between the local abundance and the community dispersal scales. This was expected since local abundance scales negatively with body size[1,4], as confirmed in our data. Moreover, generation time also scales negatively with body size[1,4]. Therefore, we suggest that large population densities and short generation times of micro-planktonic organisms are the mechanisms explaining the larger geographic range and relatively weak spatial structure of these organisms[7-10]. In contrast, larger planktonic organisms have in general longer generation times and smaller population densities[11], and therefore they are more sensitive to local extinctions and ecological drift, resulting in stronger spatial structure". The dispersal scale vs. size relationship could be "counterintuitive", because one might expect higher dispersal scales in larger body-sized taxa. In that vein, we have included new text comparing the dispersal capacities of actively and passively dispersed taxa, and compared also with terrestrial groups (see lines 277-285) "Our data support existing understanding that β-diversity in the pelagic domain increases with body size in small and mainly passive organisms while decreases in actively mobile larger taxa (pelagic fishes, cetaceans), because high dispersal capacity reduces compositional differences between sites[12]. Furthermore, considering that the community dispersal scale defined here is a good proxy of the geographic range of a particular community, it seems that the local abundance of the species from an ecological guild relates positively to their geographic range in plankton, similar to many other groups from marine and terrestrial domains, including both passively and actively dispersing species[13]".

- **RC 1.2:**

Notably, in phytoplankton, it is not the smallest organisms that exhibit fastest reproduction, but cells of intermediate size (Maranon, Annu. Rev. Mar. Sci. 2015. 7:241–64). This discrepancy in the general allometric scaling could be helpful to discern whether indeed the smallest (picoplankton), or the fastest growing cells exhibit flatter distance-decay pattern.

AR: We agree with the reviewer in that biomass specific production and growth rates in phytoplankton peak at intermediate cell sizes[14], but division rates are highest in smallest cell sizes[15], and, in our opinion, this is what matters in terms of dispersal. Moreover, the size range we analyzed here spans from prokaryotes to small fishes, not only phytoplankton, and hence, for this large range, size and growth rates are tightly linked[16,17].

- **RC 1.3:**

Another relevant aspect not discussed is the role of sinking losses, which again scale with body size. Sinking losses should be minimal for the smallest particles, allowing their populations to travel farthest."

AR: We appreciate the point raised by the reviewer. It is true that the sinking rates are likely smaller in bacteria compared to, for example phytoplankton, because sinking rates depend on the cell size[18,19], hence allowing small organism to disperse further. However, large-sized organisms, for example zooplankton, have some capacity to control their position in the water column and compensate the rates of sinking by performing diel vertical migrations. We have included the following sentence in the text hoping to cover what the reviewer points out (lines 247-250) "In addition, sinking losses for small passively-dispersed plankton (from prokaryotes to phytoplankton), which also scale with body size[19], are lower for the smaller organisms, allowing their populations to travel farthest compared to larger ones".

- **RC 1.4:**

"From finding overall flatter distance decay relationships in protists, it is concluded that they are less dispersal limited (in agreement with numerous statements made by previous studies). However, shouldn't we then expect a stronger local sorting for those groups that are less dispersal limited (as their close-to ubiquitous distribution should foster local selection)? Fig. S2 doesn't confirm this"

AR: Whether ubiquitous species are more constrained by local sorting is an open question. Particularly in protists, which are small-sized organisms that show pan-dispersed distributions, the flat distance decay relationships found in this study means that they are less limited by climate, as can be seen in supplementary Figure 1, because they tolerate large environmental gradients. We have included the following text in order to address the point raised by the reviewer: 1) Results (lines 147-149) "we found no pattern in the relation between the relative contribution of environmental drivers and body size (non-parametric bootstrap p-value = 0.55, details not shown)"; 2) Discussion (lines 252-257) "The relative contribution of environmental drivers was not correlated with body size in plankton and micro-nekton, in contrast to the increasing importance of habitat filtering with organism body size suggested by Farjalla 2012[20]. However, Farjalla 2012[20] studied the community structure of only three groups (bacteria, zooplankton and macro-invertebrates) within reduced spatial scales compared to the global scales addressed here, as their study focused in the coastal zone of Southern Brazil".

- **RC 1.5:**

*"The statements made on the relationship between productivity and phytoplankton diversity (l. 241-245) are confusing. As it stands, it is suggested there is a simple relationship, which is exactly not the case (e.g. Vallina et al. 2014 Nature Communications 5, Article number: 4299 (2014)). The authors refer to positive *edge effects* at fronts – not a direct relationship between productivity and diversity."*

AR: We agree with the reviewer that the statement about the relationship between phytoplankton productivity and diversity might be confusing. In order to clarify this we have deleted lines 241-242 (in the old version) which referred to the relationship between diversity and production, since our paper is not focused on local diversity patterns among different planktonic taxa, but on beta-diversity.

- **RC 1.6:**

"Reg Fig 3, cluster analysis – I found these Figures very hard to understand. The colors are apparently indicating clusters, same color=same cluster=connected – but why then there are circles with same color but different size in one plot? The Figure would also be easier to follow if you add one figure illustrating hydrological connectivity. In the text you refer to fronts & gyres (and Hawaii as barrier), most readers (inc me) aren't that familiar to superimpose them onto the map. A figure on connectivity could simply be based on the sample locations, with symbols reflecting the connectivity to adjacent sampling sites."

AR: We agree with the reviewer that the legend of the figure does not help to understand the plot. Each color represents a different hierarchical cluster, and the size of the circles stand for the degree of connectivity. Stations with larger circles share more species (or OTUs "Operational Taxonomic Unit") among the whole set of stations, compared to stations with small circles, where the number of shared species, i.e. connections, are fewer. It might have happened, though, that two similar clusters, say two red color circles, to have varied sizes because despite being of the same species assemblages or cluster, the number of shared species with other stations is different. For instance, if sample A has a large number of species and the sample B has a small number of species and this is a subset sample of A, hence it is expected that both are in the same cluster and sample A would have the circle size larger than that of B. Hoping to clarify this we have included the following text in the legend of the Figure 5. "Hierarchical clustering based on the Jaccard index for A) Diatoms 0-160 m, B) Meso-zooplankton, and C) Myctophids. Each color represents a different hierarchical cluster. The size of the circles indicates the number of connections (i.e. species/OTUs similarity between sites). Communities with larger-sized circles share more species (or OTUs) with all stations, compared to those represented by small-sized circles. For clarity, some stations have been aggregated due to their geographical proximity".

In the new version of the manuscript, we have included a new figure (Figure 4) and text showing data of the timescales of the ocean connectivity model by Jonsson and Watson 2016[21]. We show i) the minimum ocean transit times, based on the Dijkstra algorithm, from a particular patch to connect all other patches in the global ocean ("time from") and ii) the minimum ocean transit time for water to go from all patches to a given patch ("time to"), using two randomly chosen Malaspina stations located off Hawaii and off South-African coast (see lines 181-191) "Minimum transit times for water to go from all global surface ocean locations to Malaspina stations off Hawaii and the South-African coast are shown in Figures 4A and C respectively. Similarly, minimum transit times from these Malaspina stations to all other global surface ocean locations are shown in Figure 4B and D. These figures are outcomes of previous work on global surface ocean connectivity[21], and reveal the spatially heterogeneous nature of ocean connectivity and dispersal. For example, minimum transit times from these Malaspina stations to nearby

surface ocean locations are short, relative to those to far-off locations. At basin scale, spatial structure is well observed, being the Atlantic Ocean a basin which is less connected than the Indian and Pacific Oceans (Fig. 4). The main conclusion of this work is that the global ocean can be connected over timescales of a decade". In the discussion, we have also linked the spatial heterogeneity in the modelled surface ocean transit times and the spatial structure of the planktonic groups (see lines (296-301) "In addition, the modelling results of global ocean transit times have shown that the Atlantic Ocean is less connected compared to the Pacific and Indian oceans. This is mirrored in the spatial clustering of planktonic organisms found in our data, particularly in myctophids and macro-zooplankton, where a set of unique clusters are only seen in the Atlantic Ocean (red color stations), and other unique clusters (pink and purple stations) only in the Pacific and Indian Oceans".

Specific comments

- **RC 1.7:**

RC: l. 159 – 'no relationship between size and the scale of dispersal in micro plastic' – as I understood, no size categories were differentiated in plastic (Fig 2), so how would you detect such a relationship? In this context – how did you measure beta-diversity in plastic particles?"

AR: According to the reviewer comment 2.7, we have excluded the micro-plastic data from the analysis. In the Malaspina survey, micro-plastics were divided in different sizes and colors, though they could be grouped as a function of the size, particularly in subtropical gyres[22]. However, as the reviewer points out, the color and the size of the plastic has no relation with the environmental context or place of origin or dispersal pattern, contrary to planktonic populations. For this reason, we agreed that it is incorrect to estimate beta-diversity and distance-decay relationships, because there is no evidence of decay (slope=0) in the plastics, and dispersal distances cannot be inferred.

- **RC 1.8:**

*"l. 246 – enter 'located' in sentence: "sample sites *located* between subtropical gyres"*

AR: Agreed. Done

- **RC 1.9:**

"Fig.1 – The color code is very useless with >10 lines – you can improve legibility by adding labels at the end of each line rather having a separate legend, or at least having the items of the legend ordered according to the sequence of the lines. I could not align legend to lines, due to too similar colors."

AR: Agreed. We have included numbers at the end of each line to refer to each biological group in Figure 1 and we have ordered the items of the legend according to the sequence of the lines, as suggested. We have also made the y-axis limits narrower to better see the separation between the lines. Besides, we have included also numbers labelling each of the points in Figure 2 and Figure 3.

- **RC 1.10:**

"Fig. 1 - why two groups are estimated for larger scale on x-axis (longer lines)?"

AR: Groups with longer lines (i.e. myctophids and gelatinous zooplankton) are those with at least one pair of stations with slightly shorter transit times, compared to the other set of groups. In the Malaspina survey, sampling was not performed regularly for all groups in all stations.

****

Reviewer #2

- **RC 2.1:**

"I'll first note that the paper is poorly edited, with Figures jumbled in order and often poorly referred to"

AR: Our sincerest apologies to the reviewer. In the latest version of the paper, figures and tables are ordered and well referred.

- **RC 2.2:**

"The Methods section is poorly organized and very confusing. Note first that Figure 1 (line 266) does not refer to the Malaspina Expedition. I believe you are referring to Figure 3 (lines 745-748)?"

AR: Yes. This was a minor typo. In the new version of the paper, there is no specific figure referring to the Malaspina Expedition. We have also reorganized the Methods section to clarify the procedures used (see Response 2.8).

- **RC 2.3:**

"The sampling of diversity I assume is done appropriately to avoid bias, and was done homogeneously throughout the expedition, so I leave consideration of sampling methods for another reviewer (though depth of the neuston sampler could be useful)."

AR: Depth of the neuston sampler = 15 cm. We appreciate the reviewer comment and we have included the following text (line 351) including the neuston sampler depth "Gelatinous zooplankton, macro-zooplankton, surface meso-zooplankton and myctophid fish were sampled using a neuston sampler (80 cm wide, 30 cm high) fitted with a 200 µm mesh size, towed at 2-3 knots during 10-15 minutes at a depth of 15 cm and a distance of 5 m from the starboard side of the hull[23]"

- **RC 2.4:**

"My concerns lie with the scant details provided for identification of recovered diversity. Traditional methods (visual inspection) was used to identify phytoplankton

(surely this is only to a higher taxonomic level, but little additional information is provided), the gelatinous zooplankton (surely a group harboring considerable cryptic diversity!), and lantern fishes; this last group I will hope can be adequately validated by traditional methods. However then, the macrozooplankton were identified with "partial sequences of 16S rDNA and CoxI genes". How? Sanger, or next-generation? How many individuals per site? What were the criteria for species identification, for OTU separation? What was the consideration for cryptic diversity that may not have been previously described and available in genomic databases? None of this information is provided. What quality assessment of data, etc. etc. The same criticism could be applied to the mesozooplankton which were identified using 18S data instead, and thus almost surely lumping together cryptic diversity that cannot be distinguished with such a slow-evolving locus. No information is given on how, methodologically, the 16S rDNA data was collected or analyzed for microbial prokaryotes - was this Sanger? Was it metagenetics, and analysis with Qiime or similar packages? What were the criteria for separation, again? How can these data be referred to as "(unpublished sequences)"? That is not really appropriate for a study like this, any new published study must include some mechanism by which the data will become available; perhaps this is just a placeholder during review.

AR: We agree with the reviewer in that more details on species identification techniques are necessary both for traditional taxonomy and molecular identification. Hence, we have expanded the manuscript to provide additional details and have also provided a new supplementary table (Supplementary table 1) providing information on the description of each group and the methods used for identification and abundance estimation. In addition, we have uploaded the Malaspina occurrence data of each group at each station into the Pangaea open repository (https://www.pangaea.de/) under the following specific link (https://issues.pangaea.de/browse/PDI-14770) (See lines 419-422).

In the new version of the paper we have expanded on the procedures used to describe microbial community composition (see lines 333-341): "About 6 L of seawater were used to determine the composition of microbial communities (marine prokaryotes and small microbial eukaryotes). Water samples were pre-filtered through a 200 µm mesh to remove large plankton, followed by sequential filtration, involving filtering the sample through a 20-µm Nylon mesh followed by a 3 µm pore-size polycarbonate filter (Poretics), and finally through a 0.2 µm polycarbonate filter (Poretics) using a peristaltic pump (MasterFlex 7553-89 with cartridges Easy Load II 77200-62, Cole-Parmer Instrument Company) to collect the prokaryotes and small eukaryotes (size fraction (0.0003 - 0.001 mm). The filters were then flash-frozen in liquid N_2 and stored at −80 °C until DNA extraction".

We have also added the following sentences to provide more information for identification procedures for species and OTUs (see lines 364-367): "Large phytoplankton (dinoflagellates, diatoms and coccolithophores) were identified using inverted microscopy to species level when possible. However, some forms could be only identified to genus (e. g. *Thalassiosira spp*.) or to more general categories like "Small dinoflagellates" or "Small coccolithophores" (see Estrada et al.[24] for more details)". We acknowledge that there are many taxonomic problems with cryptic diversity in the major taxa of gelatinous zooplankton. During the Malaspina 2010

expedition we combined both molecular and morphological taxonomic approaches. To avoid the loss of morphological characters like coloration patterns, we took high-resolution macro pictures of fresh samples [25,26]; but at the same time we preserved samples for DNA extraction according to the protocol of Acuña and Molina-Ramirez[26] and following the recommendations of Dawson et al[27]. We now describe these procedures in the text (see lines 368-375): "Gelatinous zooplankton were identified combining morphological taxonomical approaches and high-resolution photography[23]. The use of molecular approaches in gelatinous zooplankton has many gaps and the most common markers used in widely used techniques as DNA barcoding like COI or ITS are often not useful to resolve all the gelatinous phyla[28]. We confirmed some morphological identifications using mainly DNA barcode with COI as molecular marker. However in groups like Ctenophora or in thaliaceans the identification approach was only based on morphology because the molecular markers were not valid to differentiate between species[28]". Particularly for the macro-zooplankton, the 16S and COI are two markers widely used to identify macro-zooplankton at species level[29]. We have added the following text (see lines 377-393): "Metabarcoding was used to identify macro-zooplankton, epipelagic meso-zooplankton (0-200 m) and microbial communities (prokaryotes and microbial eukaryotes). Specifically, DNA from macro-zooplankton (crustacean, mollusks and insects) was extracted as in Marco-Herrero 2015[30]. Target mitochondrial DNA from the 16S rRNA and Cox1 genes was amplified with polymerase chain reaction (PCR). Primers 1472 (5′- AGA TAG AAA CCA ACC TGG -3′)[31] and 16L2 (5′-TGC CTG TTT ATC AAA AAC AT-3′)[32] were used to amplify 540 bp (base pair) of 16S, while primers COH6 (5′- TAD ACT TCD GGR TGD CCA AAR AAY CA -3′) and COL6b (5′- ACA AAT CAT AAA GAT ATY GG -3′)[32] allowed amplification of 670 bp of Cox1. The PCR products were sent to external Laboratories to be purified and then bidirectionally sequenced (Sanger). Sequences were edited using the Chromas software version 2.0. With the obtained final DNA sequences, a BLAST search was executed on the NCBI webpage (https://www.ncbi.nlm.nih.gov/) to get the sequence that best matched. Macro-zooplankton specimens were identified at species level when sequences fit 100%. Assignations to generic or familial level were made with a 90-99% divergence, depending on taxa and genes analyzed[33]. For lower % of divergence OTUs were kept without taxonomical adscription". For mesozooplankton, we have included the following lines in the text (see lines 393-404): "DNA from meso-zooplankton (0-200 m) samples was extracted following Corell and Rodriguez-Ezpeleta 2014[34]. The V4 of the 18S rRNA gene was amplified using the #1/#2RC primer pair[35] following the "16S Metagenomic Sequence Library Preparation" protocol (Illumina, California, USA). Amplicons were purified using the AMPure XP beads, quantified using Quant-iT dsDNA HS assay kit using a Qubit® 2.0 Fluorometer (Life Technologies, California, USA) and pooled for high throughput sequencing in the Illumina MiSeq platform (Illumina, California, USA). After demultiplexing based on their index, reads were trimmed at 200 bp (as after this position overall Phred quality scores decreased) and processed following the mothur[36] MiSeq SOP[37]. Briefly, sequences with ambiguous bases, chimeras and global singletons were removed, and Operational Taxonomic Units (OTUs) were created by merging reads at 97% similarity". For the microbial communities (prokaryotes and microbial eukaryotes) we have added the following lines (404-419): "Prokaryotic diversity was assessed by amplicon sequencing of the V4-V5 regions of the 16S rRNA gene in the Illumina MiSeq platform (iTags) using paired-end reads (2 × 250 bp) and primers 515F-Y (5′-GTGYCAGCMGCCGCGGTAA) and 926R (5′-CCGYCAATTYMTTTRAGTTT) targeting both Archaea and Bacteria[38]. Small

microbial eukaryotic diversity was assessed by amplicon sequencing of the V4 region of the 18S rRNA gene with the Illumina MiSeq platform using paired-end reads (2 × 250 bp) and the universal eukaryotic primers TAReukFWD1 (5'-CCAGCASCYGCGGTAATTCC) and TAReukREV3 (5'-ACTTTCGTTCTTGATYRA)[39]. For both groups, sequence data processing was performed using an UPARSE[40] based workflow implemented in a local cluster [Marbits platform, ICM] (see Logares 2017[41] for sequence processing details). OTUs were obtained by clustering the sequences at a 97% similarity threshold and taxonomic assignation was performed by blasting (i.e. BLASTn[42]) the sequence representative of each OTU against the 16S SILVA v123[43] and two in-house marine microeukaryote databases based in a collection of Sanger sequences[44] or 454 reads from the BioMarKs project (http://www.biomarks.eu/)".

We are currently in the process of uploading (the data submission is now being checked and processed) the occurrence data of each group into the Pangaea open repository (https://www.pangaea.de/) under the following specific link (https://issues.pangaea.de/browse/PDI-14770) (See lines 419-422) "Occurrence data (presence/absence) of species and OTUs at each station have been submitted to the Pangaea open repository (https://www.pangaea.de/) under the following specific link (https://issues.pangaea.de/browse/PDI-14770)".

We have also included text describing the procedures for abundance estimation of each group (see lines 427-434) "Global abundance determination of each group was carried out using flow cytometer counting[45] (prokaryotes), microscope epi-fluorescense counting (small microbial eukaryotes), inverted microscopy (phytoplankton) and stereo-microscope counting (macro-zooplankton). The abundance of phytoplankton (diatoms 0-160 m, coccolithophores 0-160 m and dinoflagellates 0-160 m) were vertically integrated (0-160 m). Myctophids, gelatinous zooplankton and macro- and surface meso-zooplankton quantification was done using taxonomy identification traditional techniques (Supplementary Table 1)".

- **RC 2.5:**

Finally - note I am still on lines 294-304 alone - the discussion of OTU is of course exactly what is important in this study, so much more consideration and explanation is necessary."

AR: We understand the reviewer concerns into the OTU discussion. We have used the standard protocols for assignment to OTUs or species, which differ slightly from group to group. Microbes are usually grouped at 97-99% of identity in the sequence of their 16S or 18SrRNA, and while there are some instances in which identity at even 100% hides two very different ecotypes, these thresholds have been observed to reflect genome-wide variability, and are those that are commonly used to differentiate one OTU "unit" from another "unit". Same applied to meso- and macrozooplankton species. In any case, the same approach was used for all stations throughout the Malaspina cruise and, thus, our findings should hold independently of the accuracy of OTU or species assignment. We have included the following text, hoping to cover what the reviewer points out (see lines 355-360) "We used the standard protocols for assignment to OTUs (Operational Taxonomic Unit) or species for each group, which may have

slightly differed between groups depending on the taxa. However, the same approach was used for all stations in the Malaspina cruise and, thus, the among-site similarity of each group should hold independently of the exactness of OTU or species assignment. The following paragraph describes in detail the assignment methods".

- **RC 2.6:**

"First please note the equations in your manuscript are barely readable in the PDF provided. I'm also concerned that equation (1), for Jaccard dissimilarity, does not match the information I have for this index (Jost et al Chapter 6 in Magurran & McGill 2011. This same resource notes that in a study like this, multiple-site versions of this statistic should be applied because of the non-independence of multiple data points; it also suggests that the Jaccard index is less appropriate than the multiple-site version of the Sørensen index, but my main concern is the wrong version being applied"

AR: We must apologize. There was a major typo in equation (1). In our initial submission we wrote down Colwell and Collingtown[46] beta-diversity measure (see Koleff et al.[47]) by mistake, instead of the Jaccard similarity index, but we have calculated beta-diversities among groups using the correct Jaccard similarity index, as in Magurran & McGill[48]. We have corrected this error, and corrected the formula in the revised version of the manuscript (see equation 1 line 460). In terms of the metric used, we have calculated the similarities considering the number of shared species of each particular group at each pair of sites, that is, one site against the other, comparing two assemblages every time. This is in order to compare to pair-wise transit times driven by oceanographic currents, using a Mantel test between the two distance/similarity matrices. For that reason, we consider that for the scope of the paper, the pair-wise Jaccard metric used is more appropriate than the multiple-site-version similarity indices (Jaccard or Sorensen). In the revised version of the paper, we have also made equations larger in font size, hoping that now they will be easily readable.

- **RC 2.7:**

"it is not clear how the plastics data inform this analysis, as there is no relationship between size and scale of dispersal in plastics, but those "OTUs" are artificially defined by color, which has nothing to do with environmental context or point of origin. In fact, it isn't clear to me why the slope for plastics isn't zero?"

AR: According to the reviewer's comment, we have excluded the micro-plastic data from the analysis. See response 1.7.

- **RC 2.8:**

"I am concerned that what is being used in this paper is a very local-scale Lagrangian model in a heavily studied region of the world for these dynamics (the Southern California Bight). Whether similar methods apply to the global models, at larger scales, in particular when applied to a linear transect for which different points on the transect are likely to have non-linear and multiple paths of connectivity, is quite concerning that so little explanation is given for how one method has been scaled up from an area of about 10,000km^2 to global scales."

AR: We apologize. There was a confusion in the reference list, where we cited earlier regional work on connection by co-author Watson (Watson 2011[49]). For this paper, we used Watson and Jonsson's latest connectivity product (Jonsson and Watson 2016[21]) which was developed specifically for global scales. We have updated the text to reflect this, and in our methods, which has a brief description of Watson and Jonsson's (Jonsson and Watson 2016[21]) approach. See also response to reviewer 3.4.

- **RC 2.9:**

"The organization remains a problem in the rest of the methods; "distance-decay slopes" are explained 2 sections after the section called "Halving-distance and distance-decay slope" (starting on line 354).

AR: We agree with the point raised by the reviewer and we appreciate the suggestion of reorganizing the methods section to facilitate the understanding of the concepts. For that reason, in the revised version of the manuscript, we have merged the methods sections "Halving-Distance and distance decay slope" and "Dispersal scales and species turnover" into the "Connectivity descriptors: Halving-Distance and Distance decay Slopes" section, trying to keep the concepts clear, and in a consecutive manner. As such, we have reorganized the methods section in a more logical sequence: 1) description of the "Biological dataset", 2) the "Distance and similarity matrices, 3), the "Correlations of species turnover with currents and environmental predictors", and 4) the "Connectivity descriptors: The Halving distance and Distance decay slopes". In the latter point 4, (i) the definition of the distance decay and the formulae used is given, followed by (ii) the Halving-time definition and formulae; 5) Finally, the methods used to explore spatial patterns of β-diversity are described.

- **RC 2.10:**

I will say that I do like the idea of using plastic particles as a null approach, but even this (separating into 16 colors as "species") brings me some concern later when looking at results"

AR: We understand the concerns related to plastic colors/pseudo-OTU assignation, and because of that and previous comments (Comment 2.7 and 1.7), we decided to exclude them from the analysis; See response 1.7 for more explanations.

- **RC 2.11:**

"(Line 119). I assume on Line 123 you mean "significantly negatively correlated".

AR: Agreed and changed.

- **RC 2.12:**

"These results are intriguing when stated, but the effect sizes are hard to interpret. The "halving times" for microbial diversity are on the order of 10^6 days, or thousands of years, perhaps longer than current estimates of the total turnover time of any point in the surface ocean? Here again, references to Tables (line 149) are incorrect. Even if the

halving time for macrozooplankton is 207 days (an interesting and more relevant figure). There may indeed be a size factor involved in these distributions (not shown in "Table 5" referred to in line 159)"

AR: We appreciate this positive comment and we can understand that the effect sizes are difficult to cope with, particularly for small-bodied groups. For example, the time-decay slope of genetically identified dinoflagellates (Dinoflagellates surface) (-0.0046) is the flattest within all groups, which in turn results in a halving-time (*ca.* $1.5x10^7$ years) that goes beyond the transit time domain of the earth. This is basically because, mathematically, it would take a lot of time for the initial similarity (in adjacent sites) to decline to half. However, in some groups such as the dinoflagellates, their similarity is never less than half of the initial similarity, even for stations located far apart. To clarify this, we have included the following sentences in the text (see lines 223-231) "Notably, the large halving-times of marine microbial organisms imply that, when dispersing with ocean currents, it would take thousands of years of oceanic transport for such communities to halve the similarity between adjacent sampling stations (i.e. the initial similarity). However, in some biological groups, such as dinoflagellates, their community similarity is never less than half of the initial similarity, even for stations located far apart. As such, the halving-time is a relative indicator, or proxy, of community dispersal scale, and should not be interpreted as an absolute value of the transit time that operates among the sub-communities". Else, to make the numbers more easily interpretable we have converted the halving-times from days to years (see table 2A). We apologize for the poor edition of tables and figures. In the revised version of the manuscript the figures and tables are well referred to.

- **RC 2.13:**

"it is not clear how the plastics data inform this analysis, as there is no relationship between size and scale of dispersal in plastics, but those "OTUs" are artificially defined by color, which has nothing to do with environmental context or point of origin. In fact, it isn't clear to me why the slope for plastics isn't zero?"

AR: According to the reviewer comment 2.7, we have excluded the micro-plastic data from the analysis. See response in RC 1.7.

- **RC 2.14:**

"Given the concerns raised above, I left the Discussion alone. I think this is an astounding data set (though I don't know most of the details), and a very interesting question. In many ways, the methods seem appropriate; however, in many other ways the degree of hand-waving and incautious consideration of the complexity of this problem (what is diversity? how is it distributed? how do environmental forcing mechanisms distinct from ocean currents interact with those ocean currents to drive these distributions?) makes me concerned that we don't yet know the answer from these data."

We have addressed all the technical points raised by the referee, included new analyses in relation to abundance data, better defined the concepts, used consistent terminology,

and provided comprehensive information on methods and results in order to clarify our findings. In particular, we have analyzed the relationship between abundance and dispersal scale (halving time and time-decay slope) for each group, and we have shown that there is a significant, yet slightly stronger correlation relative to the size vs. dispersal scale (see Fig. 3 and Table 3A). We have also referred to the metabolic theory of ecology[1], as a framework that helps explain the ecological processes driving dispersal. In order to make it clear, the methods section has been re-structured and we have included more methodological detail, both in the OTU identification and counting, and some basic rationale behind the modelling approach to explain the surface ocean transit times. Our conclusion is now more clear and better supported (see lines 58-65) "Our results reveal that β-diversity is significantly negatively correlated with the surface ocean transit times, more so than with differences in environmental factors. We also find that less abundant, large-bodied plankton and micro-nekton communities in near-surface epipelagic waters have significantly shorter dispersal scales and larger spatial species-turnover rates when compared to small-bodied and higher abundant plankton. These results confirm that the dispersal scale of planktonic and micro-nektonic organisms is determined by local abundance, which scales with body size, ultimately setting global patterns of diversity". We hope this revised version of the manuscript addressed appropriately the reviewer's concerns.

****

Reviewer #3

- **RC 3.1:**

"I therefore think the manuscript needs improving in two ways: A) Restructuring and clarification for a general scientific audience. B) Additional methodological detail."

AR: We thoroughly revised the manuscript. We have tried to clarify concepts (by including specific definitions) and objectives in the introduction for a wider scientific audience. We have also tried to restructure the text to make our scientific question clearer separating the two strands of the study: 1) Identifying what drives species distribution, 2) Calculating size classes dispersal scales, provided that currents are the main process driving distribution (see lines 124-130). We have also provided additional methodological details regarding both species and OTU identification and counting (see RC 2.4), and some basic rationale behind the modeling approach to explain the surface ocean transport times (see RC 2.9).

- **RC 3.2:**

"Define and be consistent with your use of the terms 'dispersal', 'connectivity' and 'ocean transit times', as these are often used in slightly different contexts in other publications and could be confused for one another in this manuscript. I think of 'dispersal' as the transport of an individual by ocean currents (what you call

connectivity), whereas what I think you mean by dispersal scales here is more akin to species range (i.e. colonisation of increasingly wider area over multiple generations, until equilibrium is reached). This distinction between biogeography versus short-term processes is an important one. Your use of the term connectivity is also not clear. For example, in lines 182-183 you state that connectivity is determined by both transport by ocean currents (what I think of as dispersal) plus environmental filtering. I would say this was the correct definition of connectivity However, you then state that "ocean connectivity (through our estimates of surface ocean transit times) explains a larger fraction of the variability in… community similarity, relative to environmental factors" (lines 184-187).

AR: We understand the concerns raised by the reviewer and we apologize for the inconsistency and for messing up the concepts. We have addressed this by defining *dispersal* as "the movement of individuals across space[50]" (lines 71-72), *biological connectivity* as "the exchange of individuals among geographically separated subpopulations[50] (lines 70-71), and *surface ocean transit time* as "the shortest time taken for water to travel from one patch in the surface ocean to another[21]" (line 479-480). To avoid further confusions, we have also replaced the term "timescales of ocean connectivity" for "surface ocean transit time" in the new text.

- **RC 3.3:**

I would therefore recommend clearly defining and separating the two metrics; a) scales of dispersal (determined empirically based on species composition, i.e. the distance-decay slopes) and b) timescales of surface ocean connectivity/ocean transit times (derived from a previous modelling study), and how they are derived early on in the manuscript. Make it clear throughout the manuscript that only "surface ocean transit time/connectivity" is modelled. On first reading the manuscript I mistakenly assumed that you had determined the dispersal scales of the different size plankton groups using the Lagrangian particle simulation approach. You don't state that you are determining distance decay slopes until line 107, and then fail to link these to the "dispersal scales" discussed in the abstract. With this is mind, I think the structure of the introduction could be improved to make your aims and the hypotheses you are testing clearer to the reader"As I understand it, your main aim was to test whether scales of dispersal vary amongst different size groups. You do this by 1) firstly testing how much of the variation in β-diversity can be explained by oceanographic distance (modelled surface ocean transport times) over changes in environmental variables (despite the fact that, as you state at lines 91-94, the two are often correlated). Given that this tells you that β-diversity is indeed predominantly controlled by oceanographic distance, you then 2) reverse the process - using patterns of β-diversity to estimate how far different size classes of organisms are able to disperse (given the caveat that a proportion of the limitation to dispersal will still be environmentally driven). This took me a while to get at, and was not clear from the introduction alone. I think the two strands of the study should be more clearly separated"

AR: Following the reviewer advice, we have modified the text in order to separate the two metrics a) scales of dispersal and b) surface ocean transit times, and clarify that only the latter is modeled (lines 120-130) "Here, we quantify empirically derived distance-decay slopes and measure the spatial scales of dispersal for a number of planktonic and micro-nekton organisms ranging greatly in body size and abundance,

from prokaryotes to small mesopelagic fishes, and test the hypothesized size-dependence of community dispersal scales and resulting spatial patterns of regional connectivity. To do so, first we test the importance of surface ocean transit times, derived from previous Lagrangian particle simulations[21] (see methods), in explaining spatial patterns of β-diversity for each group, accounting for the relative contribution of environmental filtering[51]; second, since β-diversity has been found to be mainly controlled by surface ocean transit time, we use the distance-decay slopes of each biological group to infer the community dispersal scale as a proxy of distribution range (*sensu* biogeography".

- **RC 3.4:**

"Regarding detail on the methodology, you state that for the surface ocean transit time matrix (lines 345-352) you "[used] the approach of Watson et al. [2011]", but do not give any specifics of the model configuration for this study (Figure 5, presumably referring to S5, does not give any detail). I therefore cannot comment on this approach here. For example, what spatial and temporal scales did you model particle transport over? What depths? I think it would improve the interpretation of your results if you explicitly stated how you derived the ocean transport times - for example that you did not consider behavior (e.g. swimming) or depth - and how this may have affected your correlation between ocean transport times (your 'connectivity') and β-diversity. "

AR: We apologize for the error. There was a mistake in the literature cited, where we cited earlier regional work on connectivity by co-author Watson (Watson 2011[49]). In this paper, we actually used Watson's latest connectivity work (Jönsson and Watson 2016[21]) which was developed specifically for global scales. We have updated the text to reflect this, and our methods, which now include a brief description of Jönsson and Watson's approach (see lines 478-489): "To calculate the particle transit times between any two points in the ocean, that is, the shortest time taken for water to travel from one patch in the surface ocean to another, a Lagrangian particle simulation model was used. Specifically, we used velocity fields from the ECCO2 (http://ecco2.org), a high resolution global model (1/4ºx1/4º) that assimilates satellite and in-situ data to advect particles in the surface ocean. Particles were seeded over 9 years and advected for 100 years by looping fields for the years 2000–2010. The resulting paths were used to estimate the minimum transit times. Minimum connectivity times were then calculated by aggregating the ECCO2 grid cells into 8*8º patches, each approximately 2*2º in size. The second depth layer of each ECCO2 grid cell was used to seed the particles (36 million particles in total over all seeding times). The second depth layer is between 5 and 20 m depth (see Jönsson and Watson 2016[21] for further model details)".

We agree with the reviewer that some of our groups were sampled from surface to 200 m (meso-zooplankton) and from surface to 160 m (phytoplankton) depths, and that our particle-tracking model resolves only the 5-20 m depth. However, the highest zooplankton and phytoplankton concentrations are expected to be found in the upper ocean layer (upper 100 m). The other set of planktonic groups have been sampled at the surface (0-3 m), allowing the use of surface particle-tracking model is appropriate to explore surface connectivity patterns. We also agree with the reviewer that the myctophids' swimming behavior (the only actively swimming group studied) might affect the correlation between community similarity and transport time. In fact, the

movement of myctophid juveniles and adults mostly follow a diel vertical migration (rather than horizontal), making this group a bit difficult to cope with in terms of connectivity, but they also may drift as larvae (with an average drift duration from 30 to 60 days[52]), and hence, they behave as passive tracers, being subject to horizontal transport at surface. Myctophid larvae are passively advected in the epipelagic waters they occupy along with other macro-zooplankton[53], which may result in their export away from their home range by currents[54,55]. It must also be considered that the mesopelagic fish included in the study were sampled at the surface. The similar dispersal patterns of myctophids and macro-zooplankton may arise from the fact that both have some swimming capacity mostly used for diel vertical migrations. However, the other set of groups all are passive drifters that may be adequately represented by ocean surface transit times. We now address this in the text, which now reads (see lines 491-499) "The estimation of dispersal scales, using modelled surface ocean transit times, may be appropriate as a first order approximation for the dispersal of planktonic organisms, but it will be less so for other larger biological groups, particularly the myctophids which, as we have discussed, actively migrate vertically. There are numerous alternatives to modeling the dispersal of actively swimming marine organisms, ranging from agent-based models to advection-diffusion type methods to the simple use of great-circle distances between locations. However, for our study of the spatial patterns in planktonic communities at a global scale, using ocean transit times derived from the dispersion of passive surface Lagrangian particles was sufficient".

- **RC 3.5:**

"For example, you state that body size is not the sole driver of 'dispersal', discussing also population densities (population size at line 101) and generation times (lines 205-214) - could differing depth distributions between groups explain some of your missing variation in community structure (i.e. differing transport due to changes in currents with depth)? (In my earlier misunderstanding of your approach, on reading lines 62-64 of the abstract, in which you state "larger-bodied plankton… in near-surface epipelagic waters have significantly shorter dispersal scales", I found myself wondering whether the different dispersal scales were indeed due to differing body size, or alternatively due to their depth distribution)"

AR: We have only modeled surface currents, i.e. 5-20 m, and most of the groups have been sampled at surface in the Malaspina survey (see also response 3.4). For these organisms, distribution is mainly driven by the differences in body size or abundance between each biological group (see also response RC 1.1).

- **RC 3.6:**

"It is not clear to me how you defined microplastic community composition/distance-decay relationships to compare with the various plankton groups."

AR: As stated previously (see response 1.7), we excluded micro-plastics.

Minor comments

- **RC 3.7:**

Define specialist terms in the main text: β-diversity (line 81), halving time (line 142), spatial turnover rates (line 142).

AR: Done. We have defined *β-diversity* as "The shift in species composition among locations[56]" (line 82), *halving-time* as ", the oceanic transit time at which species similarity halves[57]" (lines 153-154) and *spatial turnover rates* as "the rate of species turnover per unit distance[58]" (lines 532-533) or as distance-decay rates (lines 85-87) "measured as the slope of a linear relationship between the logarithm of community similarity and the logarithm of Euclidean distance among pairs of sites[58]".

Line 60/65: A pet hate of mine maybe, but I recommend avoiding terminology such as 'reveal' and 'confirm', replacing it with something like 'suggest' or 'provide evidence that'. Also "of course" on line 173.

AR: Agreed. We have avoided these "categorical" verbs where possible. We have deleted "the of course" term from the text, as we considered it does not tell the reader nothing much.

Lines 99-105: The rationale for the argument that smaller organisms are likely to disperse further is not clear to me from this paragraph. Could you perhaps expand/simplify this hypothesis, using the references you cite?

AR: See also response 1.1. We have tried to expand the hypothesis, adding new text and introducing new frameworks such as the metabolic theory of ecology[1] to link abundance, generation time, body size and dispersal mode to unravel differences in dispersal scale patterns. First, we have added a sentence to make clear that we are dealing with passively dispersing taxa (lines 110-111) "Among smaller, mostly passively dispersed taxa, body size is expected to be inversely correlated with dispersal ability". Second, we formulate the hypothesis that dispersal limitation increases with body size (lines 111-112). Third, based on the new analysis, we conclude that smaller-bodied plankton are more abundant and probably have lower generation time rates, hence they are less prone to stochasticity and ecological drift (lines 112-115). To make it clear, we have emphasized the importance of abundance shaping distribution (lines 112-115) "That is, smaller organisms have in general larger population sizes[11], they are much more abundant compared to large organisms, hence should yield lower local extinction rates[7] and therefore, reduced demographic stochasticity and ecological drift[59]. And fourth, we indicate that, for the aforementioned set of reasons, small planktonic organisms are capable to be dispersed with oceanic currents, compared to passively dispersed larger taxa (lines 115-118) "Further, smaller more abundant organisms are likely to disperse further with oceanic currents for example[60], leading to shallower distance-decay slopes when compared to those of larger organisms[61-64]".

.

Line 150: Large-bodied groups?

AR: Agreed and changed.

Some typos: Line 84: “by as a”? Line 92: “weather”. Line 98: “,”. Line 352: “groups”.

AR: Agreed and changed.

In addition to the response to reviewer, during the review process we have included new data, and the size of each biological group has been updated. These small changes, however, do not affect the observed dispersal vs. size relationship.

References

1 Brown, J. H., Gillooly, J. F., Allen, A. P., Savage, V. M. & West, G. B. Towards a metabolic theory of ecology. *Ecology* **85**, 1771-1789, doi:10.1890/03-9000 (2004).

2 Peters, R. H. *The Ecological Implications of Body Size*. (Cambridge University Press, 1986).

3 Andersen, K. H. *et al.* Characteristic Sizes of Life in the Oceans, from Bacteria to Whales. *Annual review of marine science* **8**, 217-241, doi:10.1146/annurev-marine-122414-034144 (2016).

4 Gillooly, J. F., Charnov, E. L., West, G. B., Savage, V. M. & Brown, J. H. Effects of size and temperature on developmental time. *Nature* **417**, 70-73 (2002).

5 Kirchman, D. L. Growth Rates of Microbes in the Oceans. *Annual review of marine science* **8**, 285-309, doi:10.1146/annurev-marine-122414-033938 (2016).

6 Andersen, K. H. & Beyer, J. Asymptotic size determines species abundance in the marine size spectrum. *American Naturalist* **168**, 54-61, doi:10.1086/504849 (2006).

7 Pimm, S. L., Jones, H. L. & Diamond, J. On the Risk of Extinction. *The American Naturalist* **132**, 757-785 (1988).

8 Blackburn, T. M. & Gaston, K. J. *Macroecology: Concepts and Consequences: 43rd Symposium of the British Ecological Society*. (Cambridge University Press, 2003).

9 MacArthur, R. H. & Wilson, E. O. *The Theory of Island Biogeography*. (Princeton University Press, 1967).

10 Whitfield, J. Is Everything Everywhere? *Science* **310**, 960 (2005).

11 Sheldon, R. W., Prakash, A. & Sutcliffe, W. H. The size distribution of particles in the ocean. *Limnology and Oceanography* **17**, 327-340, doi:10.4319/lo.1972.17.3.0327 (1972).

12 Soininen, J., Lennon, J. J. & Hillebrand, H. A multivariate analysis of beta diversity across organism and environments. *Ecology* **88**, 2830-2838, doi:10.1890/06-1730.1 (2007).

13 Bell, G. Neutral Macroecology. *Science* **293**, 2413-2418, doi:10.1126/science.293.5539.2413 (2001).

14 Maranon, E. Cell size as a key determinant of phytoplankton metabolism and community structure. *Annual review of marine science* **7**, 241-264, doi:10.1146/annurev-marine-010814-015955 (2015).

15 Tang, E. P. Y. The allometry of algal growth rates. *Journal of Plankton Research* **17**, 1325-1335, doi:10.1093/plankt/17.6.1325 (1995).

16 Rinaldo, A., Maritan, A., Cavender-Bares, K. K. & Chisholm, S. W. Cross-scale ecological dynamics and microbial size spectra in marine ecosystems. *Proceedings of the Royal Society B: Biological Sciences* **269**, 2051-2059, doi:10.1098/rspb.2002.2102 (2002).

17 Kiørboe, T. & Hirst, A. G. Shifts in Mass Scaling of Respiration, Feeding, and Growth Rates across Life-Form Transitions in Marine Pelagic Organisms. *The American Naturalist* **183**, E118-E130, doi:doi:10.1086/675241 (2014).

18 Bienfang, P. K. Phytoplankton sinking rates in oligotrophic waters off Hawaii, USA. *Marine Biology* **61**, 69-77, doi:10.1007/bf00410342 (1980).

19 Finkel, Z. V. *et al.* Phytoplankton in a changing world: cell size and elemental stoichiometry. *Journal of Plankton Research* **32**, 119-137, doi:10.1093/plankt/fbp098 (2010).
20 Farjalla, V. F. *et al.* Ecological determinism increases with organism size. *Ecology* **93**, 1752-1759, doi:10.1890/11-1144.1 (2012).
21 Jonsson, B. F. & Watson, J. R. The timescales of global surface-ocean connectivity. *Nat Commun* **7**, doi:10.1038/ncomms11239 (2016).
22 Cózar, A. *et al.* Plastic debris in the open ocean. *Proceedings of the National Academy of Sciences* **111**, 10239-10244, doi:10.1073/pnas.1314705111 (2014).
23 Molina-Ramírez, A. *et al.* Functional differences in the allometry of the water, carbon and nitrogen content of gelatinous organisms. *Journal of Plankton Research*, doi:10.1093/plankt/fbv037 (2015).
24 Estrada, M. *et al.* Phytoplankton across Tropical and Subtropical Regions of the Atlantic, Indian and Pacific Oceans. *PLoS ONE* **11**, e0151699, doi:10.1371/journal.pone.0151699 (2016).
25 Svoboda, A. Close-up photography of marine life on board research vessels. *Scientia Marina* **56**, 239-244 (1992).
26 Acuña J.L & Molina-Ramírez, A. in *Expedición Malaspina 2010. Cambio Global y Exploración de la biodiversidad del óceano. Libro blanco de métodos y técnicas de trabajo ocenaográfico* (ed E Moreno Ostos) 623-645 (2011).
27 Dawson M.N, K.A. Raskoff & Jacobs, D. K. Preservation of marine invertebrate tissues for DNA analyses. *Molecular Marine Biology and Biotechnology* **7**, 145-152 (1998).
28 Bucklin, A., Steinke, D. & Blanco-Bercial, L. DNA barcoding of marine metazoa. *Annual review of marine science* **3**, 471-508, doi:10.1146/annurev-marine-120308-080950 (2011).
29 Schubart, C. D. N., J.E. . The use of the mitochondrial 16S rRNA gene for phylogenetic and biogeographic studies of Crustacea. **12**, 817–830 (2000).
30 Marco-Herrero, E., González-Gordillo, J. I. & Cuesta, J. A. Larval morphology of the family Parthenopidae, with the description of the megalopa stage of Derilambrus angulifrons (Latreille, 1825) (Decapoda: Brachyura), identified by DNA barcode. *Journal of the Marine Biological Association of the United Kingdom* **95**, 513-521, doi:10.1017/s0025315414001908 (2015).
31 Crandall, K. A. & Fitzpatrick, J. J. F. Crayfish Molecular Systematics: Using a Combination of Procedures to Estimate Phylogeny. *Systematic Biology* **45**, 1-26, doi:10.1093/sysbio/45.1.1 (1996).
32 Schubart, C. D., Cuesta, J. A. & Felder, D. L. Glyptograpsidae, a New Brachyuran Family from Central America: Larval and Adult Morphology, and a Molecular Phylogeny of the Grapsoidea. *Journal of Crustacean Biology* **22**, 28-44, doi:doi:https://doi.org/10.1163/20021975-99990206 (2002).
33 Costa, F. O. *et al.* Biological identifications through DNA barcodes: the case of the Crustacea. *Canadian Journal of Fisheries and Aquatic Sciences* **64**, 272-295, doi:10.1139/f07-008 (2007).
34 Corell, J. & Rodríguez-Ezpeleta, N. Tuning of protocols and marker selection to evaluate the diversity of zooplankton using metabarcoding. *Revista de Investigacion Marina* **21**, 19-39 (2014).
35 Machida, R. J. & Knowlton, N. PCR Primers for Metazoan Nuclear 18S and 28S Ribosomal DNA Sequences. *PLoS ONE* **7**, e46180, doi:10.1371/journal.pone.0046180 (2012).

36 Schloss, P. D. *et al.* Introducing mothur: Open-Source, Platform-Independent, Community-Supported Software for Describing and Comparing Microbial Communities. *Applied and Environmental Microbiology* **75**, 7537-7541, doi:10.1128/aem.01541-09 (2009).

37 Kozich, J. J., Westcott, S. L., Baxter, N. T., Highlander, S. K. & Schloss, P. D. Development of a Dual-Index Sequencing Strategy and Curation Pipeline for Analyzing Amplicon Sequence Data on the MiSeq Illumina Sequencing Platform. *Applied and Environmental Microbiology* **79**, 5112-5120, doi:10.1128/aem.01043-13 (2013).

38 Parada, A. E., Needham, D. M. & Fuhrman, J. A. Every base matters: assessing small subunit rRNA primers for marine microbiomes with mock communities, time series and global field samples. *Environmental Microbiology* **18**, 1403-1414, doi:10.1111/1462-2920.13023 (2016).

39 Stoeck, T. *et al.* Multiple marker parallel tag environmental DNA sequencing reveals a highly complex eukaryotic community in marine anoxic water. *Mol Ecol* **19 Suppl 1**, 21-31, doi:10.1111/j.1365-294X.2009.04480.x (2010).

40 Edgar, R. C. UPARSE: highly accurate OTU sequences from microbial amplicon reads. *Nat Meth* **10**, 996-998 (2013).

41 Workflow for analysing MiSeq amplicons based on Uparse v1.5 (2017).

42 Altschul, S. F. *et al.* Gapped BLAST and PSI-BLAST: a new generation of protein database search programs. *Nucleic Acids Research* **25**, 3389-3402 (1997).

43 Quast, C. *et al.* The SILVA ribosomal RNA gene database project: improved data processing and web-based tools. *Nucleic Acids Research* **41**, D590-D596, doi:10.1093/nar/gks1219 (2013).

44 Pernice, M. C., Logares, R., Guillou, L. & Massana, R. General Patterns of Diversity in Major Marine Microeukaryote Lineages. *PLoS ONE* **8**, e57170, doi:10.1371/journal.pone.0057170 (2013).

45 Gasol, J. M. & Morán, X. A. G. in *Hydrocarbon and Lipid Microbiology Protocols: Single-Cell and Single-Molecule Methods* (eds Terry J. McGenity, Kenneth N. Timmis, & Balbina Nogales) 159-187 (Springer Berlin Heidelberg, 2016).

46 Colwell, R. K. & Coddington, J. A. Estimating Terrestrial Biodiversity through Extrapolation. *Philosophical Transactions of the Royal Society of London. Series B: Biological Sciences* **345**, 101-118, doi:10.1098/rstb.1994.0091 (1994).

47 Koleff, P., Gaston, K. J. & Lennon, J. J. Measuring beta diversity for presence–absence data. *Journal of Animal Ecology* **72**, 367-382, doi:10.1046/j.1365-2656.2003.00710.x (2003).

48 Magurran, A. E. *Measuring biological diversity*. (John Wiley & Sons, 2013).

49 Watson, J. R. *et al.* Currents connecting communities: nearshore community similarity and ocean circulation. *Ecology* **92**, 1193-1200, doi:10.1890/10-1436.1 (2011).

50 Cowen, R. K., Gawarkiewicz, G., Pineda, J., Thorrold, S. R. & Werner, F. E. Population Connectivity in Marine Systems. *Oceanography* **20** (2007).

51 Legendre, P. & Legendre, L. F. J. *Numerical Ecology*. (Elsevier Science, 2012).

52 Conley, W. J. & Gartner, J. J. V. Growth Among Larvae of Lanternfishes (Teleostei: Myctophidae) from the Eastern Gulf of Mexico. *Bulletin of Marine Science* **84**, 123-135 (2009).

53 Olivar, M. P. *et al.* Diel-depth distributions of fish larvae off the Balearic Islands (western Mediterranean) under two environmental scenarios. *Journal of Marine Systems* **138**, 127-138 (2014).
54 Hulley, P. A. The South African Museum's Meiring Naude Cruises. Part 14. Family Myctophidae *Annals of The South African Museum* **93**, 53--96 (1984).
55 Flynn, A. J. & Marshall, N. J. Lanternfish (Myctophidae) Zoogeography off Eastern Australia: A Comparison with Physicochemical Biogeography. *PLoS ONE* **8**, e80950, doi:10.1371/journal.pone.0080950 (2013).
56 Whittaker, R. H. Vegetation of the Siskiyou Mountains, Oregon and California. *Ecological Monographs* **30**, 279-338, doi:10.2307/1943563 (1960).
57 Soininen, J., McDonald, R. & Hillebrand, H. The distance decay of similarity in ecological communities. *Ecography* **30**, 3-12, doi:10.1111/j.0906-7590.2007.04817.x (2007).
58 Nekola, J. C. & White, P. S. The distance decay of similarity in biogeography and ecology. *Journal of Biogeography* **26**, 867-878, doi:10.1046/j.1365-2699.1999.00305.x (1999).
59 Hubbell, S. P. *The Unified Neutral Theory of Biodiversity and Biogeography (MPB-32)*. (Princeton University Press, 2001).
60 Finlay, B. J., Esteban, G. F. & Fenchel, T. Global diversity and body size. *Nature* **383**, 132-133 (1996).
61 Salazar, G. *et al.* Global diversity and biogeography of deep-sea pelagic prokaryotes. *ISME J*, doi:10.1038/ismej.2015.137 (2015).
62 Finlay, B. J. Global Dispersal of Free-Living Microbial Eukaryote Species. *Science* **296**, 1061-1063, doi:10.1126/science.1070710 (2002).
63 Martiny, J. B. H. *et al.* Microbial biogeography: putting microorganisms on the map. *Nat Rev Micro* **4**, 102-112 (2006).
64 De Wit, R. & Bouvier, T. ‘Everything is everywhere, but, the environment selects’; what did Baas Becking and Beijerinck really say? *Environmental Microbiology* **8**, 755-758, doi:10.1111/j.1462-2920.2006.01017.x (2006).

Reviewers' comments:

Reviewer #1 (Remarks to the Author):

The manuscript overall improved considerably compared to the previous submission. However, the language of the text needs to be checked carefully, there are many long sentences and syntax errors (not commented below).
My only major concern is that the authors fail to address a conflicting finding of the study, namely finding on the one hand weaker distance decay in microbes and protists, while they could not show a stronger (local) environmental control in these groups (as should be expected, following hypothesis 1). This can be interpreted that some important env parameters are missing. More specifically, the smallest groups exhibit pronounced short-term (seasonal) fluctuations in their community composition, which might not be represented well in the available env data. It may, hoever, also point at limitations regarding the quantification of connectivity, as transit times (e.g. roles of considering regional population sizes are not considered).

Abundances are invoked as a 'mechanism' supporting higher connectivity in small(=abundant) organisms, yet the mechanism isnt specified (the effectve population size should consider biovolume, not cellular abundance). Possession of resting stages (in microbes & protists) & low sinking losses seems to be a more valid argument than cellular abundance.

Specific comments

L 53 "we quantified the dispersal scale and community structure – β-diversity" – community structure is not beta diversity (insert 'spatial' before community structure)

l. 71-74 – split sentence (structure)

l. 76-77 – why differences in _expected_ body size?
I'm missing a consideration that you address passively dispersing organisms in this paragraph. Reg comp with terr habitats (which are spatially structured), I doubt you can readily derive expectations for organisms drifting in a continuum, unless you address explicitly passive dispersers

l. 98f – overall, there is a surely correlation between spatial and env distance, but this may have various shapes - not necessarily proportional (would require to be linear on log-log scale)
l. 103 f – there is surely a robust (=general) distance decay pattern across organisms (see reviews by Hillebrand and others), you rather mean that slopes differ across phylogenetic groups(?)

l 108 – metabolic rates scale with growth rates, small organisms have _high_ rates

l 110. – abundant in terms of cellular/organismal density or biomass? Quite a difference when comparing e.g. viruses with phytoplankton and bacteria
l. 117 ff – "Further, smaller more abundant organisms are likely to disperse further with oceanic currents" – why? The extinction argument was made before, what else would support farther dispersal (among passive dispersers)

l. 146 – finding a low correlation between spat and env distance is in contrast to expectations and should be highlighted more

l. 171 – importance of distance per se depends on quality of env data
l. 246 – linking dispersal to generation time is acceptable as mechanism – but what is the mechanism underlying the abundance argument?
L 297 – this paragraph isn't very clear (high amount of shared taxa, simultaneously high beta div) –" sample sites between

298 subtropical gyres of the Atlantic, Pacific and Indian Oceans are extremely well
299 connected in terms of species shared (i.e. acting as bridges between ocean provinces)
300 with relatively high β-diversityhigh beta diversity are reported together with high connectivity”

Reviewer #2 (Remarks to the Author):

Though I really appreciated the novelty of using oceanic micro plastics in some form of null inquiry in the previous version, I agree with your decision to remove this from the current manuscript. I think it is considerably better now, though there are still a number of proofreading errors and so please check it again with a fine-toothed comb! Otherwise I feel that my concerns have been addressed, this is a nice contribution.

Reviewer #3 (Remarks to the Author):

The manuscript is now greatly improved. I appreciate that the authors have clarified their terminology, restructured the manuscript and provided more methodological detail. I also agree with the decision to remove microplastics from the analysis. The manuscript requires minor edits for further clarity (detailed below) and grammatical issues. Note that I am unable to comment on the appropriateness of the biological and statistical content of the paper, which I leave for other reviewers.
1. ECCO2 should be described as a global ocean circulation model as opposed to simply a global model. Secondly, I would not describe 1/4° as ‘high resolution’, for example when compared to the HYCOM global reanalysis circulation output which is provided at 1/12° 3-hourly or daily resolution on a global scale for the period 1992-present. The vertical resolution of the ECCO2 fields is also low if velocities are given as an average between 5 and 20m depth.
2. I still feel more detail could be added on the modelling approach; What dispersal modelling system did you use? (n.b. a comma should be added in line 488 before ‘advect’ to make it clear that ECCO2 does not do the particle advection itself but is instead used as input data). What is the temporal resolution of the ECCO2 velocity fields (which is as, if not more, important in influencing modelled patterns of dispersal than spatial resolution)? How frequently did you ‘seed’ particles? Presumably the model was run in 2D and particles weren’t able to move in the vertical? Even if you don’t feel this detail to be important in the methods, as it refers to a previous study, these considerations should be still be discussed as potential limitations of the underlying modelled surface ocean transit times. Finally, you do not cite Jonsson and Watson 2016 until the last line, where it looks instead like it refers to the depth layer of the ECCO2 output.
3. The new paragraph at lines 183-193 seems a bit out of place – I’m not sure what you are trying to say here and why this is important to the results, especially as it refers to previously published results. Perhaps it could be shortened/simplified and merged with the following paragraph? Further, I’m not sure how you conclude from this figure that the Atlantic is ‘less connected’ than other basins – connected to what?
4. Lines 116-119: This sentence is not clear. Is the hypothesis that smaller organisms likely to disperse further because they disperse passively, or simply because they are more abundant?
5. Line 128-129: Do you mean here that “β-diversity has been found to be mainly controlled by surface ocean transit time” in this study or elsewhere? The use of past tense is confusing, present tense should be used for the study being described in this manuscript.
6. Line 170: Remove ‘not surprisingly’.
7. Response to review comment 1.2: This (cell division rates being more important for dispersal than growth rates in phytoplankton) seems an important point which could be incorporated in the discussion?
8. The manuscript needs editing for grammar, e.g.: Line 63: Suggest ‘compared to more abundant small-bodied plankton’, making it clear that the 2 refer to the same group of organisms. Line 69-

70: Remove commas or split sentence. Line 72-73: Put the definition of ‘dispersal’ in brackets to make it clear it is a definition and reduce use of commas in this sentence. Line 85: Remove unnecessary word ‘Further’. Line 191: re-structure sentence.

Second round of response to the reviewer's comments

Manuscript No.: NCOMMS-16-24484 entitled "***Large scale ocean connectivity and planktonic body size***"

- RC: Reviewer comment (in italic and grey)
- Author Response (AR) in blue

Reviewer #1

- ### RC 1.1:

The manuscript overall improved considerably compared to the previous submission. However, the language of the text needs to be checked carefully, there are many long sentences and syntax errors (not commented below).

AR: We appreciate the reviewer comment about the manuscript improvement. We have make sentences shorter and we have amended for the syntax errors in the new version of the manuscript, which has been carefully revised by co-author James Watson.

- ### RC 1.2:

My only major concern is that the authors fail to address a conflicting finding of the study, namely finding on the one hand weaker distance decay in microbes and protists, while they could not show a stronger (local) environmental control in these groups (as should be expected, following hypothesis 1). This can be interpreted that some important env parameters are missing. More specifically, the smallest groups exhibit pronounced short-term (seasonal) fluctuations in their community composition, which might not be represented well in the available env data. It may, hoever, also point at limitations regarding the quantification of connectivity, as transit times (e.g. roles of considering regional population sizes are not considered).

AR: There is probably a misunderstanding here. A weaker decay in similarity with distance means that microbe and protist communities in point A and B are more similar than for example larger sized zooplankton communities. Results have provided evidence that this is in large part due to currents derived dispersal limited processes, independently of the environmental conditions (Table 1). In fact, the correlation between the surface ocean transit times and the environmental distance has been found to be rather weak among the all pair-sites (Mantel r = 0.09) (see also a detailed explanation in RC 1.12). To cover this, we have included the following sentence in the new version of the manuscript (see lines 146-148) "The correlation between the surface ocean transit times and the environmental distance among the all pair-sites is rather weak (Mantel r = 0.09, Supplementary Table 1A)".

Referring to what the manuscript says about hypothesis 1 (lines 91-93): "(1*) local niche-based processes, which is epitomized by the hypothesis that, below 1-mm body size, "everything is everywhere, but the environment selects*". That is, "environment selects", but we don't say whether we expect "a stronger or weaker (local) environmental control" for these microbes and protist groups. That said, the reviewer is right in that other environmental data not measured in this study might play a role in controlling community

composition at all taxonomic levels because communities are shaped by historical processes and instantaneous measures of environmental variables such as temperature, salinity, O2.... However, the environmental gradients employed in the analysis (sea surface temperature values range from 14.7º-29.6ºC; sea surface salinity = 33-37; Photosynthetic Active Radiation= 0-1200 Wm^{-2}) should be large enough to have an effect in the plankton community assembly. Following the reviewer suggestion, we have included the following sentence in the new version of the manuscript (see lines 253-255) "Other environmental data not measured in this study might also play a role in controlling community composition at all taxonomic levels".

We do agree that small groups typically show pronounced variation in temporal patterns of community assembly in a particular location, as has been already well reported for marine bacteria[1,2], zooplankton[3], phytoplankton[4] and protist[5]. However, our paper does not focus on plankton beta-diversity patterns in time but in space. On the other hand, the view that local sorting should be the strongest in smaller organisms as the reviewer points out is an open question and some studies, e.g. Farjalla et al[6], found that habitat filtering increase with body size arguing that larger taxa often exhibit lower plasticity in the fundamental niches and have stronger competitive interactions[6]. We think that seasonal variation of local communities should not have a relevant effect on the relations found between community similarity and oceanic transit times.

- **RC 1.3:**

Abundances are invoked as a 'mechanism' supporting higher connectivity in small(=abundant) organisms, yet the mechanism isnt specified (the effectve population size should consider biovolume, not cellular abundance). Possession of resting stages (in microbes & protists) & low sinking losses seems to be a more valid argument than cellular abundance.

AR: We appreciate this insightful comment done by the reviewer. We agree in that low sinking losses, which highly depend on the cell size[7,8], and the long term persistence as resting stages of not only bacteria and protist[9] but also phytoplankton[10] are two important mechanism playing a role in the degree to which planktonic organism disperse. In fact, slow sinking cells stay more time in the water column so they are prone to higher advection compared to fast sinking larger organisms, which makes smaller organisms to disperse farther. Possession of resting stages in small sized plankton also make these organisms to spend more time dwelling in the water column, which results in longer dispersal distances. However, nor sinking losses nor dormant stages have been measured during the Malaspina survey, so we cannot conclude if they are a more important mechanism than abundance explaining dispersal patterns of plankton. In our opinion, the three mechanisms (high abundance, low sinking rates and the presence of resting stages) might be complementary triggering smaller planktonic organisms to disperse farther, compared to bigger sized plankton. In the introduction lines (110-120), we explain the relationship between the abundance and dispersal patterns of passively dispersing groups and the mechanism behind. We have included a sentence in the discussion in order to recall the reader on that (see lines 236-238) "Locally abundant species are exposed to lower local extinction rates[11] and hence, reduced demographic stochasticity and ecological drift[12]. Therefore, we suggest that large population densities and short generation times of micro-planktonic organisms are the mechanisms explaining the larger geographic range and relatively weak spatial structure of these organisms[11,13-15]". We have remarked the point about sinking losses and we have add around those lines a note on

the potential effect of resting stages following the point raised by the reviewer (see lines 244-246) "In addition, lower sinking losses[8] and longer survival times of resting stages of small passively-dispersed plankton (from prokaryotes to phytoplankton)[9] allow their populations to travel farthest compared to large-sized plankton".

Specific comments

- **RC 1.4:**

L 53 "we quantified the dispersal scale and community structure – β-diversity" – community structure is not beta diversity (insert 'spatial' before community structure)

AR: Agreed, done.

- **RC 1.5:**

l. 71-74 – split sentence (structure)

AR: Agreed, done. Now it reads as (see lines 73-75) "However, biological connectivity, or the exchange of individuals among geographically separated subpopulations[16], is not uniform as there exist barriers to dispersal, i.e. movement of individuals across space[16]. Such barriers include land masses, frontal systems, gyres, and other oceanographic features[17]".

- **RC 1.6:**

l. 76-77 – why differences in _expected_ body size?

AR: No reason for including "expected". Deleted.

I'm missing a consideration that you address passively dispersing organisms in this paragraph. Reg comp with terr habitats (which are spatially structured), I doubt you can readily derive expectations for organisms drifting in a continuum, unless you address explicitly passive dispersers

AR: The aim of comparing passively dispersed marine organism vs terrestrial ones is to frame our study in a more general ecological hypothesis, and show that terrestrial research in body-size dependent spatial patterns of diversity is one step ahead compared to marine habitats. Similarities between terrestrial fragmented landscapes and marine "continuum" do exist: the ocean has also discontinuities that limit dispersal, as for example terrestrial barriers, frontal systems, gyres or other oceanographic features[17]. In the former version of the paper we included examples of body-size influence in shaping community structure and dispersal scales of actively and passively dispersing terrestrial animals[18,19]. According to the reviewer, we have included in the new version a citation of passively dispersers[20], and we keep the actively and passively dispersing example of the insects, a sound work of Siemann and colleagues[19] (see lines 78-81).

- **RC 1.7:**

l. 98f – overall, there is a surely correlation between spatial and env distance, but this may have various shapes - not necessarily proportional (would require to be linear on log-log scale)

AR: Agreed. We have changed the word "proportional" by "correlated".

- **RC 1.8:**

l. 103 f – there is surely a robust (=general) distance decay pattern across organisms (see reviews by Hillebrand and others), you rather mean that slopes differ across phylogenetic groups(?)

AR: With this sentence, what we want to say is that distance-decay slopes, which are a function of the distance-decay relationships, differ among taxa due to differing body size in aquatic and terrestrial domains. We are quite in line with what Soininen 2007[21] reported in their work "*Despite the recent attention to the distance decay relationship, there is no consensus on how the relationship varies across organism groups, geographic gradients and environments*". To include the Soininen 2007[21] work and following the reviewer suggestion, we have changed the sentence into (see lines 100-105): ". Indeed, while distance-decay patterns have been observed for specific taxa in terrestrial (e.g. rainforest trees[22]), freshwater (e.g. aquatic beetles[23]; fish and macroinvertebrates[24]), and marine communities (e.g. coral reefs[12]; marine bacteria and prokaryotes[25,26]; and macrobenthos and plankton[27]), few studies have identified a robust distance-decay pattern across-taxa or across key physiological traits such as body size[21]".

- **RC 1.9:**

l 108 – metabolic rates scale with growth rates, small organisms have _high_ rates

AR: Agreed and changed (see line 107).

- **RC 1.10:**

l 110. – abundant in terms of cellular/organismal density or biomass? Quite a difference when comparing e.g. viruses with phytoplankton and bacteria

AR: We meant abundance in terms of population density. In order to clarify that, we have included the following sentence in the new version of the manuscript (see lines 110-112): "Other implications of body size are that small organisms are generally more abundant, in terms of population density, than larger organisms[28]".

- **RC 1.11:**

l. 117 ff – "Further, smaller more abundant organisms are likely to disperse further with oceanic currents" – why? The extinction argument was made before, what else would support farther dispersal (among passive dispersers)

AR: There is a small typo here that makes the sentence confusing. We meant "farther" instead of "further". Now it reads as (see lines 117-120) "In the oceans, it is therefore expected that smaller planktonic organisms, which are relatively more abundant, to

disperse farther with oceanic currents[29], leading to shallower distance-decay slopes when compared to those of larger planktonic organisms[26,30-32]".

- **RC 1.12:**

l. 146 – finding a low correlation between spat and env distance is in contrast to expectations and should be highlighted more

AR: At regional scales, the differences in environmental characteristics highly correlate to the geographic distance[12,31,33]. However, at the scale of the study (tropical and subtropical regions of the world's oceans), we found a low correlation between the ocean transit times and the environmental distance (Mantel r = 0.09, now included see lines 146-148). This might be because a sampling point in the northern tropical hemisphere, say 30º north in latitude, is climatically very similar to a sampling point at -30º, despite being geographically far apart. At regional scales within the same Hemisphere and ocean, we could had found higher correlations between pair-sites. We have included the following sentence in the discussion (see lines 208-213) "The low spatial autocorrelation between oceanic transit time and environmental distance found is due to the global scale of the study (tropical and subtropical regions of the world's oceans). Contrary to most regional studies where climate and space correlates well, here climatically very similar locations can be geographically far apart (for instance, two antipode points in the equator or two points at 30º North and South)".

- **RC 1.13:**

l. 171 – importance of distance per se depends on quality of env data

AR: The reviewer might refer to the fact that if more environmental drivers are included, the environmental distance of the study would had been more precise (see response 1.2).

- **RC 1.14:**

l. 246 – linking dispersal to generation time is acceptable as mechanism – but what is the mechanism underlying the abundance argument?

AR: The mechanism stated in the introduction (see lines 107-120) now has been rewritten and completed and also included for clarification in the discussion too (see lines 236-243) "Locally abundant species are exposed to lower local extinction rates[11] and hence, reduced demographic stochasticity and ecological drift[12]. Therefore, we suggest that large population densities and short generation times of micro-planktonic organisms are the mechanisms explaining the larger geographic range and relatively weak spatial structure of these organisms[11,13-15]. In contrast, larger planktonic organisms have in general longer generation times and smaller population densities[34], and therefore they are more sensitive to local extinctions and ecological drift, resulting in stronger spatial structure"

- **RC 1.15:**

L 297 – this paragraph isn't very clear (high amount of shared taxa, simultaneously high beta div) –"sample sites between subtropical gyres of the Atlantic, Pacific and Indian

Oceans are extremely well connected in terms of species shared (i.e. acting as bridges between ocean provinces) with relatively high β-diversity" high beta diversity are reported together with high connectivity"

AR: We do agree with the reviewer that the sentence, as it stands, it is a bit confusing, particularly because if a sampling station is extremely well connected to the others, in terms of shared species, the relative beta-diversity of that station will be low, and not high, as it was written before. We have changed the sentences to cover the reviewer points. Now, the results section reads as (see lines 191-193) "Network graphs also reveal an area of high β-diversity for myctophids in the central Pacific Ocean (Fig. 4C, pink points), where species connectivity is low due to limited mixing between neighboring communities" and in discussion as (see lines 286-292) "In particular, we identified large-scale frontal zones as areas of low β-diversity in the case of meso-zooplankton and specially myctophids fishes. These frontal zones act as barriers separating subtropical gyres, and are typically areas of relatively high primary production[35]. Limited dispersal between distinct pelagic provinces has been shown to play a major role in plankton population differentiation, and the creation of strong genetic breaks and enhanced diversity in bridging region[36]"

Reviewer #2

- **RC 2.1:**

Though I really appreciated the novelty of using oceanic micro plastics in some form of null inquiry in the previous version, I agree with your decision to remove this from the current manuscript. I think it is considerably better now, though there are still a number of proofreading errors and so please check it again with a fine-toothed comb! Otherwise I feel that my concerns have been addressed, this is a nice contribution.

AR: We appreciated the nice words from the reviewer. On the new version of the manuscript, syntax errors and long sentences have been avoided, after a careful revision of co-author James Watson.

Reviewer #3

- **RC 3.1:**

The manuscript is now greatly improved. I appreciate that the authors have clarified their terminology, restructured the manuscript and provided more methodological detail. I also agree with the decision to remove microplastics from the analysis. The manuscript requires minor edits for further clarity (detailed below) and grammatical issues. Note that I am unable to comment on the appropriateness of the biological and statistical content of the paper, which I leave for other reviewers.

AR: We appreciated the reviewer comments on the manuscript improvement. Grammatical issues have been carefully addressed by co-author James Watson.

- **RC 3.2:**

ECCO2 should be described as a global ocean circulation model as opposed to simply a global model. Secondly, I would not describe 1/4° as 'high resolution', for example when compared to the HYCOM global reanalysis circulation output which is provided at 1/12° 3-

hourly or daily resolution on a global scale for the period 1992-present. The vertical resolution of the ECCO2 fields is also low if velocities are given as an average between 5 and 20m depth.

AR: We have changed the wording to a global Ocean General Circulation Model (OGCM). We have also changed the text to reflect that the horizontal model resolution is eddy permitting (see lines 474-479). "In brief, velocity fields from the ECCO2 model (http://ecco2.org), an eddy permitting global Ocean General Circulation Model (OGCM) with a 1/4ºx1/4º horizontal resolution that assimilates satellite and in-situ data using a 4D-var approach, were used as inputs to the TRACMASS offline particle tracking framework[37] to advect virtual particles, bound to the near surface (5-20 m depth), using only horizontal velocities". The effect of model resolution on the connectivity matrix is described in detail in Jonsson & Watson 2016[38]. In short, we assume that models with higher resolutions will result in more explicit pathways as inputs for the shortest pathway analysis and hence potentially shorter min-t connectivity times (ocean transit times). At the same time, the fact that ECCO2 provide a physically consistent state estimate using 4D-VAR minimizes the risk for unrealistic pathways due to the data assimilation in for example HYCOM. As an effect, we believe that the use of ECCO2 as source for surface velocities is a reasonable and conservative choice for the task at hand. It should also be noted that we don't use mean values between 5 and 20 meters depth, but the second layer in the model. This is to avoid interference with air-sea exchange in the model and makes our virtual drifters to behave in a similar fashion to floats in the Global Drifter Program (http://www.aoml.noaa.gov/phod/dac/index.php) (see lines 471 – 489).

- **RC 3.3:**

I still feel more detail could be added on the modelling approach; What dispersal modelling system did you use? (n.b. a comma should be added in line 488 before 'advect' to make it clear that ECCO2 does not do the particle advection itself but is instead used as input data). What is the temporal resolution of the ECCO2 velocity fields (which is as, if not more, important in influencing modelled patterns of dispersal than spatial resolution)? How frequently did you 'seed' particles? Presumably the model was run in 2D and particles weren't able to move in the vertical? Even if you don't feel this detail to be important in the methods, as it refers to a previous study, these considerations should be still be discussed as potential limitations of the underlying modelled surface ocean transit times. Finally, you do not cite Jonsson and Watson 2016 until the last line, where it looks instead like it refers to the depth layer of the ECCO2 output.

AR: We use the TRACMASS off-line particle tracking framework[37] where individual particles are advected by interpolating velocity fields in space and time. This is a robust approach that has been used in nearly hundred studies over several decades. We do not add extra dispersal to the particles since we want the result to be as conservative as possible. The ECCO2 model has a horizontal resolution of 1/4° x 1/4° degree and assimilates observations using 4D-Var to create an internally consistent state estimate of the global ocean. We have added more information about the model and connectivity matrix to the text (see lines 471-489).

- **RC 3.4:**

The new paragraph at lines 183-193 seems a bit out of place – I'm not sure what you are trying to say here and why this is important to the results, especially as it refers to

previously published results. Perhaps it could be shortened/simplified and merged with the following paragraph? Further, I'm not sure how you conclude from this figure that the Atlantic is 'less connected' than other basins – connected to what?

AR: We have revised and shortened the paragraph and we have move it to methods (see lines 491-498), because the reviewer is right in that Figure 5 is not a result of this paper. We think it is important to include this figure because it not only shows the variability of the surface ocean transit time of the world's oceans, which is a key component of our analysis (transit-time matrix), but also the myctophids and macro-zooplankton unique cluster arrangements correspond well with the less connected areas of the world oceans (see discussion lines 292-298). By saying that the Atlantic is less connected we mean that the minimum connection times between the Atlantic and the other ocean are longer compared to the connection times of the other oceans. This has been clarified in the text (see lines 496-498).

- **RC 3.5:**

Lines 116-119: This sentence is not clear. Is the hypothesis that smaller organisms likely to disperse further because they disperse passively, or simply because they are more abundant?

AR: We agree with the reviewer that the sentence can make confusion. In order to clarify that, now it reads as (See lines 117-120) "In the oceans, it is therefore expected that smaller planktonic organisms, which are relatively more abundant, to disperse farther with oceanic currents[29], leading to shallower distance-decay slopes when compared to those of larger planktonic organisms[26,30-32]".

- **RC 3.6:**

Line 128-129: Do you mean here that "β-diversity has been found to be mainly controlled by surface ocean transit time" in this study or elsewhere? The use of past tense is confusing, present tense should be used for the study being described in this manuscript.

AR: Agreed. We have included the present sentence whenever we have referred to our study.

- **RC 3.7:**

Line 170: Remove 'not surprisingly'.

AR: Agreed. Done

- **RC 3.8:**

Response to review comment 1.2: This (cell division rates being more important for dispersal than growth rates in phytoplankton) seems an important point which could be incorporated in the discussion?

AR: This is true for phytoplankton. However, we think that incorporating this into the discussion can be confusing because our paper analyzes several groups, from

prokaryotes to small fishes, not only phytoplankton, where size and growth rates are tightly linked[39,40].

- **RC 3.9:**

The manuscript needs editing for grammar

- *e.g.: Line 63: Suggest 'compared to more abundant small-bodied plankton', making it clear that the 2 refer to the same group of organisms.*

  AR: Agreed. Done.

- *Line 69-70: Remove commas or split sentence.*

  AR: Agreed. Done. We have split the sentence which now reads as (see lines 71-72) "The oceans can be considered the largest continuous environment on Earth. Over long timescales, all marine ecosystems are connected to each other by ocean currents[38]".

- *Line 72-73: Put the definition of 'dispersal' in brackets to make it clear it is a definition and reduce use of commas in this sentence.*

  AR: Agreed. Done.

- *Line 85: Remove unnecessary word 'Further'. Line 191: re-structure sentence.*

  AR: Agreed. Done.

References

1 Hatosy, S. M. *et al.* Beta diversity of marine bacteria depends on temporal scale. *Ecology* **94**, 1898-1904, doi:10.1890/12-2125.1 (2013).

2 Langenheder, S., Berga, M., Ostman, O. & Szekely, A. J. Temporal variation of [beta]-diversity and assembly mechanisms in a bacterial metacommunity. *ISME J* **6**, 1107-1114, doi:http://www.nature.com/ismej/journal/v6/n6/suppinfo/ismej2011177s1.html (2012).

3 Chain, F. J. J., Brown, E. A., MacIsaac, H. J. & Cristescu, M. E. Metabarcoding reveals strong spatial structure and temporal turnover of zooplankton communities among marine and freshwater ports. *Diversity and Distributions* **22**, 493-504, doi:10.1111/ddi.12427 (2016).

4 Mousing, E. A., Richardson, K., Bendtsen, J., Cetinić, I. & Perry, M. J. Evidence of small-scale spatial structuring of phytoplankton alpha- and beta-diversity in the open ocean. *Journal of Ecology* **104**, 1682-1695, doi:10.1111/1365-2745.12634 (2016).

5 Piredda, R. *et al.* Diversity and temporal patterns of planktonic protist assemblages at a Mediterranean Long Term Ecological Research site. *FEMS microbiology ecology* **93**, fiw200-fiw200, doi:10.1093/femsec/fiw200 (2017).

6 Farjalla, V. F. *et al.* Ecological determinism increases with organism size. *Ecology* **93**, 1752-1759, doi:10.1890/11-1144.1 (2012).

7 Bienfang, P. K. Phytoplankton sinking rates in oligotrophic waters off Hawaii, USA. *Marine Biology* **61**, 69-77, doi:10.1007/bf00410342 (1980).

8 Finkel, Z. V. *et al.* Phytoplankton in a changing world: cell size and elemental stoichiometry. *Journal of Plankton Research* **32**, 119-137, doi:10.1093/plankt/fbp098 (2010).

9 Lennon, J. T. & Jones, S. E. Microbial seed banks: the ecological and evolutionary implications of dormancy. *Nat Rev Micro* **9**, 119-130, doi:http://www.nature.com/nrmicro/journal/v9/n2/suppinfo/nrmicro2504_S1.html (2011).

10 Ellegaard, M. & Ribeiro, S. The long-term persistence of phytoplankton resting stages in aquatic 'seed banks'. *Biological Reviews*, n/a-n/a, doi:10.1111/brv.12338 (2017).

11 Pimm, S. L., Jones, H. L. & Diamond, J. On the Risk of Extinction. *The American Naturalist* **132**, 757-785 (1988).

12 Hubbell, S. P. *The Unified Neutral Theory of Biodiversity and Biogeography (MPB-32)*. (Princeton University Press, 2001).

13 Blackburn, T. M. & Gaston, K. J. *Macroecology: Concepts and Consequences: 43rd Symposium of the British Ecological Society*. (Cambridge University Press, 2003).

14 MacArthur, R. H. & Wilson, E. O. *The Theory of Island Biogeography*. (Princeton University Press, 1967).

15 Whitfield, J. Is Everything Everywhere? *Science* **310**, 960 (2005).

16 Cowen, R. K., Gawarkiewicz, G., Pineda, J., Thorrold, S. R. & Werner, F. E. Population Connectivity in Marine Systems. *Oceanography* **20** (2007).

17 Longhurst, A. R. in *Ecological Geography of the Sea (Second Edition)* 51-70 (Academic Press, 2007).

18 Borthagaray, A. I., Arim, M. & Marquet, P. A. Connecting landscape structure and patterns in body size distributions. *Oikos* **121**, 697-710, doi:10.1111/j.1600-0706.2011.19548.x (2012).
19 Siemann, E., Tilman, D. & Haarstad, J. Insect species diversity, abundance and body size relationships. *Nature* **380**, 704-706 (1996).
20 Chu, C.-J. *et al.* On the Balance between Niche and Neutral Processes as Drivers of Community Structure along a Successional Gradient: Insights from Alpine and Sub-alpine Meadow Communities. *Annals of Botany* **100**, 807-812, doi:10.1093/aob/mcm166 (2007).
21 Soininen, J., Lennon, J. J. & Hillebrand, H. A multivariate analysis of beta diversity across organism and environments. *Ecology* **88**, 2830-2838, doi:10.1890/06-1730.1 (2007).
22 Condit, R. *et al.* Beta-Diversity in Tropical Forest Trees. *Science* **295**, 666 (2002).
23 Baselga, A. *et al.* Whole-community DNA barcoding reveals a spatio-temporal continuum of biodiversity at species and genetic levels. *Nat Commun* **4**, 1892, doi:10.1038/ncomms2881 (2013).
24 Shurin, J. B., Cottenie, K. & Hillebrand, H. Spatial Autocorrelation and Dispersal Limitation in Freshwater Organisms. *Oecologia* **159**, 151-159 (2009).
25 Martiny, J. B. H., Eisen, J. A., Penn, K., Allison, S. D. & Horner-Devine, M. C. Drivers of bacterial β-diversity depend on spatial scale. *Proceedings of the National Academy of Sciences* **108**, 7850-7854, doi:10.1073/pnas.1016308108 (2011).
26 Salazar, G. *et al.* Global diversity and biogeography of deep-sea pelagic prokaryotes. *ISME J*, doi:10.1038/ismej.2015.137 (2015).
27 Chust, G. *et al.* Dispersal similarly shapes both population genetics and community patterns in the marine realm. *Scientific Reports* **6**, 28730 (2016).
28 Peters, R. H. *The Ecological Implications of Body Size*. (Cambridge University Press, 1986).
29 Finlay, B. J., Esteban, G. F. & Fenchel, T. Global diversity and body size. *Nature* **383**, 132-133 (1996).
30 Finlay, B. J. Global Dispersal of Free-Living Microbial Eukaryote Species. *Science* **296**, 1061-1063, doi:10.1126/science.1070710 (2002).
31 Martiny, J. B. H. *et al.* Microbial biogeography: putting microorganisms on the map. *Nat Rev Micro* **4**, 102-112 (2006).
32 De Wit, R. & Bouvier, T. 'Everything is everywhere, but, the environment selects'; what did Baas Becking and Beijerinck really say? *Environmental Microbiology* **8**, 755-758, doi:10.1111/j.1462-2920.2006.01017.x (2006).
33 Telford, R. J., Vandvik, V. & Birks, H. J. B. Dispersal Limitations Matter for Microbial Morphospecies. *Science* **312**, 1015 (2006).
34 Sheldon, R. W., Prakash, A. & Sutcliffe, W. H. The size distribution of particles in the ocean. *Limnology and Oceanography* **17**, 327-340, doi:10.4319/lo.1972.17.3.0327 (1972).
35 Cermeño, P. *et al.* The role of nutricline depth in regulating the ocean carbon cycle. *Proceedings of the National Academy of Sciences* **105**, 20344-20349, doi:10.1073/pnas.0811302106 (2008).
36 Goetze, E. Population differentiation in the open sea: insights from the pelagic copepod Pleuromamma xiphias. *Integrative and comparative biology* **51**, 580-597, doi:10.1093/icb/icr104 (2011).

37 Döös, K. Interocean exchange of water masses. *Journal of Geophysical Research: Oceans* **100**, 13499-13514, doi:10.1029/95JC00337 (1995).
38 Jonsson, B. F. & Watson, J. R. The timescales of global surface-ocean connectivity. *Nat Commun* **7**, doi:10.1038/ncomms11239 (2016).
39 Rinaldo, A., Maritan, A., Cavender-Bares, K. K. & Chisholm, S. W. Cross-scale ecological dynamics and microbial size spectra in marine ecosystems. *Proceedings of the Royal Society B: Biological Sciences* **269**, 2051-2059, doi:10.1098/rspb.2002.2102 (2002).
40 Kiørboe, T. & Hirst, A. G. Shifts in Mass Scaling of Respiration, Feeding, and Growth Rates across Life-Form Transitions in Marine Pelagic Organisms. *The American Naturalist* **183**, E118-E130, doi:doi:10.1086/675241 (2014).

Reviewers' comments:

Reviewer #1 (Remarks to the Author):

The authors have addressed most of my concerns successfully. I see one remaining issue, which should be addressed more clearly in the text. It relates to the fact that 'microbes' show simultaneously weak distance decay, but also a weak match with env gradients. I'm not askin for new analyses, but to rethnik the arguments how we can explain both weak env match and weak distance decay within one group.
The argumentation of teh authors is somewhat opportunistic. Weak distance decay is attributed to high dispersal. However, at teh same time the authors emphasize short generationt times and high metabolic rates in tehse groups. Phytoplankton is traditionally being used as excellent indicator for water quality due to the fact that these communities respond almost instantaneously to env change. The underlying point is that communities should reflect the result of species sorting. And this process should be assumed to be highly efficient in organisms that dipserse easily and grow fast (same for their decay). So in short, the authors should think a bit about laws of species sorting & community assembly along their allometric gradient - the mechanisms they have outlined should advocate for best match between env and community composition in the smalles organisms. (mass effects are out of question for the transit times that were reported).

Reviewer #3 (Remarks to the Author):

I consider the content of the manuscript ready for publication. However, there are still frequent grammatical errors which affect readability. Some examples are given below. I will leave these corrections to the editor to resolve and do not need to see a revised version of the manuscript.
Present and past tense are again used interchangeably in the abstract (lines 59 and 64) – "have shown" (line 64) suggests the work of previous studies.
Line 71-72: Suggest "over *sufficiently* long timescales".
Line 74-5: There needs to be some punctuation here to separate out the definition of dispersal, or remove the definition given in quotation marks.
Line 111: Organism*s*.
Line 118-119: Grammatical error, replace 'to' with 'will'.
Line 138: Remove the word 'shaping'.
Line 142: Single or multiple correlations? Should read either "The correlation with" or "Correlations with".
Line 152: Mechanism*s*.
Line 206: Either "time explains" or "times explain".
Line 223: Remove the world 'their'.
Line 231: Remove the world 'its'.
Line 277-279: Insert: "However, *the study of* Jenkins…" and remove the word 'it'.
Line 481: Remove the word 'model' (unnecessary when the more accurate word 'simulation' is used). This sentence is also very long.

Third round of response to the reviewer's comments

Manuscript No.: NCOMMS-16-24484 entitled "***Large scale ocean connectivity and planktonic body size***"

- Reviewer comment (in italic and grey)
- Author Response (AR) in blue

Reviewer #1

"The authors have addressed most of my concerns successfully. I see one remaining issue, which should be addressed more clearly in the text. It relates to the fact that 'microbes' show simultaneously weak distance decay, but also a weak match with env gradients. I'm not asking for new analyses, but to rethink the arguments how we can explain both weak env match and weak distance decay within one group. The argumentation of the authors is somewhat opportunistic. Weak distance decay is attributed to high dispersal. However, at the same time the authors emphasize short generation times and high metabolic rates in these groups. Phytoplankton is traditionally being used as excellent indicator for water quality due to the fact that these communities respond almost instantaneously to env change. The underlying point is that communities should reflect the result of species sorting. And this process should be assumed to be highly efficient in organisms that disperse easily and grow fast (same for their decay). So in short, the authors should think a bit about laws of species sorting & community assembly along their allometric gradient - the mechanisms they have outlined should advocate for best match between env and community composition in the smallest organisms. (mass effects are out of question for the transit times that were reported).

AR: We understand reviewer#1's concerns about microbes showing both weak distance-decay and environmental match, because microbes often respond fast to environmental change, and perceive the environment at relatively very fine spatial and temporal scales[1,2]. In our case, different reasons might explain why we found weak environmental match in microbes, and in the large-sized plankton groups. The first reason is that most of the Malaspina cruise was restricted to tropical and subtropical regions and took place during the summer stratification period. Under these conditions, the vertical gradient in environmental variables is much stronger than the horizontal one (see examples of phytoplankton[3]). Our data were either from surface or vertically integrated (0-200 m), and therefore vertical variability is missed. On the other hand, except for a few regions (e.g. Equatorial Upwelling), the horizontal variability of the environmental variables we considered was not strong enough to capture strong environmental species sorting of the microbial and plankton communities, given their degree of niche plasticity. The situation could be different if we had samples along a gradient of an upwelling event, a pulse of vertical mixing, across coastal to offshore, or stronger temperature gradients; situations in which a stronger environmental control would be expected. Our study also excluded biotic interactions, which possibly play a strong role in driving spatial distribution patterns, particularly with large planktonic taxa. Note, in this context, the portion of unexplained variance in microbes approximately 70%, Table 1.

Second, marine microbial communities are dispersed mainly by currents (see Table 1), implying that their spatial distributions should be ubiquitous. As a fundamental driver of realized dispersal is high absolute abundance, and as organism size and abundance are inversely related, this also leads to microbes with ubiquitous spatial distributions[4,5]. This is why we observe relatively weak decay-patterns and high dispersal in smaller organisms, when compared to larger organisms. The reviewer is right that phytoplankton, together with other autotrophic organisms should show higher environmental control, as they need light and nutrients for their production[5]. In fact, environmental species sorting has been shown to be an important determinant for the assembly of marine diatom communities[6]. However, as explained above, the environmental conditions in the horizontal were relatively homogeneous during our sampling.

The third reason is that a stronger relationship between microbes and plankton with environmental factors would be better observed in the relative abundance of species composition, instead of the presence-absence index we used, which is less sensitive to environmental gradients. In fact, a recent meta-analysis by Soininen[7] concluded that studies using abundance data showed a higher degree of environmental species sorting, which is related to the assemblage variation explained by the environment[8], compared to studies based on presence-absence data. In our study, each group's abundance was converted into presence-absence to determine distance-decay relationships based on Jaccard dissimilarity index. We did so because in some groups (prokaryotes, microbial eukaryotes, meso-zooplankton 0-200 m and macro-zooplankton) abundances were estimated using genetic techniques, which are not precise enough to infer trustable abundances.

In line with the reviewer's comment, there is a vast literature reporting the use of phytoplankton, and particularly diatoms, as water-quality indicators[9]. Nonetheless, these indicators are mainly based on overall biomass and specific species linked to their biological traits. The reviewer is right in that small groups typically show pronounced variation in temporal patterns of community assembly in a particular location, as it has been already well reported for marine bacteria[10,11], zooplankton[12], phytoplankton[13] and protists[14]. However, our study does not focus on plankton beta-diversity patterns in time but in space. A community monitored over time, for example time-series studies, could be better examined for any relationship with changes in environmental factors. It is worth noting that weak relationships between species similarity and environmental factors have also been reported in similar studies dealing with spatial patterns of phytoplankton beta-diversity[15].

In our study, the body size to environmental species sorting relationship, based on a gradient spanning 6 orders of magnitude in body size, is rather weak, and this is in line with a recent meta-review by Soininen[7], which aimed to determine the variation in the degree of species sorting along allometric gradients and trophic levels (including datasets of up to 12 order of magnitude in body-size). This suggest that the strength of environmental species sorting among size-varying organisms needs to be further investigated, and research efforts should also focus on integrating biotic interactions on meta-community studies, particularly when dealing with larger taxa.

Aiming to address what the reviewer points out, in the new version of the manuscript we have changed and expanded the paragraph that deals with the weak relationship between environmental filtering and body-size. Now it reads (See lines 255-275):

"In our study, the environment, through environmental species sorting, explains little of the observed spatial variation in community structure in both the plankton and micronekton groups. Different reasons might explain this. First, the Malaspina sampling was restricted to tropical and subtropical regions and it took place in summertime where horizontal environmental gradients are typically low at surface, and hence it is difficult to capture assemblage variations due to climate. Second, the presence-absence indices that we used are less sensitive[7], relative to relative abundances, which we anticipate would potential identify a stronger relationship in both small and large sized plankton and micro-nekton with environmental gradients. Other potential reasons might be on the other environmental variables not measured in our study, and the exclusion of biotic variables, which might play a role driving spatial distribution, particularly in large planktonic taxa. Finally, marine microbial communities are mainly dispersed by advection and diffusion, and together with their high niche plasticity when compared to larger-bodied taxa, results in that their spatial distribution should be relatively broad. However, our results do not identify low niche plasticity in large-bodied taxa[16], and further we observe no significant relationship between organism body-size and environmental variability. This is in line with a recent meta-analysis by Soininen[7], that concluded that body-size and environmental species sorting are not significantly related in a dataset spanning a range in body-size of up to 12 orders of magnitude. This apparent contradiction in thinking and evidence highlights the need for further research on the strength of environmental species sorting among organisms of different size".

Reviewer #3

"I consider the content of the manuscript ready for publication". However, there are still frequent grammatical errors which affect readability. Some examples are given below. I will leave these corrections to the editor to resolve and do not need to see a revised version of the manuscript".

AR: We appreciate a lot the reviewer words supporting manuscript publication.

Specific comments on language issues

- *Present and past tense are again used interchangeably in the abstract (lines 59 and 64) – "have shown" (line 64) suggests the work of previous studies.*
  AR: Agreed. Changed.
- *Line 71-72: Suggest "over *sufficiently* long timescales".*
  AR: We prefer it as it is.
- *Line 74-5: There needs to be some punctuation here to separate out the definition of dispersal, or remove the definition given in quotation marks.*
  AR: Agreed. Removed.
- *Line 111: Organism*s*.* AR: Agreed. Done
- *Line 118-119: Grammatical error, replace 'to' with 'will'.*
  AR: It does not make sense to replace it by "will". It is a comparison between two things "compared…to".

- *Line 138: Remove the word ‘shaping’.*
  AR: Agreed. Done
- *Line 142: Single or multiple correlations? Should read either “The correlation with” or “Correlations with”.*
  AR: Agreed. Done
- *Line 152: Mechanism*s*.*
  AR: Agreed. Done
- *Line 206: Either “time explains” or “times explain”.*
  AR: Agreed. Done
- *Line 223: Remove the world ‘their’.*
  AR: Agreed. Done
- *Line 231: Remove the world ‘its’.*
  AR: We don’t agree. It helps to refer to large organism similarity.
- *Line 277-279: Insert: “However, *the study of* Jenkins…” and remove the word ‘it’.*
  AR: Agreed. Done
- *Line 481: Remove the word ‘model’ (unnecessary when the more accurate word ‘simulation’ is used). This sentence is also very long.*
  AR: Agreed. Now it reads as (see lines 471-473) “Estimates of minimum connection time or surface ocean transit time between pair-sites were obtained from a previously published global surface ocean Lagrangian particle simulation[17]”.

References

1 Azovsky, A. I. Size-dependent species-area relationships in benthos: is the world more diverse for microbes? *Ecography* **25**, 273-282, doi:10.1034/j.1600-0587.2002.250303.x (2002).

2 Allison, S. D. & Martiny, J. B. H. Resistance, resilience, and redundancy in microbial communities. *Proceedings of the National Academy of Sciences of the United States of America* **105**, 11512-11519, doi:10.1073/pnas.0801925105 (2008).

3 Estrada, M. *et al.* Phytoplankton across Tropical and Subtropical Regions of the Atlantic, Indian and Pacific Oceans. *PLoS ONE* **11**, e0151699, doi:10.1371/journal.pone.0151699 (2016).

4 Finlay, B. J. Global Dispersal of Free-Living Microbial Eukaryote Species. *Science* **296**, 1061-1063, doi:10.1126/science.1070710 (2002).

5 Soininen, J., Kokocinski, M., Estlander, S., Kotanen, J. & Heino, J. Neutrality, niches, and determinants of plankton metacommunity structure across boreal wetland ponds. *Écoscience* **14**, 146-154, doi:10.2980/1195-6860(2007)14[146:NNADOP]2.0.CO;2 (2007).

6 Cermeno, P. & Falkowski, P. G. Controls on diatom biogeography in the ocean. *Science* **325**, 1539-1541, doi:10.1126/science.1174159 (2009).

7 Soininen, J. A quantitative analysis of species sorting across organisms and ecosystems. *Ecology* **95**, 3284-3292, doi:10.1890/13-2228.1 (2014).

8 Cottenie, K. Integrating environmental and spatial processes in ecological community dynamics. *Ecology Letters* **8**, 1175-1182, doi:10.1111/j.1461-0248.2005.00820.x (2005).

9 Stoermer, E. F. & Smol, J. P. *The Diatoms: Applications for the Environmental and Earth Sciences*. (Cambridge University Press, 2001).

10 Hatosy, S. M. *et al.* Beta diversity of marine bacteria depends on temporal scale. *Ecology* **94**, 1898-1904, doi:10.1890/12-2125.1 (2013).

11 Langenheder, S., Berga, M., Ostman, O. & Szekely, A. J. Temporal variation of [beta]-diversity and assembly mechanisms in a bacterial metacommunity. *ISME J* **6**, 1107-1114, doi:http://www.nature.com/ismej/journal/v6/n6/suppinfo/ismej2011177s1.html (2012).

12 Chain, F. J. J., Brown, E. A., MacIsaac, H. J. & Cristescu, M. E. Metabarcoding reveals strong spatial structure and temporal turnover of zooplankton communities among marine and freshwater ports. *Diversity and Distributions* **22**, 493-504, doi:10.1111/ddi.12427 (2016).

13 Mousing, E. A., Richardson, K., Bendtsen, J., Cetinić, I. & Perry, M. J. Evidence of small-scale spatial structuring of phytoplankton alpha- and beta-diversity in the open ocean. *Journal of Ecology* **104**, 1682-1695, doi:10.1111/1365-2745.12634 (2016).

14 Piredda, R. *et al.* Diversity and temporal patterns of planktonic protist assemblages at a Mediterranean Long Term Ecological Research site. *FEMS microbiology ecology* **93**, fiw200-fiw200, doi:10.1093/femsec/fiw200 (2017).

15 Chust, G., Irigoien, X., Chave, J. & Harris, R. P. Latitudinal phytoplankton distribution and the neutral theory of biodiversity. *Global Ecology and Biogeography* **22**, 531-543, doi:10.1111/geb.12016 (2013).

16 Farjalla, V. F. *et al.* Ecological determinism increases with organism size. *Ecology* **93**, 1752-1759, doi:10.1890/11-1144.1 (2012).

17 Jonsson, B. F. & Watson, J. R. The timescales of global surface-ocean connectivity. *Nat Commun* **7**, doi:10.1038/ncomms11239 (2016).

REVIEWERS' COMMENTS:

Reviewer #1 (Remarks to the Author):

The authors have improved the discussion in being more balanced towards alternative underlying mechanisms. Overall this is a nice contribution, highlighting the importance of hydrological conectivity across large spatial scales for diversity pattern in (very) small organisms.

Final round of response to the reviewer's comments

Manuscript No.: NCOMMS-16-24484 entitled "***Large scale ocean connectivity and planktonic body size***"

- Reviewer comment (in italic and grey)

- Author Response (AR) in blue

REVIEWERS' COMMENTS:

Reviewer #1 (Remarks to the Author):

"The authors have improved the discussion in being more balanced towards alternative underlying mechanisms. Overall this is a nice contribution, highlighting the importance of hydrological connectivity across large spatial scales for diversity pattern in (very) small organisms".

AR: We are thankful to the reviewer for his/her positive assessment.
